# Enhanced external counterpulsation ameliorates endothelial dysfunction and elevates exercise tolerance in patients with coronary artery disease

**DOI:** 10.3389/fcvm.2022.997109

**Published:** 2022-11-29

**Authors:** Huongrui Yang, Lixue Song, Xiang Ning, Yanyan Ma, Aiying Xue, Hongbing Zhao, Yimeng Du, Qinghua Lu, Zhendong Liu, Juan Wang

**Affiliations:** ^1^Department of Cardiology, The Second Hospital of Shandong University, Jinan, Shandong, China; ^2^Cardio-Cerebrovascular Control and Research Center, Basic Medical College, Shandong First Medical University, Jinan, Shandong, China

**Keywords:** enhanced external counterpulsation, hemodynamic responses, cardiopulmonary exercise tests, metabolic equivalent (METs), endothelial growth factors

## Abstract

**Purpose:**

Enhanced external counterpulsation (EECP) is a new non-drug treatment for coronary artery disease (CAD). However, the long-term effect of EECP on endothelial dysfunction and exercise tolerance, and the relationship between the changes in the endothelial dysfunction and exercise tolerance in the patients with coronary heart disease are still unclear.

**Methods:**

A total of 240 patients with CAD were randomly divided into EECP group (*n* = 120) and control group (*n* = 120). All patients received routine treatment of CAD as the basic therapy. Patients in the EECP group received 35 1-h daily sessions of EECP during 7 consecutive weeks while the control group received the same treatment course, but the cuff inflation pressure was 0–10 mmHg. Peak systolic velocity (PSV), end diastolic velocity (EDV), resistance index (RI), and inner diameter (ID) of the right carotid artery were examined using a Color Doppler Ultrasound and used to calculate the fluid shear stress (FSS). Serum levels of human vascular endothelial cell growth factor (VEGF), vascular endothelial cell growth factor receptor 2 (VEGFR_2_), and human angiotensin 2 (Ang_2_) were determined by enzyme-linked immunosorbent assay (ELISA). Exercise load time, maximal oxygen uptake (VO_2*max*_), metabolic equivalent (METs), anaerobic threshold (AT), peak oxygen pulse (VO_2max_/HR) were assessed using cardiopulmonary exercise tests.

**Results:**

After 1 year follow-up, the EDV, PSV, ID, and FSS were significantly increased in the EECP group (*P* < 0.05 and 0.01, respectively), whereas there were no significant changes in these parameters in the control group. The serum levels of VEGF and VEGFR_2_ were elevated in the EECP and control groups (all *P* < 0.05). However, the changes in VEGF and VEGFR_2_ were significantly higher in the EECP group than in the control group (*P* < 0.01). The serum level of Ang_2_ was decreased in the EECP group (*P* < 0.05) and no obvious changes in the control group. As for exercise tolerance of patients, there were significant increases in the exercise load time, VO2_max_, VO_2max_/HR, AT and METs in the EECP group (all *P* < 0.05) and VO_2max_ and METs in the control group (all *P* < 0.05). Correlation analyses showed a significant and positive correlations of VEGF and VEGFR_2_ levels with the changes in FSS (all *P* < 0.001). The correlations were still remained even after adjustment for confounders (all *Padjustment* < 0.001). Linear regression displays the age, the medication of ACEI (angiotensin-converting enzyme inhibitors) or ARB (angiotensin receptor blockers), the diabetes and the changes in VEGF and VEGFR_2_ were positively and independently associated with the changes in METs after adjustment for confounders (all *Padjustment* < 0.05).

**Conclusion:**

The data of our study suggested that EECP is a useful therapeutic measurement for amelioration of endothelial dysfunction and long-term elevation of exercise tolerance for patients with coronary heart disease.

**Clinical trial registration:**

[http://www.chictr.org.cn/], identifier [ChiCTR1800020102].

## Introduction

Many clinical and comparative studies have confirmed that EECP is a safe, effective, non-invasive, and cost-effective treatment method for coronary artery disease (CAD). In 2013, the European Society of Cardiology included EECP therapy in the treatment plan for patients with stable coronary heart disease. External counterpulsation was recently shown to significantly reduce the incidence of angina pectoris in patients with CAD, increase myocardial perfusion, and improve left ventricular function (1, 2).

By synchronizing a patient’s electrocardiogram signal and cycle, the compression cuff wrapped around the patient’s calf and thigh is continually expanded and swollen to enlarge the coronary arteries. The perfusion pressure increases the cardiac output and reduces the left ventricular load (3). By establishing a three-dimensional model, the fundamental principle of the EECP in the treatment of CAD is the improvement of the hemodynamics (4). Other study reported that, after a month of follow-up, the positive effects of EECP persist. This suggests that improving endothelial function may contribute to the clinical benefit of EECP for patients with symptomatic CAD (5).

Endothelial dysfunction is a systemic process, not necessarily limited to the clinically visible atherosclerotic vascular layer (6). Patients with coronary heart disease often have varying degrees of endothelial dysfunction. Study has predominantly focused on EECP improving myocardial perfusion (1) and changing hemodynamics (7), however, there are few studies on endothelial function. In the current study, it was hypothesized that EECP would induce changes in blood flow shear stress resulting in corresponding changes in endothelial-derived vasoactive substances, blood pressure, and cardiopulmonary function. Through the randomized controlled trial method, 240 patients with coronary atherosclerotic heart disease were enrolled, their background information was recorded, and they received standard EECP and medication. Cardiopulmonary exercise tests were performed before starting EECP treatment and after 1 year of follow-up. Hematology specimens were collected from the patients for further analysis.

## Materials and methods

### Participants

A total of 240 patients with coronary atherosclerotic heart disease that had been diagnosed by coronary angiography were enrolled in the study. Exclusion criteria were absence of ST-segment depression (at least 1 mm) in the cardiopulmonary exercise testing (CPET), >75 years of age, coronary artery bypass graft within the past 3 months or PCI in the past 6 months, uncontrolled arrhythmia, decompensated heart failure, severe heart valve disease, severe peripheral vascular disease, uncontrolled hypertension or severe pulmonary hypertension, pregnancy, aneurysms requiring surgery, and other situations that are not suitable for EECP. This study was approved by the Shandong University Institutional Review Board and written informed consent was obtained from all patients. The study was registered with ChiCTR.org.cn (ChiCTR18000201002).

### Intervention

Patients were randomly divided into EECP and control groups, the ratio of EECP group to control group was 1:1. Patients in the EECP group received 35 1-h daily sessions of EECP over 7 consecutive weeks, using cuff inflation pressures of 300 mmHg, while the control group received the same treatment course, but the cuff inflation pressure was 0–10 mmHg. All patients received essential medication for CAD, which included antiplatelet drugs (aspirin 100 mg/day and/or clopidogrel 75 mg/day), β-receptor blockers, angiotensin-converting enzyme inhibitors (ACEI) or angiotensin receptor blockers (ARB) and statins.

### Biochemical assays

Serum was collected from control and experimental groups before starting EECP treatment and after 1 year of follow-up. Serum levels of human vascular endothelial cell growth factor (VEGF), human vascular endothelial cell growth factor receptor 2 (VEGFR_2_), and human angiotensin 2 (Ang_2_) were determined by enzyme-linked immunosorbent assay (ELISA) kit (Cusabio, Wuhan, China). Blood lipids, creatinine, biochemical ions, fasting plasma glucose, and other indicators were measured by standard biochemical methods on a Cobas c702 platform.

### Carotid ultrasound

Color Doppler Ultrasound (Sonosite M-Turbo, Bothell, WA, USA) was used to obtain hemodynamic parameters of the right carotid artery at baseline and 1 year after 35 sessions of treatment of the EECP and non-EECP groups. Parameters including peak systolic velocity (PSV), end diastolic velocity (EDV), resistance index (RI), and inner diameter (ID) were examined at a distance of 1.5 cm proximally to the bifurcation of the vessel. FSS is required for normal homeostasis of endothelial function (8). So, we further calculated the change in FSS before and after the intervention. According to Poiseuille’s law, the formula for calculating blood fluid shear stress (FSS) was as follows: FSS = η × 4 × V/D, where η is blood viscosity, V is mean blood flow velocity, and D is end-diastolic vessel diameter (9). The baseline status measurements of both groups were performed in the supine position after 10 min of relaxation. For the EECP group, the measurement after treatment was the same as baseline measurements. For the non-EECP group, measurements were performed soon after the final EECP treatment. Differences between the before and after data were calculated. The original ultrasound image and data has been uploaded to the Supplementary material.

### Cardiopulmonary exercise testing

All patients underwent CEPT with 12-lead ECG monitoring (Mac 5000, GE Healthcare, Waukesha, WI) before the intervention and 1 year after follow-up. Exercise load time, maximal oxygen uptake (VO_2max_), metabolic equivalent (METs), anaerobic threshold (AT), peak oxygen pulse (VO_2max_/HR), and other indicators were calculated and the values before and after intervention were compared. AT was determined by the V-slope method (10). VO_2max_ was determined as the average value during the final 30 s of exercise. Exercise load time refers to the time to reach the stop target. VO_2max_/HR is the VO_2max_ divided by the heart rate concurrently. METs were estimated as the ratio of resting metabolic rate where 1 MET is equivalent to the energy expenditure value of sitting quietly using an established method (11).

### Statistical analysis

Categorical variables were expressed in numbers and percentages and compared using the Chi-square test. Continuous data are expressed as mean ± standard deviation (SD) or median [interquartile range (IQR)]. The Shapiro–Wilk test was used to test normality. Comparisons the changes in EECP and control groups were made with the independent *t*-test. The paired *t*-test was used for comparison before and after EECP treatment. Spearman’s correlation was employed to assess the correlation between variables. Multiple linear regression analysis was performed to evaluate the independent risk factors and the best predictor for exercise load time, VO_2max_, METs, AT, and VO_2max_/HR. Statistical analyses were performed using SPSS software (version 20) and GraphPad Prism Software (version 9.0.0).

## Results

The descriptive and metabolic characteristics of the patients are shown in Table 1. All patients completed the experimental protocol of the EECP treatment or control regimen, and no adverse cardiovascular events occurred. There were no significant differences in baseline characteristics between the intervention and control group (all *P* > 0.05).

**TABLE 1:** Baseline information in the two groups.

Variables	Control group (*n* = 120)	EECP group (*n* = 120)	*P-*value
Age (years)	61.89 ± 7.57	63.11 ± 7.47	0.269
Sex [Female, *n* (%)]	83 (69.17)	79 (65.83)	0.581
Smoking *n* (%)	20 (16.67)	15 (12.50)	0.360
Prior PTCA	0 (0.00)	1 (0.83)	0.316
Prior CABG	21 (17.50)	17 (14.16)	0.479
Hypertension	27 (22.50)	33 (27.50)	0.371
Diabetes mellitus	26 (21.67)	23 (19.17)	0.631
Hyperlipidemia	3 (2.50)	6 (5.00)	0.308
Antiplatelet drugs	120 (100)	120 (100)	0.999
β-blocker	23 (19.17)	18 (15.00)	0.391
Lipid-lowering drug	120 (100)	120 (100)	0.999
ACEI/ARB	26 (21.67)	30 (25.00)	0.542
Insulin	22 (18.33)	18 (15.00)	0.488

Age is expressed as mean ± SD. Categorical variables are expressed as a percentage. PTCA, percutaneous transluminal coronary angioplasty; CABG, coronary artery bypass grafting; ACEI, angiotensin-converting enzyme inhibitors; ARB, angiotensin receptor blocker.

### Effect of enhanced external counterpulsation on hemodynamics

**FIGURE 1:**
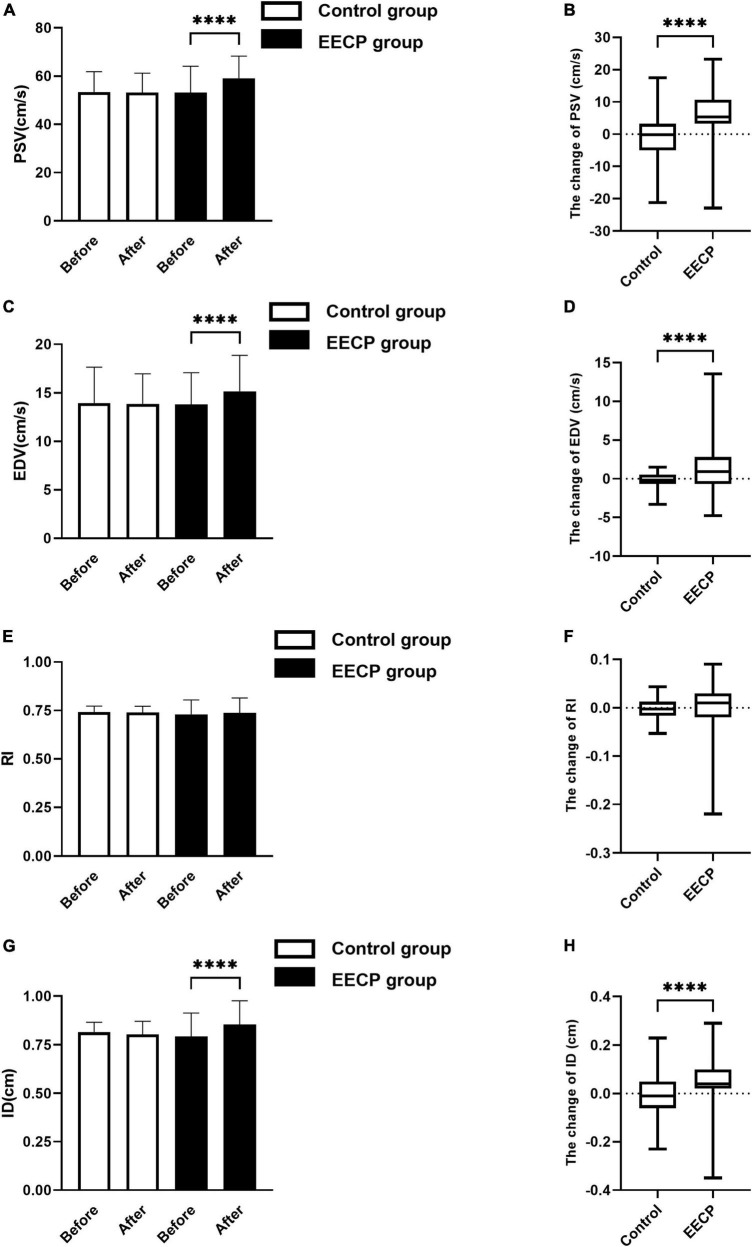
The change of carotid blood flow status before and after the intervention. Effect of interventions on the PSV **(A)**, EDV **(C)**, RI **(E)**, and ID **(G)** of the right carotid artery (RC) between the EECP and control groups. Change of PSV **(B)**, EDV **(D)**, RI **(F)**, and ID **(H)** before and after the intervention between the EECP and control groups. PSV, peak systolic velocity; EDV, end diastolic velocity; RI, resistance index; ID, inner diameter. “****” denotes *P* < 0.001.

No significant differences in PSV, EDV, RI, and ID were found between the EECP group and the control group before intervention (all *P* > 0.05, Figure 1). After the intervention, EDV (*P* < 0.05, Figure 1A) and PSV (*P* < 0.01, Figure 1C) in the EECP group were significantly higher compared with that before intervention, while the ID in the EECP group was increased slightly compared with pre-intervention levels (*P* < 0.01, Figure 1G). EDV, PSV, and ID increased more in the EECP group compared with the control group (all *P* > 0.05, Figures 1B,D,H). However, there was no significant difference in the RI of the right carotid artery in the EECP group before and after intervention (*P* > 0.05, Figure 1E), and no significant difference in the change value of RI in the EECP group compared with the control group (*P* > 0.05, Figure 1F). There were no significant differences in EDV, PSV, RI, ID, and FSS in the control group before and after intervention (all *P* > 0.05, Figures 1A,C,E,G).

**FIGURE 2:**
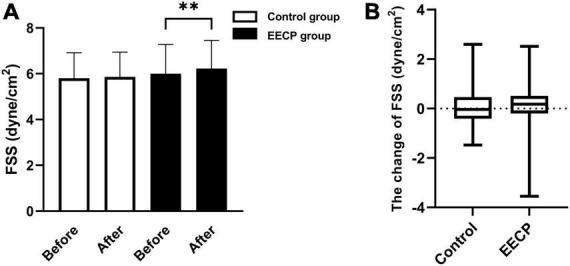
The change of FSS before and after the intervention. Effect of interventions on the FSS **(A)** of the right carotid artery (RC) between the EECP and control groups. Change of FSS **(B)** before and after the intervention between the EECP and control groups. FSS, fluid shear stress. “**” denotes *P* < 0.01.

Fluid shear stress is required for normal homeostasis of endothelial function (8). In Figure 2, we compared the changes in FSS between the control group and the EECP group. No significant difference in FSS was found between the EECP group and the control group before intervention (*P* > 0.05), but there was significant increase in FSS in the EECP group after intervention (*P* < 0.01).

### Effect of enhanced external counterpulsation on endothelial function

**FIGURE 3:**
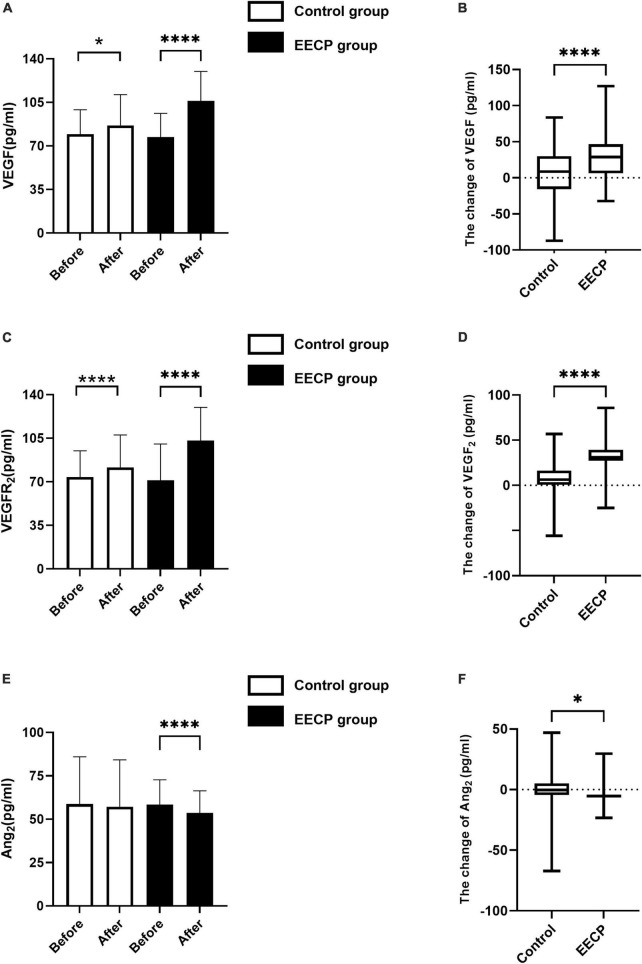
The change of endothelial cell cytokine before and after the intervention. Effect of interventions on expression of VEGF **(A)**, VEGFR_2_ **(C)**, and Ang_2_ **(E)** between the EECP and control groups. Change of VEGF **(B)**, VEGFR_2_ **(D)**, and Ang_2_ **(F)** before and after the intervention between the EECP and control groups. FSS, fluid shear stress; VEGF, vascular endothelial cell growth factor; VEGFR_2_, human vascular endothelial cell growth factor receptor 2; Ang_2_, human angiotensin 2. “*” and “****” denotes P < 0.05 and <0.001.

At study entry, the levels of VEGF, VEGFR_2_, and Ang_2_ did not differ between groups (all *P* > 0.05, Figure 3). EECP therapy increased the expression levels of VEGF (*P* < 0.001, Figure 3A) and VEGFR_2_ (*P* < 0.001, Figure 3C), and simultaneously reduced the expression level of Ang_2_ (*P* > 0.05, Figure 3E). Medical treatment alone also increased the levels of VEGF (*P* < 0.001, Figure 3C) and VEGFR_2_ (*P* < 0.001, Figure 3E), but the change was more pronounced in the EECP group (*P* < 0.001, Figures 3B,D). There was no change in the expression level of Ang_2_ in the control group (all *P* > 0.05, Figure 3E).

### Effect of enhanced external counterpulsation on metabolic equivalents

At study entry, the levels of exercise load time, VO_2max_, VO_2max_/HR, AT, and METs did not differ between groups (all *P* > 0.05, Table 2). After 1 year of follow-up, the exercise load time, VO_2max_, VO_2max_/HR, AT, and METs showed a significant increase in the EECP group compared with the value’s pre-intervention, and the difference was statistically significant (all *P* < 0.05, Table 2). VO_2max_ (*P* = 0.03, Table 2) and METs (*P* = 0.001, Table 2) of the control group were also higher compared with baseline levels. In the control group, there was no change in the exercise load time, AT, and VO_2max_/HR from the baseline levels (all *P* > 0.05, Table 2).

**TABLE 2:** Results of cardiopulmonary exercise testing before and after intervention therapy.

	Control group			EECP group			*P*-value*
	T1	T2	*P*-value	T1	T2	*P*-value	
Exercise load time (s)	416.32 ± 55.92	419.96 ± 81.19	0.143	417.00 ± 84.53	455.14 ± 102.15	< 0.001	0.942
VO_2max_ (ml/min)	1144.93 ± 350.80	1158.79 ± 322.80	0.030	1183.79 ± 330.20	1423.47 ± 298.15	< 0.001	0.378
AT (ml/min/kg)	31.32 ± 3.35	31.18 ± 2.55	0.092	31.40 ± 4.32	36.25 ± 4.77	< 0.001	0.876
VO_2_/HR_max_ (ml/beat)	10.08 ± 2.93	10.38 ± 2.94	0.412	9.93 ± 2.39	11.43 ± 1.64	< 0.001	0.664
METs	4.53 ± 1.04	5.11 ± 1.20	< 0.001	4.47 ± 0.86	5.25 ± 1.20	0.001	0.606

*P*-values were calculated by paired *t*-test within a group after 35 sessions of EECP. *P*-value* is paired *t*-test by between groups in baseline. T1, time 1 (baseline); T2, time 2 (after 35 sessions of EECP); VO_2max_, maximal oxygen uptake; METs, metabolic equivalent; AT, anaerobic threshold; VO_2max_/HR, oxygen pulse.

### Multiple linear regression analysis

**FIGURE 4:**
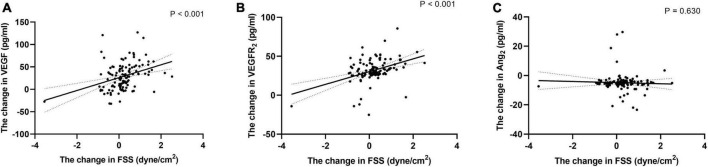
Correlation of the change of FSS with the change of endothelial cell cytokine. Correlation of the change of FSS during follow-up with the change of VEGF **(A)**, VEGFR_2_ **(B)**, and Ang_2_ **(C)**. Exponential trend lines are included on each graph. FSS, fluid shear stress; VEGF, vascular endothelial cell growth factor; VEGFR_2_, vascular endothelial cell growth factor receptor 2; Ang_2_, angiotensin 2.

Differences in carotid artery ultrasound data, endothelial cytokines and METs before and after intervention were calculated and these new variables were represented by Δ plus the original variable name. Figure 4 showed correlation analysis showed that ΔFSS was positively correlated with VEGF and VEGFR_2_ (*r* = 0.356 and 0.407, respectively, all *P* < 0.001), and no significant correlation exists between ΔAng_2_ (*r* = 0.044, *P* = 0.630). The correlations were still remained even after adjustment for confounders (all *Padjustment* < 0.001).

**FIGURE 5:**
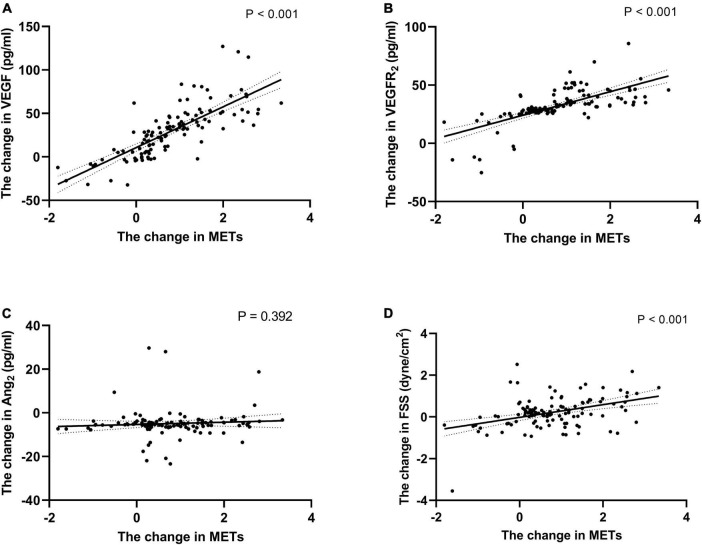
Correlation of the change of METs with the change of endothelial cell cytokine and FSS. Correlation of the change of METs during follow-up with the change of VEGF **(A)**, VEGFR_2_ **(B)**, Ang_2_ **(C)** and FSS **(D)**. Exponential trend lines are included on each graph. FSS, fluid shear stress; VEGF, vascular endothelial cell growth factor; VEGFR_2_, human vascular endothelial cell growth factor receptor 2; Ang_2_, human angiotensin 2.

There were positive correlations between ΔFSS, ΔVEGF, ΔVEGFR_2_ and ΔMETs (*r* = 0.409, 0.660, and 0.485, respectively, all *P* < 0.001, Figure 5). No significant correlation exists between ΔAng_2_ and ΔMETs and ΔFSS (*P* > 0.05, Figure 5). ΔExercise loadtime, ΔVO_2_max, ΔVO_2max_/HR, ΔAT, and ΔMETs were used as dependent variables, respectively, and the clinical variables related to these variables in Spearman’s correlation analysis were used as independent variables. Potential confounders such as gender (male = 1, female = 0), smoking history (No = 0, Yes = 1), medication history, and previous medical history (No = 0, Yes = 1) were also included as independent variables. Multivariate stepwise regression analysis was performed on the data of 120 patients in the EECP group, and regression equations were established. Table 3 shows that age, use of ACEI or ARB, ΔVEGF, ΔVEGFR_2_, and the history of diabetes were associated with the levels of METs over the EECP treatment period. VIFs were < 1.5 in all analyses, suggesting that no collinearity was observed.

**TABLE 3:** Multiple linear regression of influencing factors of ΔMETs.

Variable	Unstandardized β	95% CI	*t*-value	*P*-value
Age (years)	–0.034	−0.051 to −0.017	–3.978	< 0.001
ACEI or ARB	0.300	0.059 to 0.542	2.485	0.015
Diabetes	0.273	0.030 to 0.517	2.225	0.028
ΔVEGF (pg/ml)	0.019	0.014 to 0.023	8.465	< 0.001
ΔVEGFR_2_ (pg/ml)	0.014	0.004 to 0.023	2.967	0.023

Δ indicates the change value. VEGF, vascular endothelial cell growth factor; VEGFR_2_, vascular endothelial cell growth factor receptor 2; ACEI, angiotensin-converting enzyme inhibitors; ARB, angiotensin receptor blocker.

## Discussion

There was different degrees of endothelial dysfunction in patients with coronary heart disease and endothelial dysfunction is a systemic process that is not necessarily limited to the atherosclerotic vascular layer (6). Although elective PCI was used for patients with ischemic heart disease, the progression of coronary atherosclerosis, stent restenosis, coronary endothelial dysfunction, and coronary microcirculation disorders may be unpredictable. The symptoms of CAD in the elderly patients are often atypical, and the incidence of asymptomatic myocardial ischemia, asymptomatic coronary artery spasm, and coronary microangiopathy is higher than the non-elderly patients (12). EECP affected the patient’s blood pressure, changed the vascular endothelial shear force, and then promoted the formation of collateral circulation, increased organ perfusion and improved cardiopulmonary function.

Our study found that the PSV and EDV increased significantly during EECP treatment in patients with coronary heart disease and resulted in increased carotid vascular shear (7, 13). Internal carotid artery blood flow velocity changed rapidly during systole and slowly during diastole, and PSV could return to normal levels immediately after EECP. The blood flow status of the right carotid artery changed more significantly compared with that of the left carotid artery (13). Two studies had confirmed that changes in the blood flow characteristics of the right common carotid artery were more predictive of cardiovascular events compared with those of the left common carotid artery (14, 15). In the current study, the RI of the right carotid artery was significantly lower in patients with coronary heart disease immediately after EECP compared with the baseline. Decreased RI suggests improved peripheral vascular function (16), and was an important manifestation of the improvement of carotid hemodynamics in patients with coronary heart disease. Changes in RI could predict the degree of atherosclerosis, reflect cerebrovascular resistance, and helped increase cerebral blood flow (16, 17).

Vascular endothelial cell growth factor activity *in vivo* can promote the growth of arterial, venous, and lymphatic endothelial cells (18). FSS was required for normal homeostasis of endothelial function (19). FSS also activated VEGFR_2_ phosphorylation and induces its downstream signaling (8). Fluid shear stress regulates VEGFR-3 was expressed in vascular endothelial cells undergoing vascular remodeling during early pregnancy (20). Our study found that the FSS of the carotid artery increased significantly before and after a single EECP treatment, and that the FSS was positively correlated with changes in serum expression levels of VEGF and VEGFR_2_. This was consistent with the study of Steffen Gloekler (21). Impaired expression of eNOS is a crucial element for fluid shear stress regulation of endothelial function. EECP treatment increased plasma NO levels and endothelial NO synthase (eNOS) gene expression (22). In animal models, EECP could upregulate the expression of vascular eNOS and downregulate the activity of extracellular signal-regulated kinase 1/2 (ERK1/2) and inhibit the development of intimal hyperplasia and atherosclerosis (23). Changes in arterial blood flow induce a reduction of Kruppel-like factor 2 (KLF2)-mediated apoptosis and increase VEGF while activating expression of VEGF-VEGFR_2_ autocrine-paracrine signaling (24). Binding of VEGFR_2_ to VEGF-A (VEGF) induced dimerization and activation of its receptor kinase activity, leading to autophosphorylation on tyrosine residues (25). These findings indicated that changes in shear force may be an important mechanism to promote VEGF production during EECP. The formation of coronary collateral circulation (CCC) was a process involving many factors. Our experiments showed lower shear stress in patients treated with EECP fluid shear elicited a strong arteriogenic response, restores cell proliferation, and stimulates cytoskeletal rearrangements. *In vivo*, shear stress and VEGF could regulate downstream signaling pathways through the same receptor mechanism (26). VEGF has an important role in the formation of muscle ischemic arterioles caused by exercise (27–29). However, in animal models, VEGF expression gradually decreased during the later stages of collateral circulation maturation, suggesting that VEGF primarily mediates the initiation of collateral loops (30).

Experiments in the current study revealed that VEGF and VEGFR_2_ expression was significantly higher in the EECP group compared with the routine medication regimen group, indicating that external counterpulsation could promote the establishment of coronary collateral circulation and improve blood flow reserve and myocardial perfusion. Ang_2_ was one of the ligands of the tyrosine kinase receptor Tie-2. Ang_2_ bind to Tie-2 and could promote endothelial cell growth, tumor cell growth, and angiogenesis. In the present study, there was no significant change in the level of Ang_2_ in the experimental group after EECP treatment. This phenomenon was related to the use of ACEI or ARB. When endothelial cells were exposed to the low sheer force of 1 dyne/cm2 for 24 h, the expression of Ang_2_ mRNA and protein and the subsequent release is significantly increased, but the expression and release of Ang_2_ mRNA and protein was decreased in endothelial cells exposed to high shear flow (30 dyne/cm^2^) for 24 h (31). The expression of Ang_2_ was significantly increased in vascular remodeling during many diseases. As a pro-inflammatory factor, Ang_2_ can promoted vascular remodeling and increased leukocyte infiltration, promote increased expression of P- and E-selectin, and increase vascular instability (32). Ang_2_ predominantly acted on endothelial cell-specific receptor tyrosine kinase receptor-2. the VEGF and ANG_2_ acted synergistically to promote angiogenesis (33). When cells were in different environments, Ang_2_ exhibited different physiological functions, although this phenomenon had not yet been fully explored. Improved understanding of the role of Ang_2_ in tumor angiogenesis and the cooperation of VEGF and Ang_2_ will facilitated the further development of effective antiangiogenic and anticancer therapies.

Cardiopulmonary exercise testing was a combination of exercise physiology and the relationship between gas metabolism indicators and is an objective and quantitative method for evaluating cardiopulmonary reserve function (34). In healthy people, EECP improved hemodynamic status and microcirculation, increased splanchnic perfusion, increased pulmonary blood flow, and increased the pulmonary V/Q ratio, thereby improved lung oxygenation (35–37). The current study focused on patients with CAD. CPET data was collected before intervention and after 1 year follow-up in the experimental group and revealed that exercise load time, METs, VO_2max_, AT, and VO_2max_/HR all increased, while the routine medication regimens only improved VO_2max_ and METs. These data showed that the cardiopulmonary function of patients with CHD can significantly improve after EECP treatment. Regression analysis demonstrated that a single external counterpulsation could cause changes in endothelial cytokines and predict cardiopulmonary function of patients and improved prognosis through hemodynamic changes in the carotid artery, but other factors such as medicines and medical history needed to be combined with EECP. VO_2max_ reflected individual aerobic exercise capacity and cardiorespiratory reserve. CAD patients reduced the amount of exercise compared with a healthy general population, external counterpulsation could simulate the process of muscle contraction, simulate the process of exercise, improve the hemodynamic state of skeletal muscle blood vessels, promote the formation of small blood vessels in skeletal muscle, and increase exercise tolerance (38), as well as reducing muscle soreness, fatigue, and lactic acidosis caused by lactic acid build up. Thus, EECP was beneficial to improve cardiopulmonary function, exercise tolerance, and quality of life in patients with CAD. Previous studies had found no significant associations with age, sex, BMI, beta-blockers, ACEI/ARB, statins, diltiazem, or nitrates to account for differences in METs in patients after PCI (39). Through regression analysis, we found that METS value was associated with endoglin level, age, history of diabetes. May be age, diabetes and other factors affect endothelial cell function. Therefore, in the process of external counterpulsation, the changes in hemodynamic accumulation had a more direct effect on the recovery of the patient’s cardiopulmonary function, but the indirect effect of the interactions between the various components cannot be ignored.

## Conclusion

The data of our study suggested that EECP is a useful therapeutic measurement to long-term ameliorate endothelial dysfunction and elevate exercise tolerance for patients with coronary heart disease. Future research should focus on the effect of different EECP courses on cardiopulmonary function in different patients with CAD to explore personalized treatment options in EECP therapy.

## Data availability statement

The original contributions presented in this study are included in the article/Supplementary material, further inquiries can be directed to the corresponding author.

## Ethics statement

The studies involving human participants were reviewed and approved by the Shandong University Institutional Review Board. The patients/participants provided their written informed consent to participate in this study. Written informed consent was obtained from the individual(s) for the publication of any potentially identifiable images or data included in this article.

## Author contributions

HY and JW proposed the scientific problems. HY, LS, XN, and YM collected the experimental data. HY and LS processed and calculated the data. HY conducted the statistical analysis and wrote the draft manuscript. AX, YD, QL, ZL, and JW contributed to the revision and final version of the manuscript. All authors contributed to the article and approved the submitted version.
