# Supplementary figures and images for: Enhanced external counterpulsation ameliorates endothelial dysfunction and elevates exercise tolerance in patients with coronary artery disease (part 2 of 2)
Source: Front Cardiovasc Med. 2022 Nov 29;9:997109. doi: 10.3389/fcvm.2022.997109 (PMC9744945; doi:10.3389/fcvm.2022.997109)

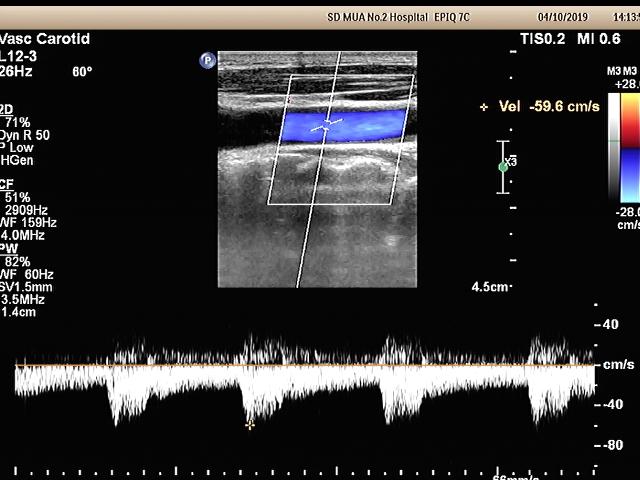

Supplement: Supplementary file 1 [file Data_Sheet_1.ZIP › CONTROLdate1/82.1.JPG]

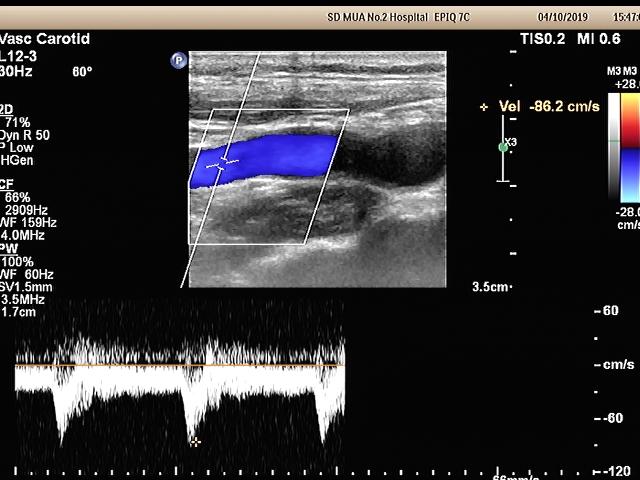

Supplement: Supplementary file 1 [file Data_Sheet_1.ZIP › CONTROLdate1/83.1.JPG]

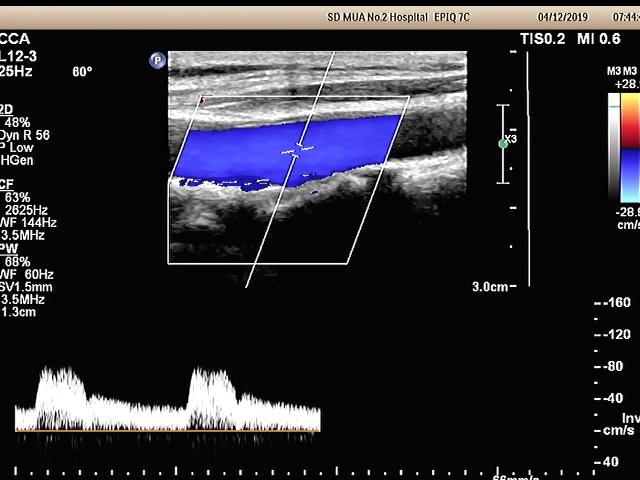

Supplement: Supplementary file 1 [file Data_Sheet_1.ZIP › CONTROLdate1/84.1.JPG]

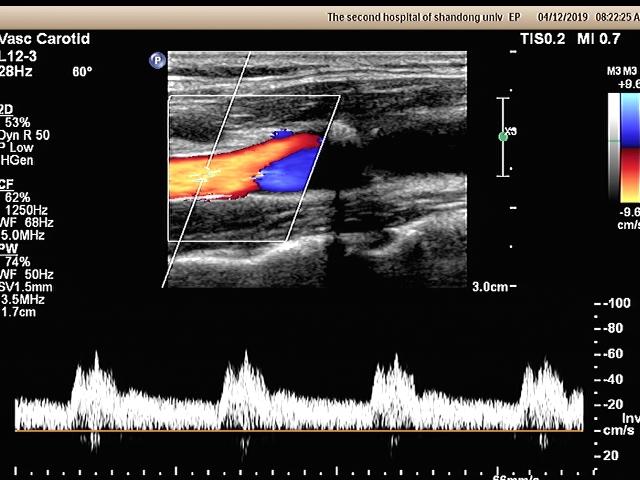

Supplement: Supplementary file 1 [file Data_Sheet_1.ZIP › CONTROLdate1/85.1.JPG]

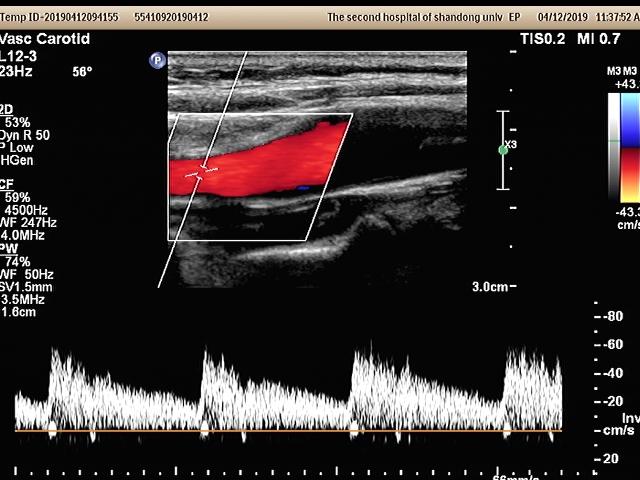

Supplement: Supplementary file 1 [file Data_Sheet_1.ZIP › CONTROLdate1/86.1.JPG]

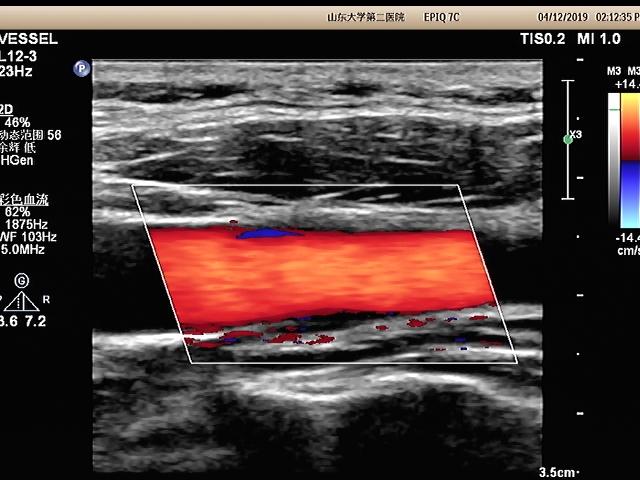

Supplement: Supplementary file 1 [file Data_Sheet_1.ZIP › CONTROLdate1/87.1.JPG]

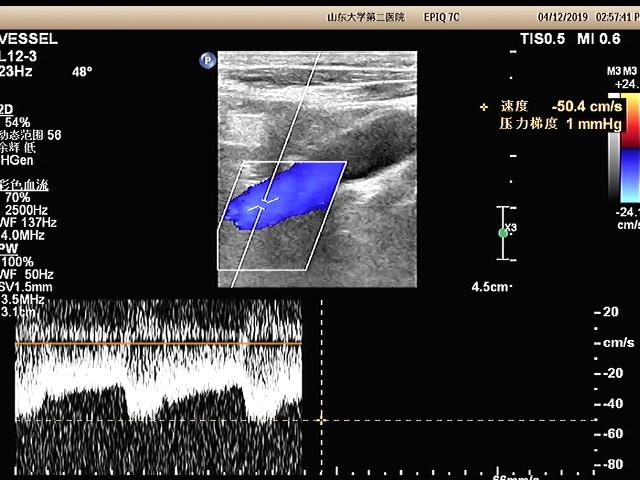

Supplement: Supplementary file 1 [file Data_Sheet_1.ZIP › CONTROLdate1/88.1.JPG]

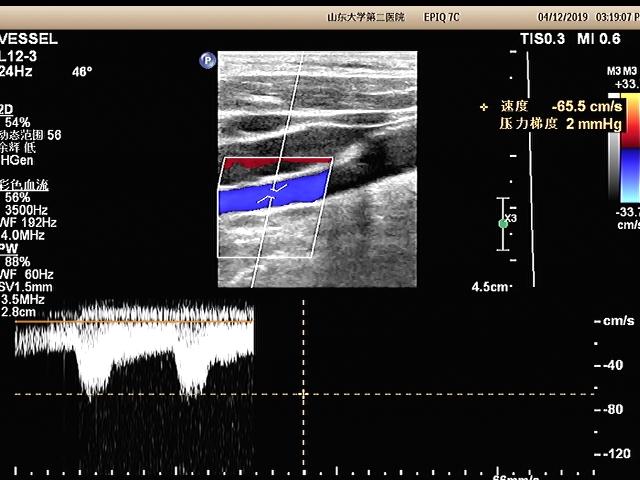

Supplement: Supplementary file 1 [file Data_Sheet_1.ZIP › CONTROLdate1/89.1.JPG]

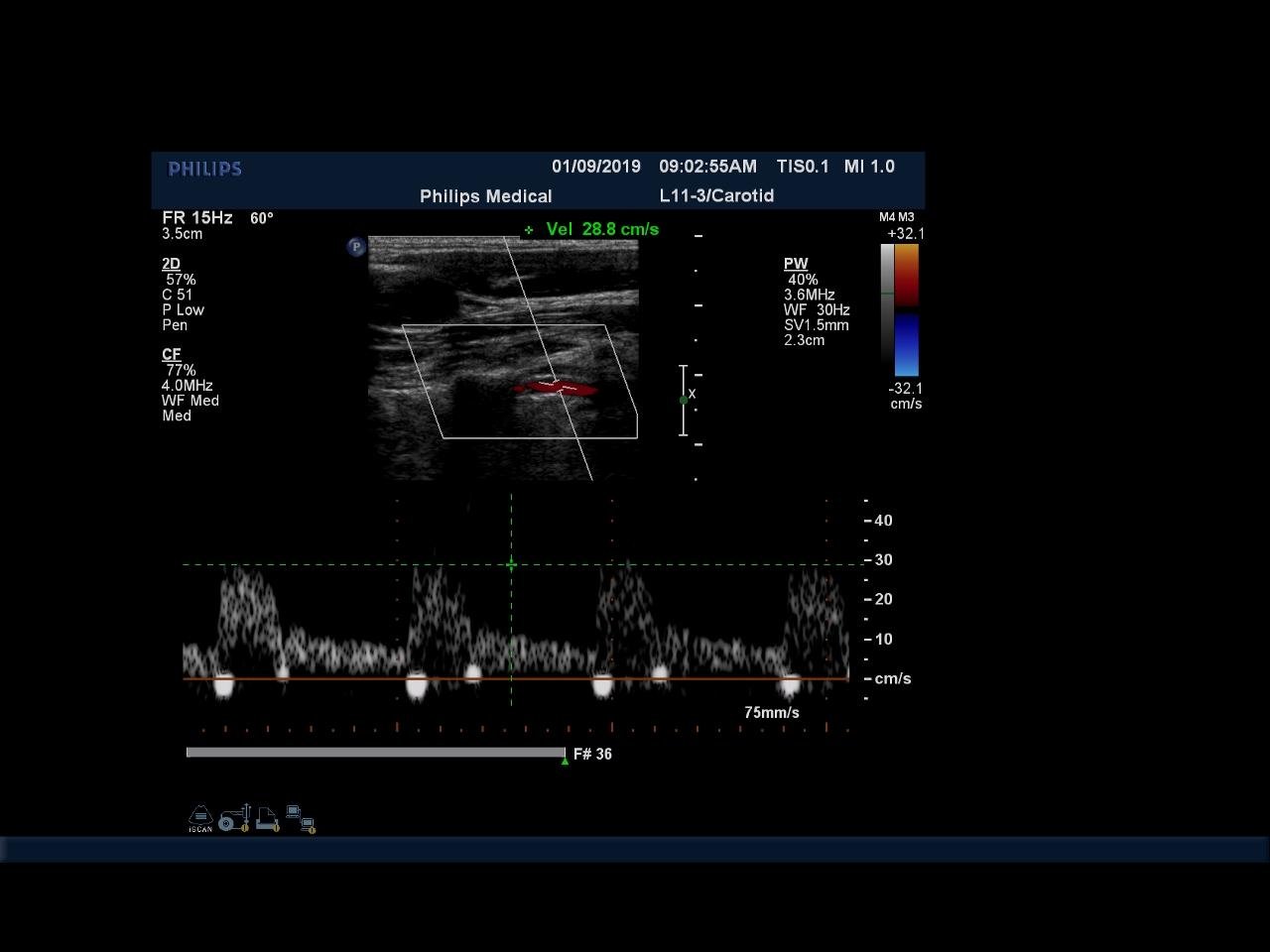

Supplement: Supplementary file 1 [file Data_Sheet_1.ZIP › CONTROLdate1/9.1.JPG]

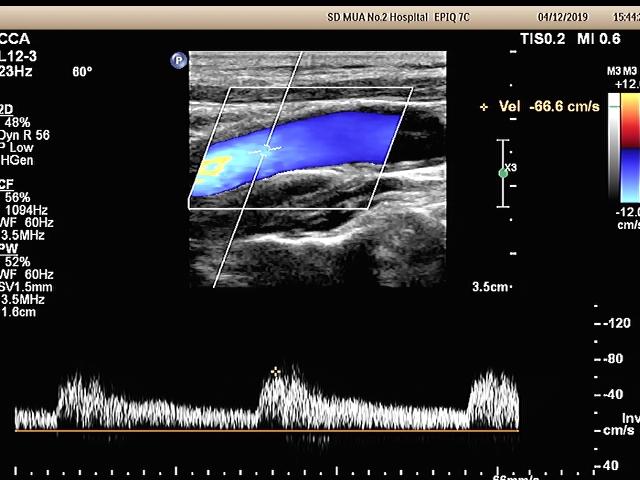

Supplement: Supplementary file 1 [file Data_Sheet_1.ZIP › CONTROLdate1/90.1.JPG]

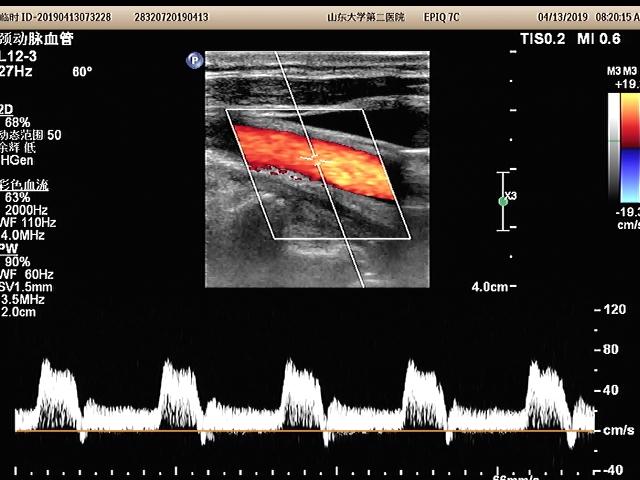

Supplement: Supplementary file 1 [file Data_Sheet_1.ZIP › CONTROLdate1/91.1.JPG]

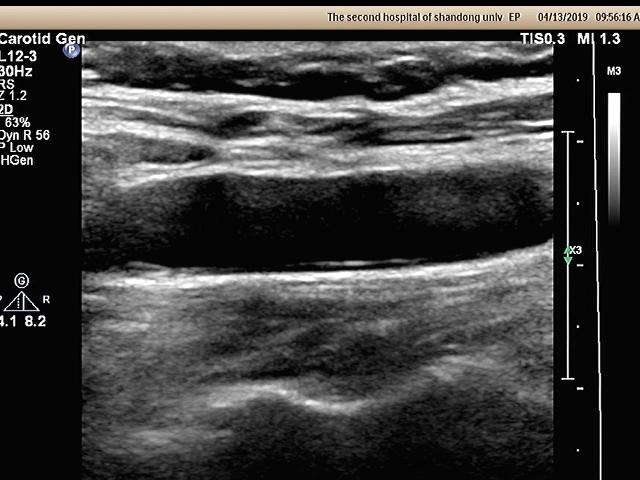

Supplement: Supplementary file 1 [file Data_Sheet_1.ZIP › CONTROLdate1/92.1.JPG]

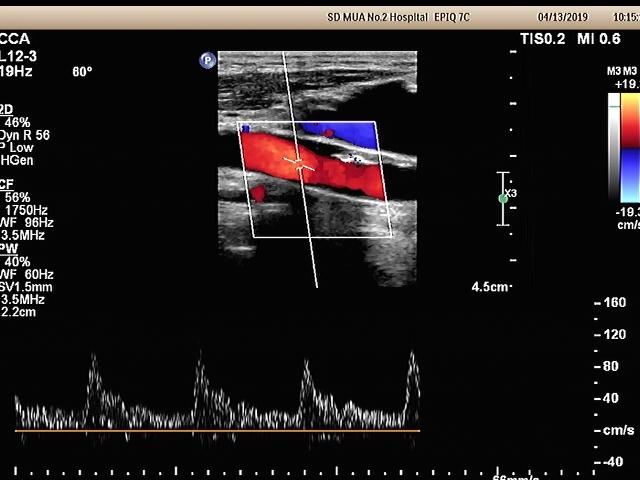

Supplement: Supplementary file 1 [file Data_Sheet_1.ZIP › CONTROLdate1/93.1.JPG]

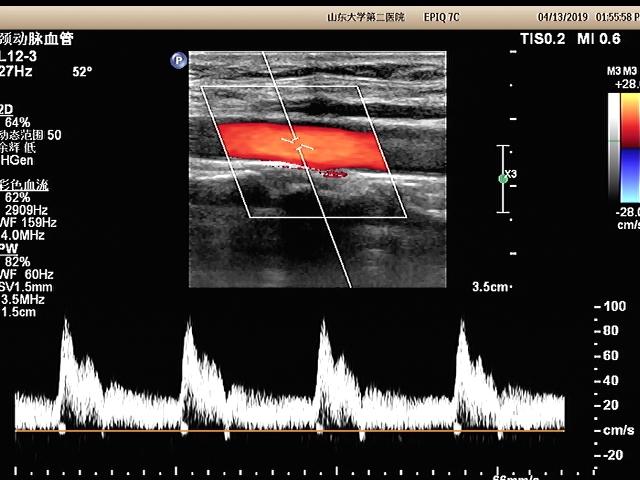

Supplement: Supplementary file 1 [file Data_Sheet_1.ZIP › CONTROLdate1/94.1.JPG]

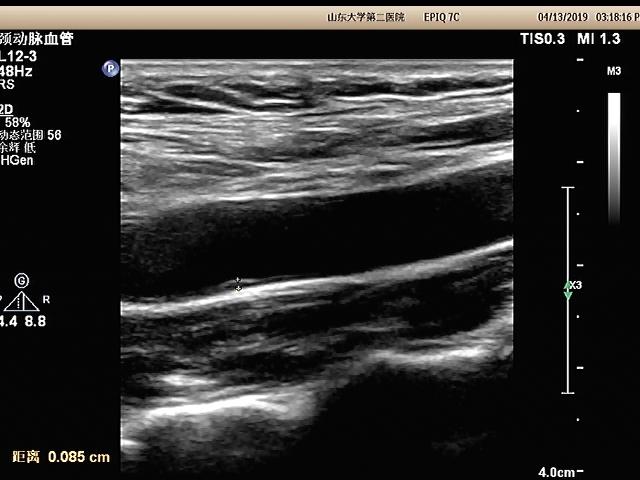

Supplement: Supplementary file 1 [file Data_Sheet_1.ZIP › CONTROLdate1/95.1.JPG]

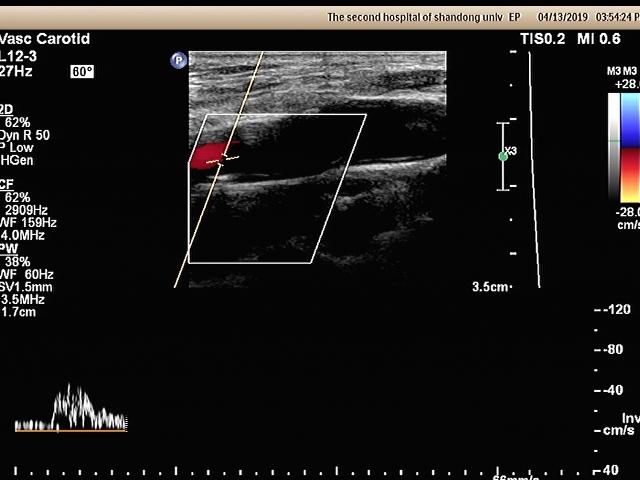

Supplement: Supplementary file 1 [file Data_Sheet_1.ZIP › CONTROLdate1/96.1.JPG]

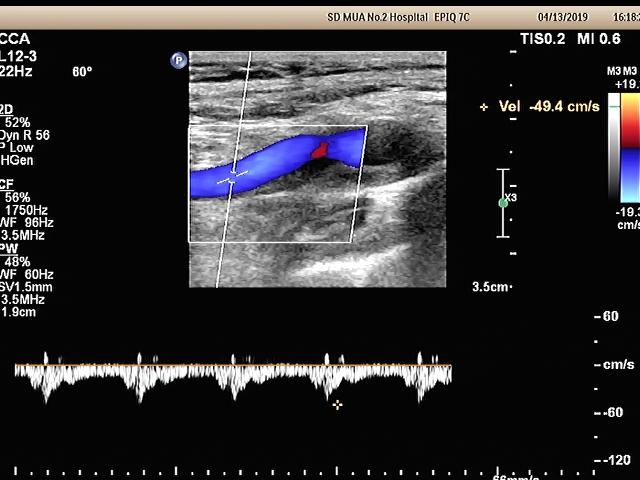

Supplement: Supplementary file 1 [file Data_Sheet_1.ZIP › CONTROLdate1/97.1.JPG]

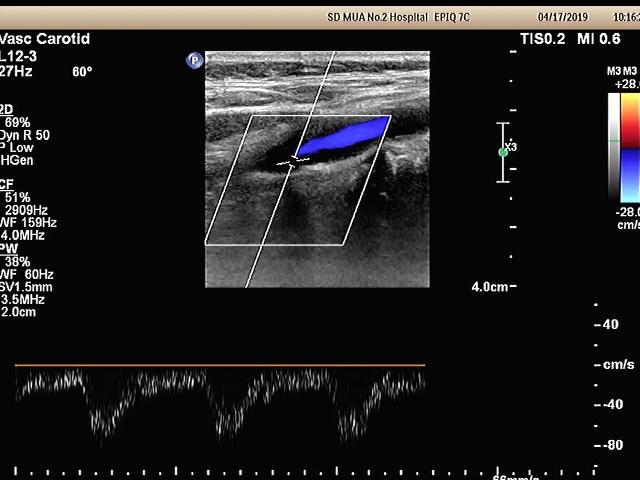

Supplement: Supplementary file 1 [file Data_Sheet_1.ZIP › CONTROLdate1/98.1.JPG]

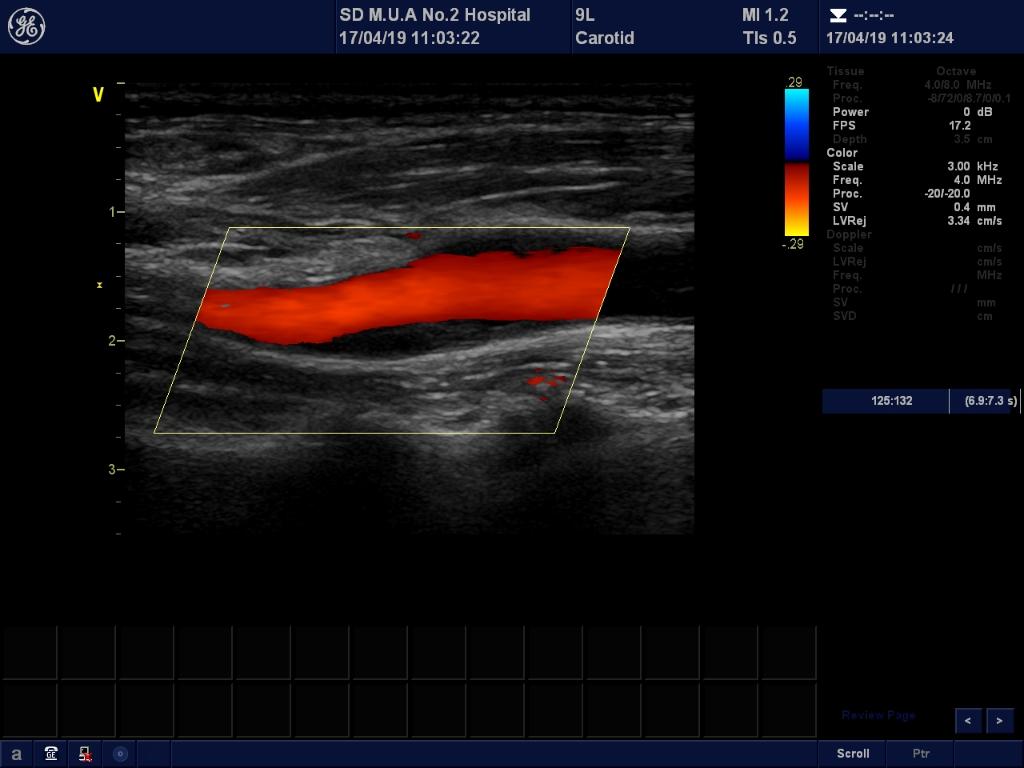

Supplement: Supplementary file 1 [file Data_Sheet_1.ZIP › CONTROLdate1/99.1.JPG]

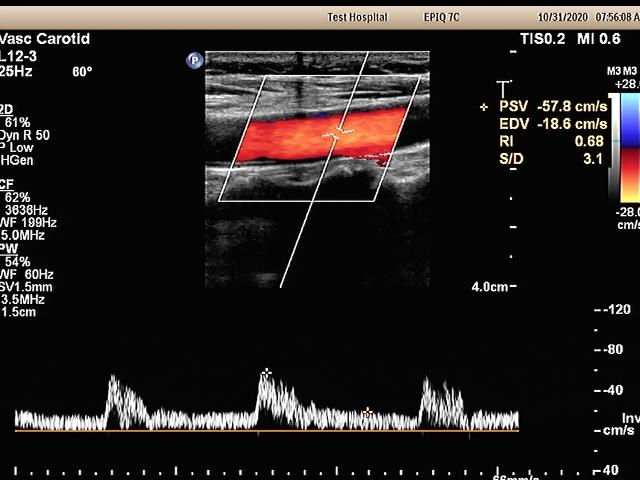

Supplement: Supplementary file 1 [file Data_Sheet_1.ZIP › CONTROLdate2/1.1.JPG]

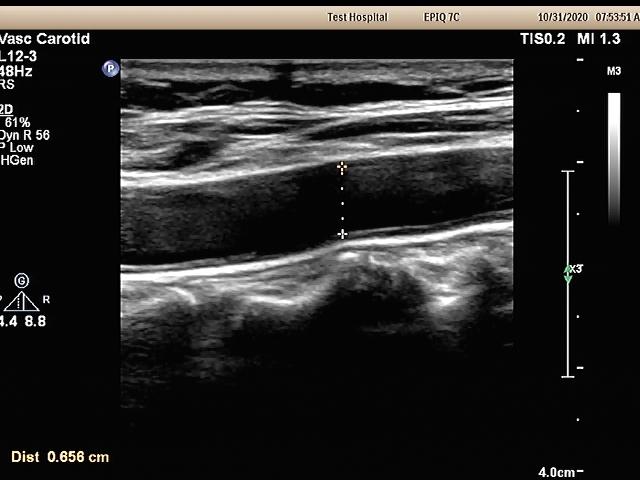

Supplement: Supplementary file 1 [file Data_Sheet_1.ZIP › CONTROLdate2/1.2.JPG]

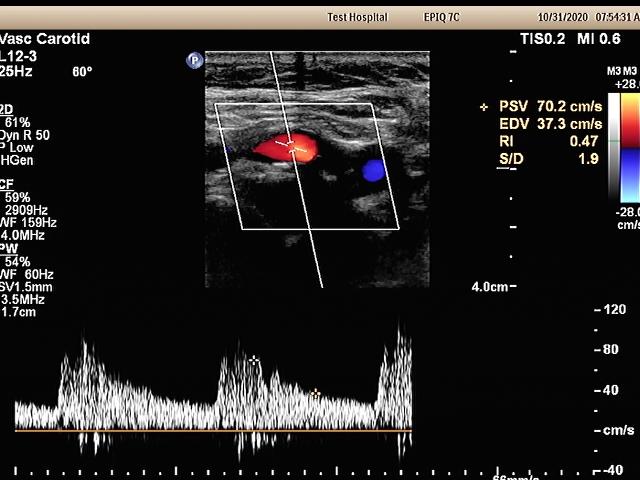

Supplement: Supplementary file 1 [file Data_Sheet_1.ZIP › CONTROLdate2/1.3.JPG]

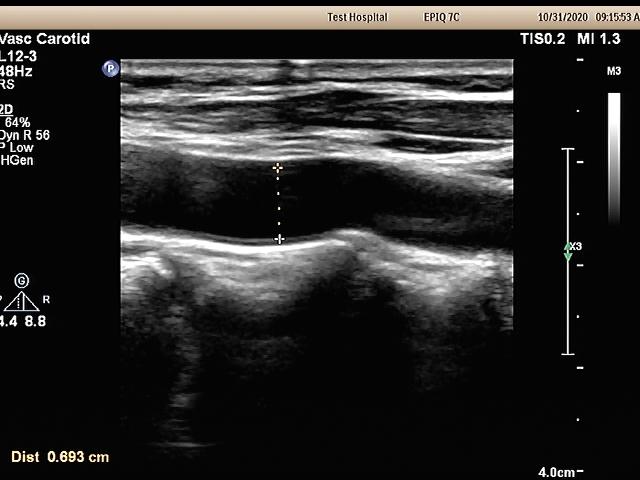

Supplement: Supplementary file 1 [file Data_Sheet_1.ZIP › CONTROLdate2/10.1.JPG]

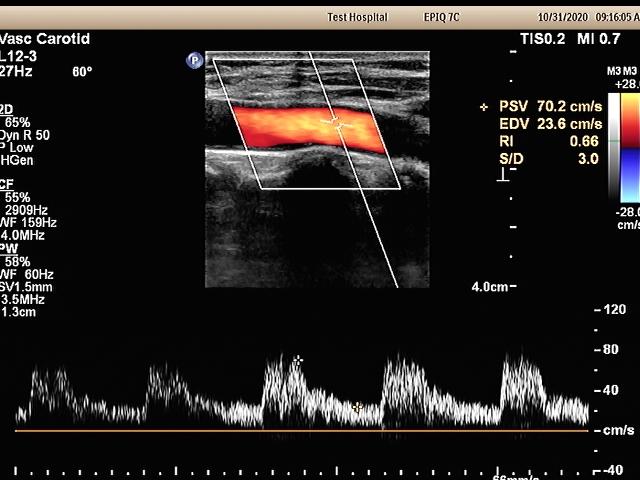

Supplement: Supplementary file 1 [file Data_Sheet_1.ZIP › CONTROLdate2/10.2.JPG]

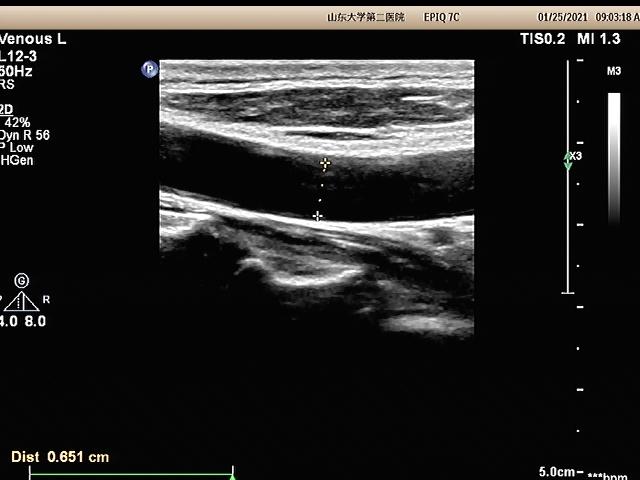

Supplement: Supplementary file 1 [file Data_Sheet_1.ZIP › CONTROLdate2/100.1.JPG]

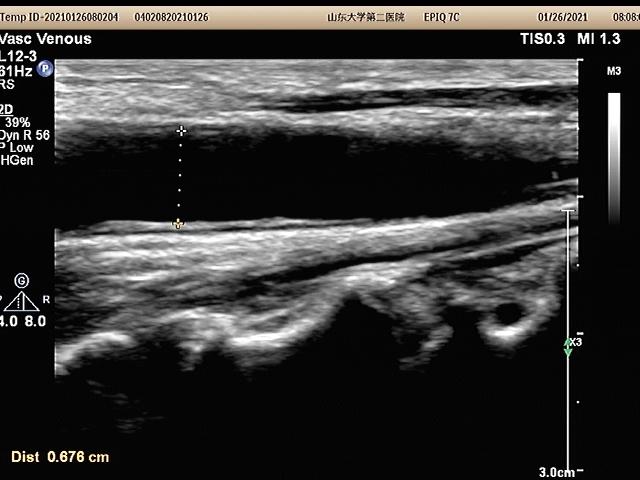

Supplement: Supplementary file 1 [file Data_Sheet_1.ZIP › CONTROLdate2/101.1.JPG]

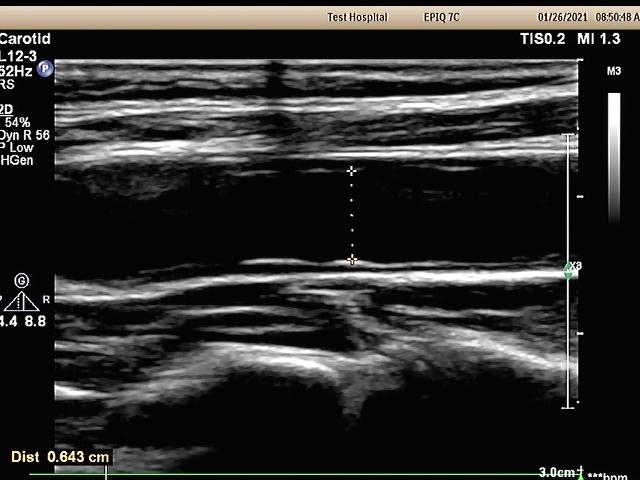

Supplement: Supplementary file 1 [file Data_Sheet_1.ZIP › CONTROLdate2/102.1.JPG]

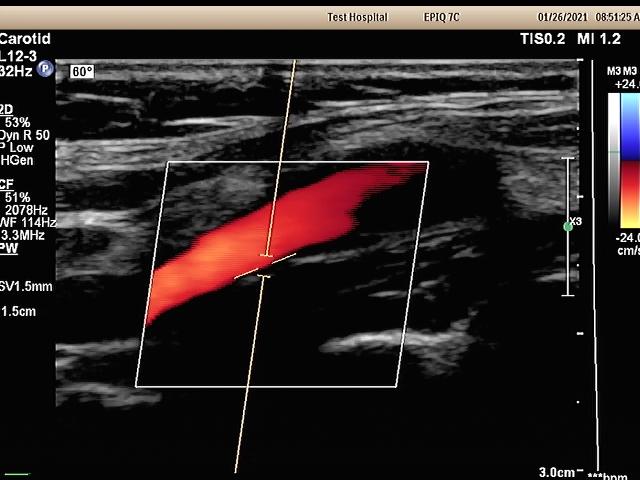

Supplement: Supplementary file 1 [file Data_Sheet_1.ZIP › CONTROLdate2/102.2.JPG]

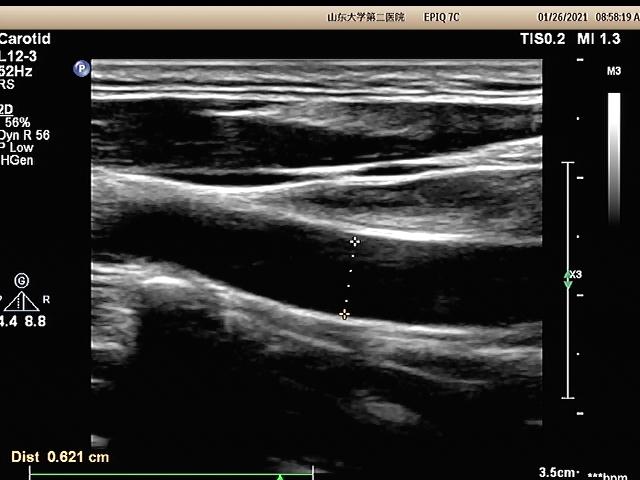

Supplement: Supplementary file 1 [file Data_Sheet_1.ZIP › CONTROLdate2/103.1.JPG]

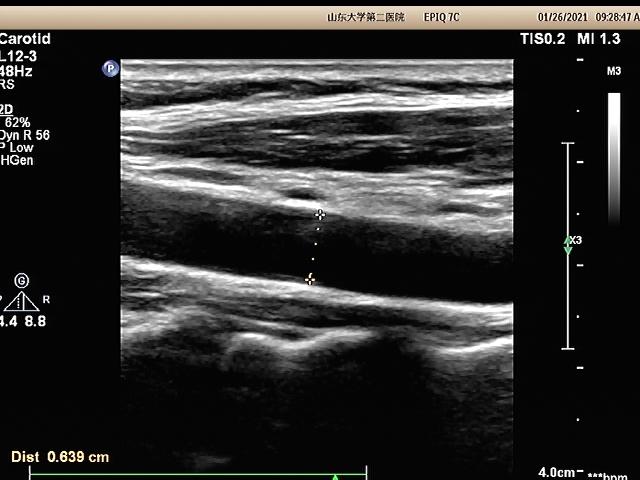

Supplement: Supplementary file 1 [file Data_Sheet_1.ZIP › CONTROLdate2/104.1.JPG]

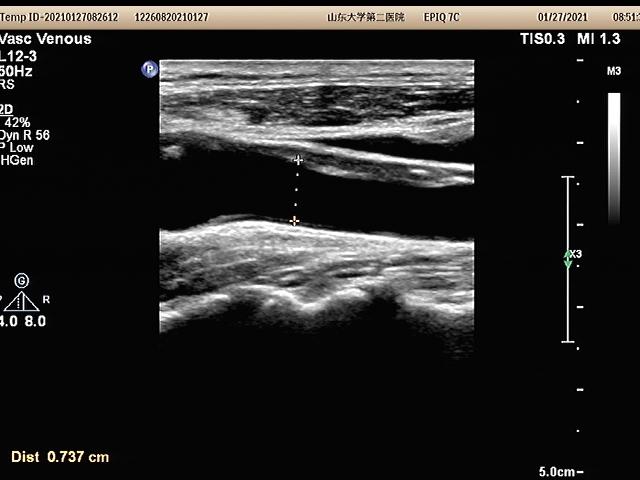

Supplement: Supplementary file 1 [file Data_Sheet_1.ZIP › CONTROLdate2/105.1.JPG]

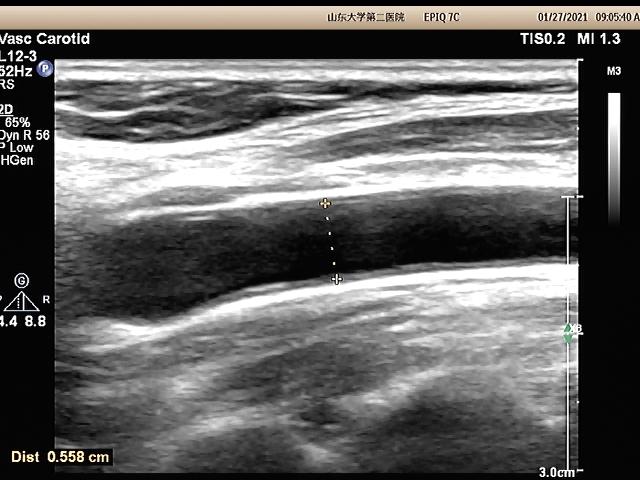

Supplement: Supplementary file 1 [file Data_Sheet_1.ZIP › CONTROLdate2/106.1.JPG]

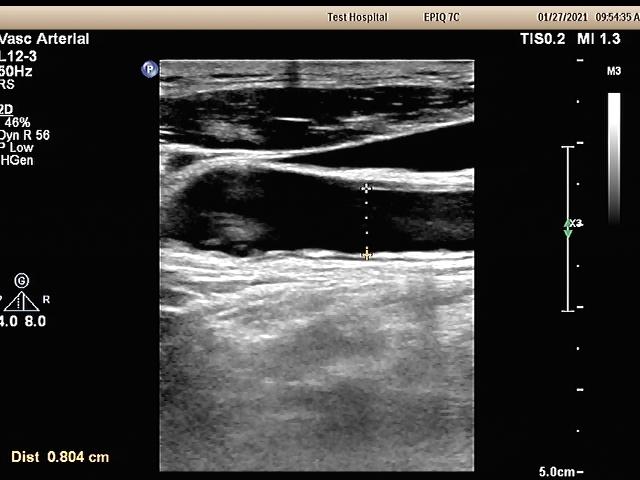

Supplement: Supplementary file 1 [file Data_Sheet_1.ZIP › CONTROLdate2/107.1.JPG]

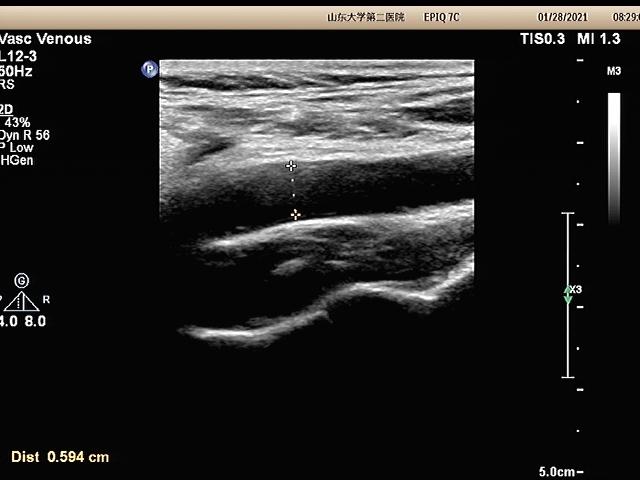

Supplement: Supplementary file 1 [file Data_Sheet_1.ZIP › CONTROLdate2/108.1.JPG]

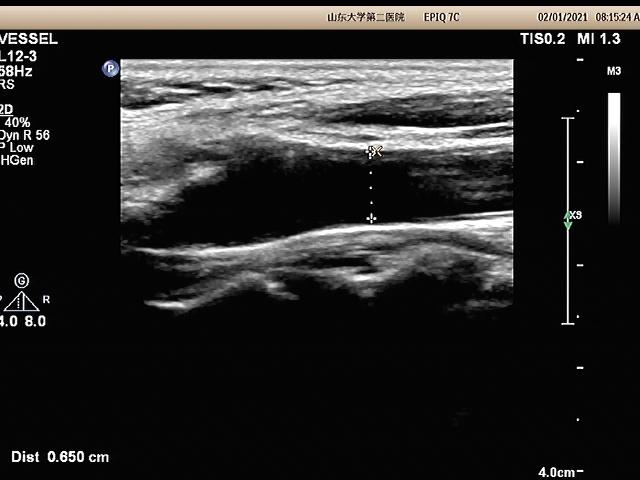

Supplement: Supplementary file 1 [file Data_Sheet_1.ZIP › CONTROLdate2/109.1.JPG]

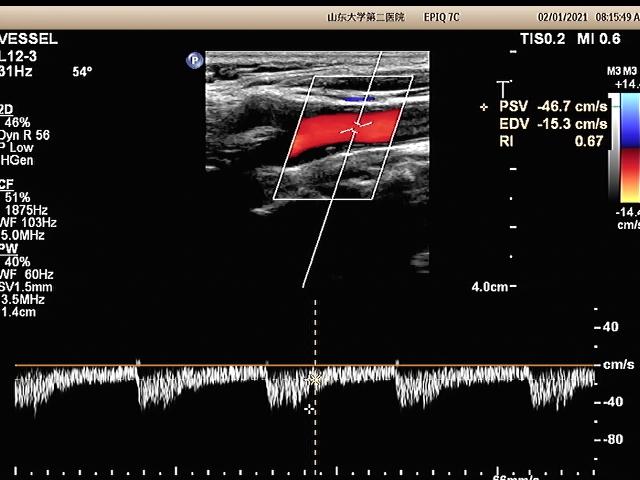

Supplement: Supplementary file 1 [file Data_Sheet_1.ZIP › CONTROLdate2/109.2.JPG]

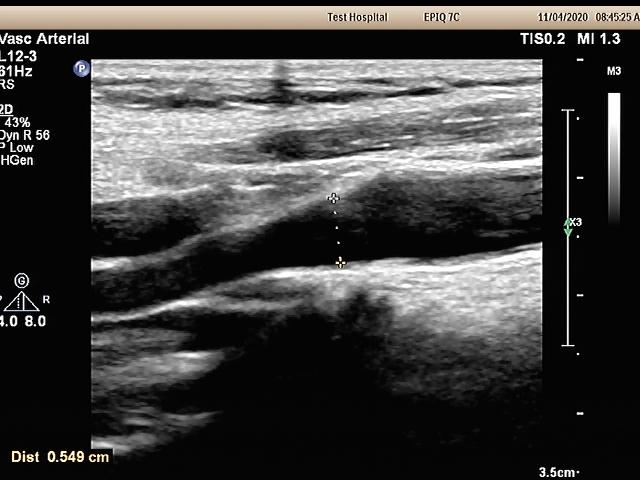

Supplement: Supplementary file 1 [file Data_Sheet_1.ZIP › CONTROLdate2/11.1.JPG]

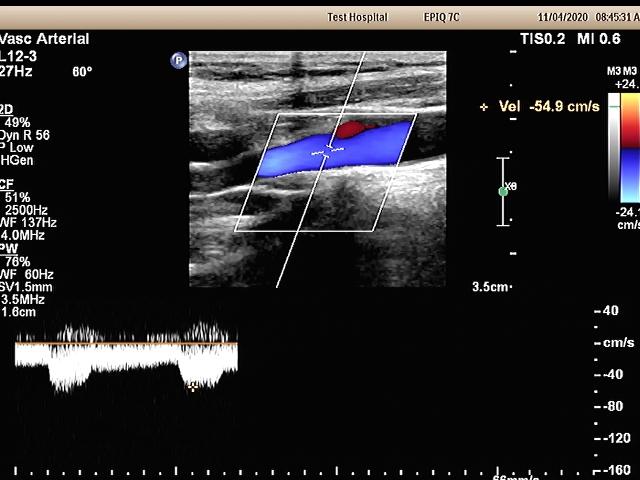

Supplement: Supplementary file 1 [file Data_Sheet_1.ZIP › CONTROLdate2/11.2.JPG]

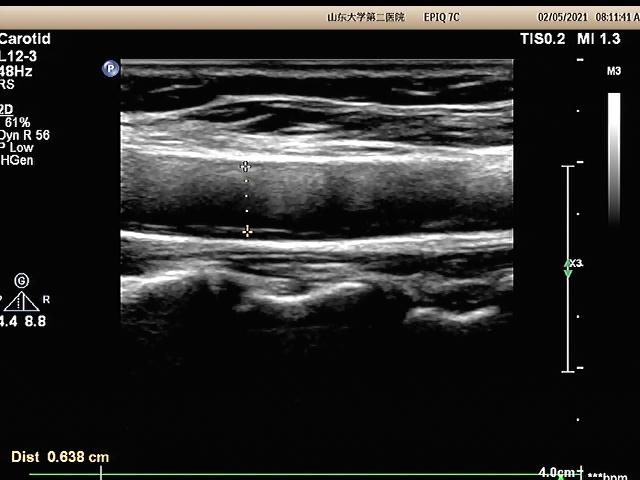

Supplement: Supplementary file 1 [file Data_Sheet_1.ZIP › CONTROLdate2/110.1.JPG]

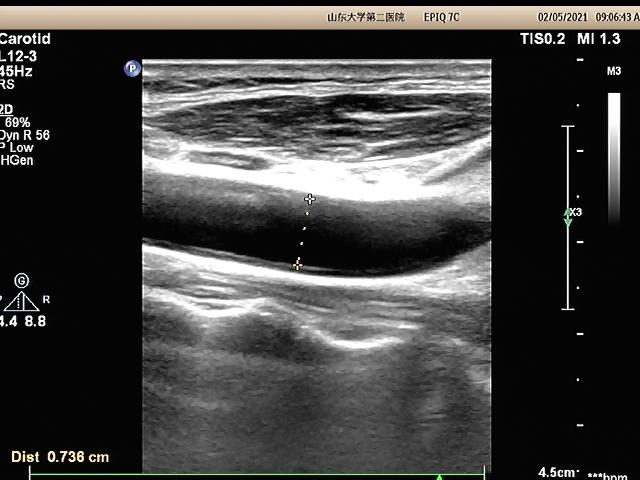

Supplement: Supplementary file 1 [file Data_Sheet_1.ZIP › CONTROLdate2/111.1.JPG]

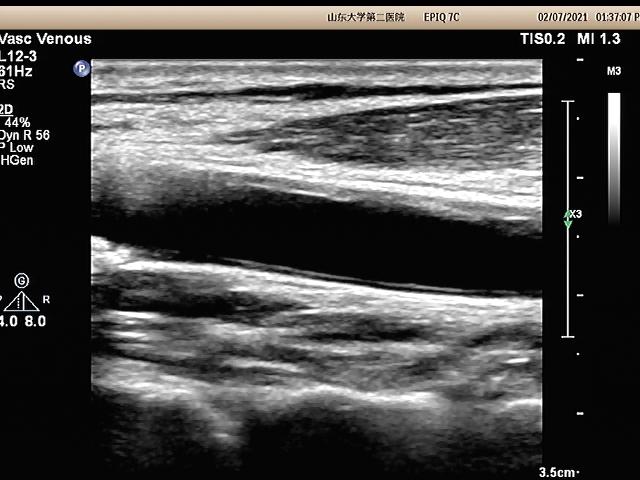

Supplement: Supplementary file 1 [file Data_Sheet_1.ZIP › CONTROLdate2/112.1.JPG]

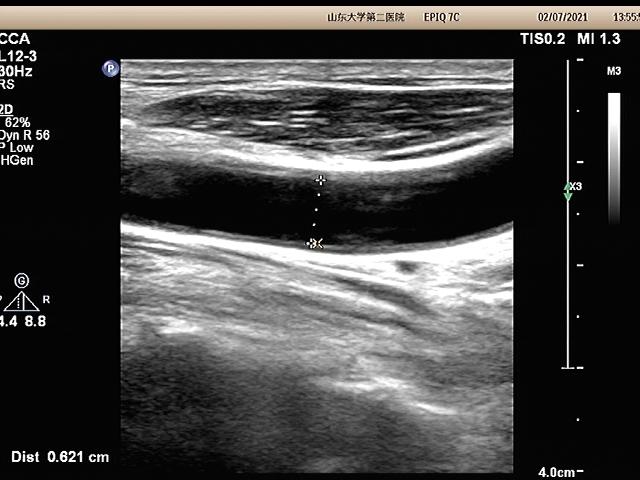

Supplement: Supplementary file 1 [file Data_Sheet_1.ZIP › CONTROLdate2/113.1.JPG]

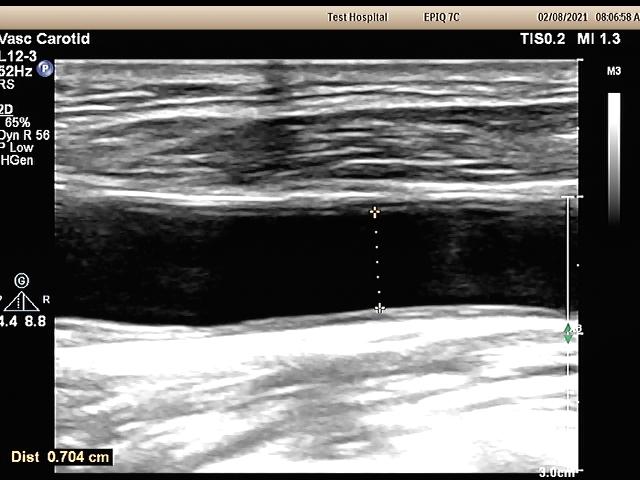

Supplement: Supplementary file 1 [file Data_Sheet_1.ZIP › CONTROLdate2/114.1.JPG]

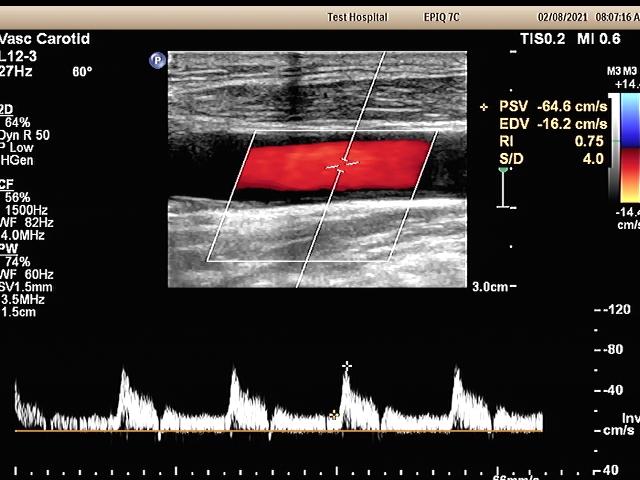

Supplement: Supplementary file 1 [file Data_Sheet_1.ZIP › CONTROLdate2/114.2.JPG]

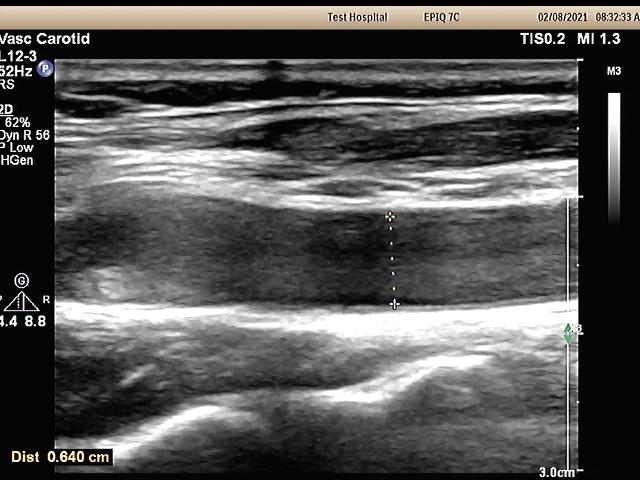

Supplement: Supplementary file 1 [file Data_Sheet_1.ZIP › CONTROLdate2/115.1.JPG]

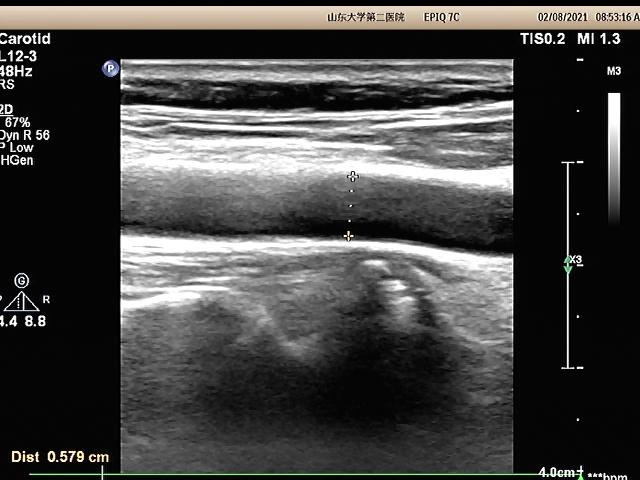

Supplement: Supplementary file 1 [file Data_Sheet_1.ZIP › CONTROLdate2/116.1.JPG]

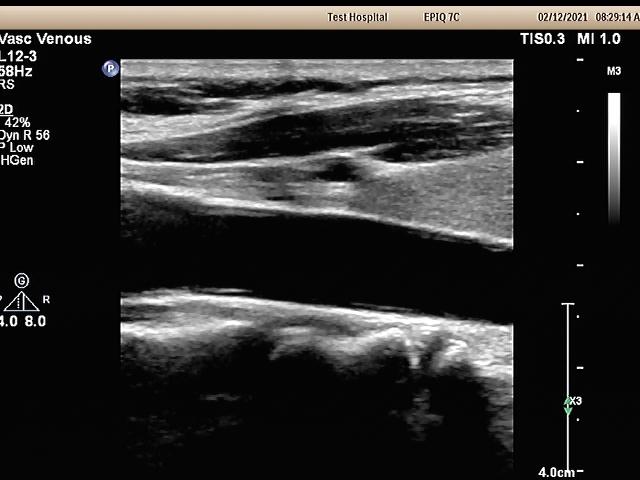

Supplement: Supplementary file 1 [file Data_Sheet_1.ZIP › CONTROLdate2/117.1.JPG]

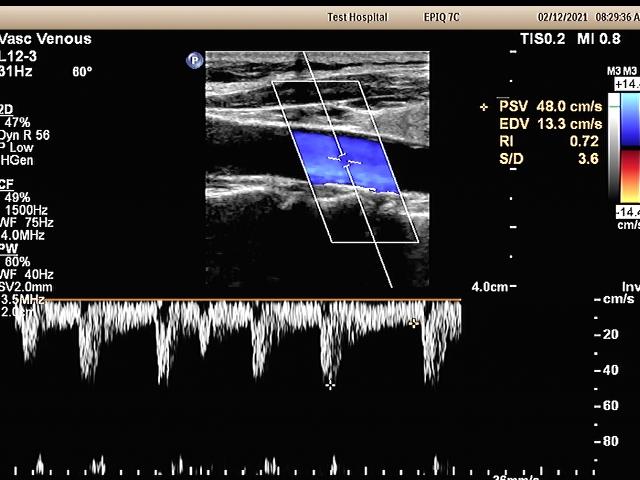

Supplement: Supplementary file 1 [file Data_Sheet_1.ZIP › CONTROLdate2/117.2.JPG]

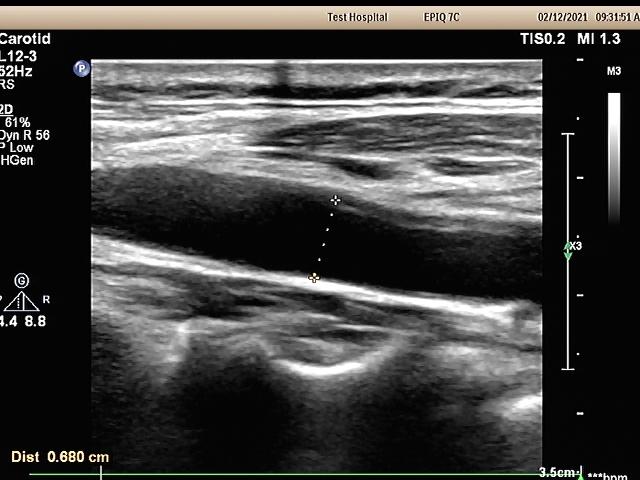

Supplement: Supplementary file 1 [file Data_Sheet_1.ZIP › CONTROLdate2/118.1.JPG]

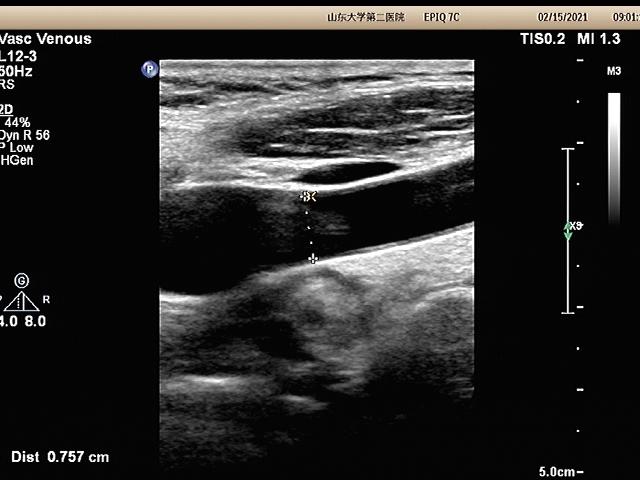

Supplement: Supplementary file 1 [file Data_Sheet_1.ZIP › CONTROLdate2/119.1.JPG]

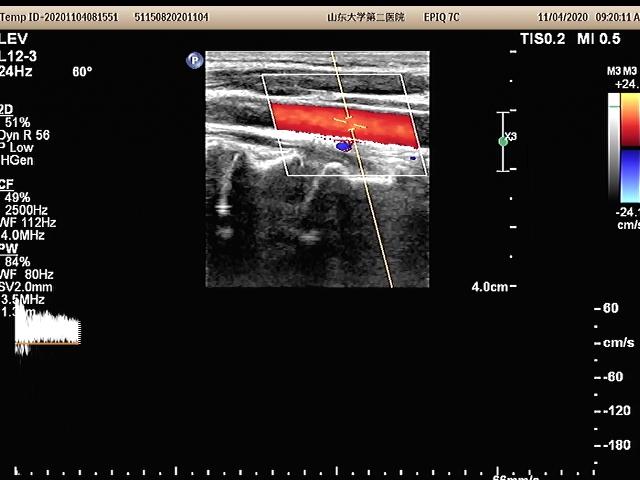

Supplement: Supplementary file 1 [file Data_Sheet_1.ZIP › CONTROLdate2/12.1.JPG]

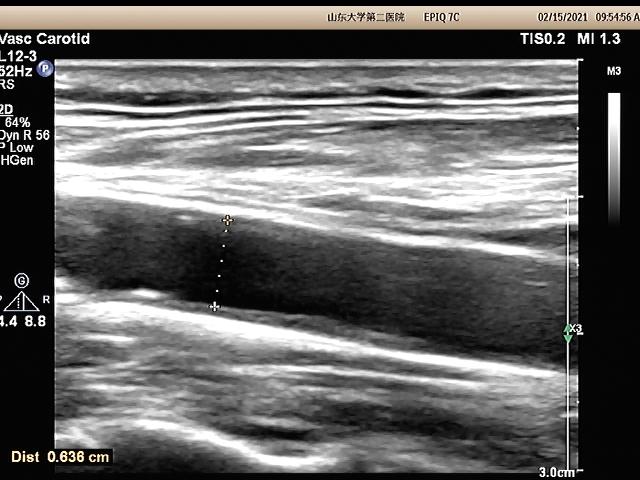

Supplement: Supplementary file 1 [file Data_Sheet_1.ZIP › CONTROLdate2/120.1.JPG]

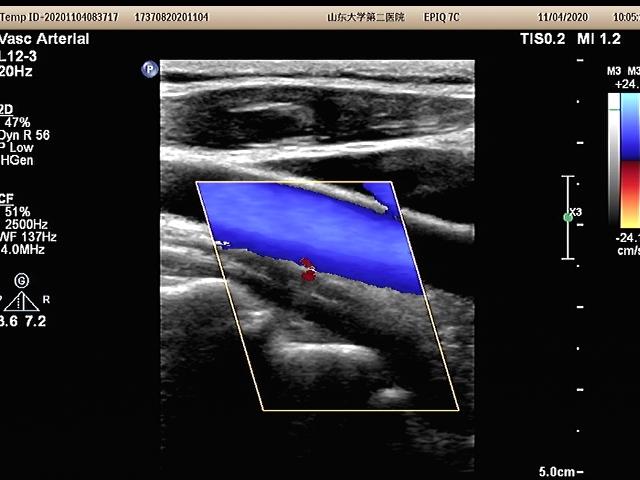

Supplement: Supplementary file 1 [file Data_Sheet_1.ZIP › CONTROLdate2/13.1.JPG]

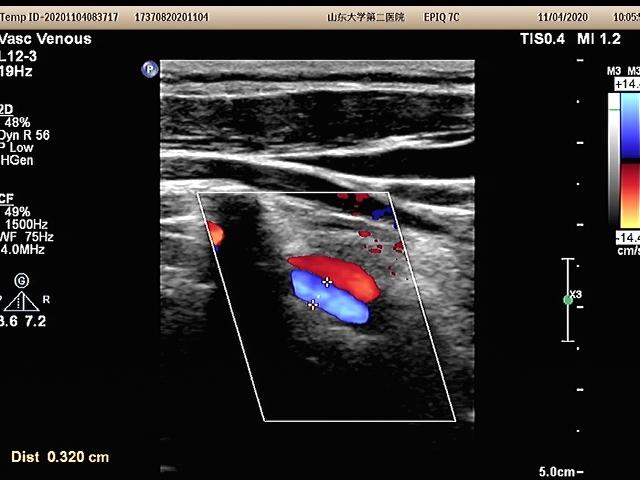

Supplement: Supplementary file 1 [file Data_Sheet_1.ZIP › CONTROLdate2/13.2.JPG]

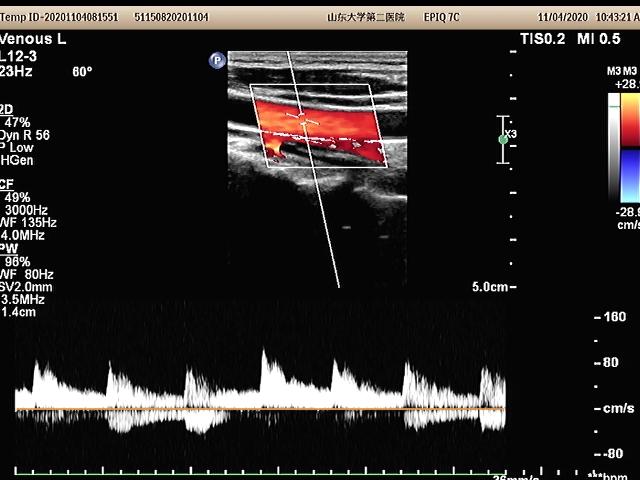

Supplement: Supplementary file 1 [file Data_Sheet_1.ZIP › CONTROLdate2/14.1.JPG]

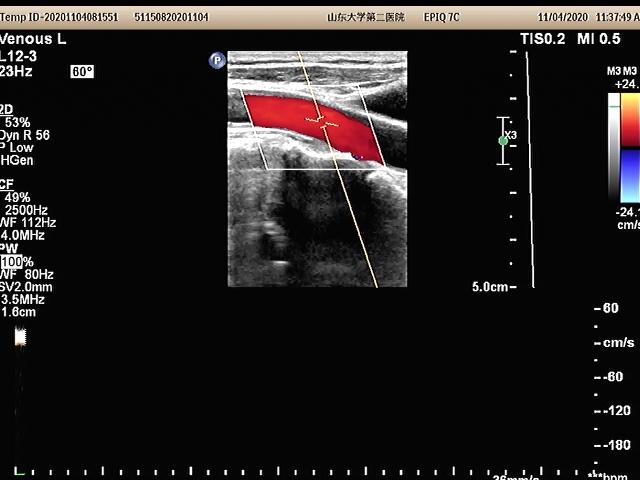

Supplement: Supplementary file 1 [file Data_Sheet_1.ZIP › CONTROLdate2/15.1.JPG]

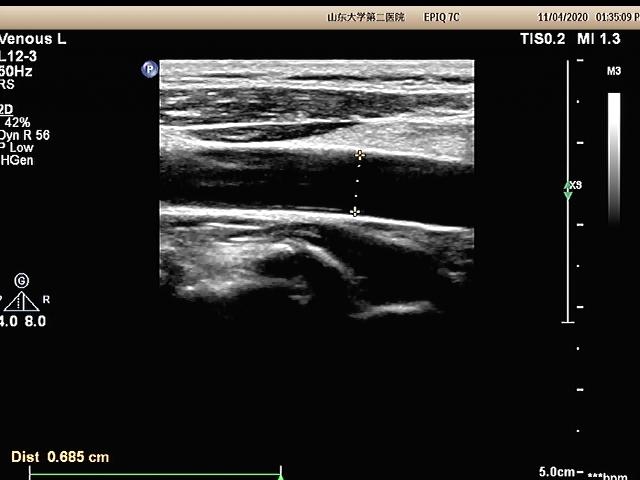

Supplement: Supplementary file 1 [file Data_Sheet_1.ZIP › CONTROLdate2/16.1.JPG]

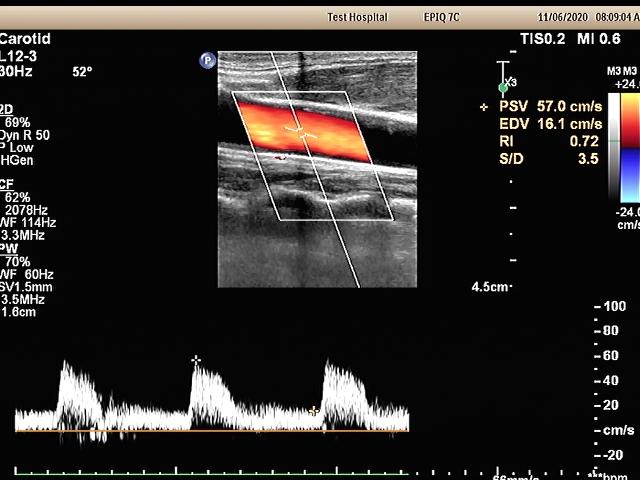

Supplement: Supplementary file 1 [file Data_Sheet_1.ZIP › CONTROLdate2/17.1.JPG]

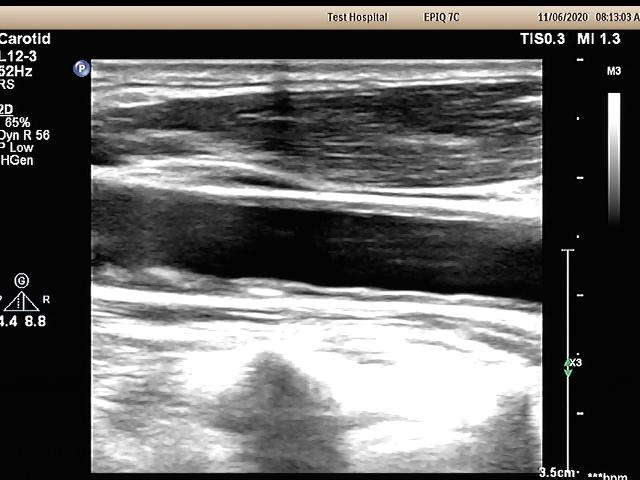

Supplement: Supplementary file 1 [file Data_Sheet_1.ZIP › CONTROLdate2/17.2.JPG]

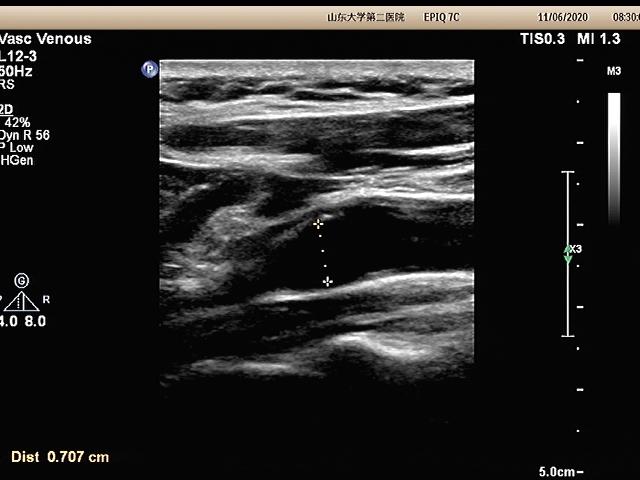

Supplement: Supplementary file 1 [file Data_Sheet_1.ZIP › CONTROLdate2/18.1.JPG]

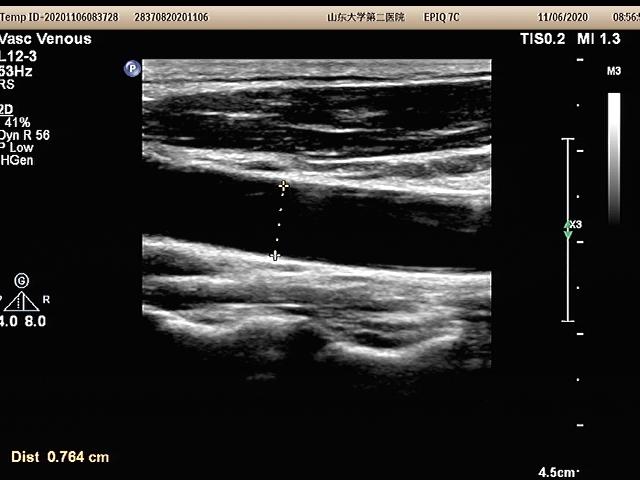

Supplement: Supplementary file 1 [file Data_Sheet_1.ZIP › CONTROLdate2/19.1.JPG]

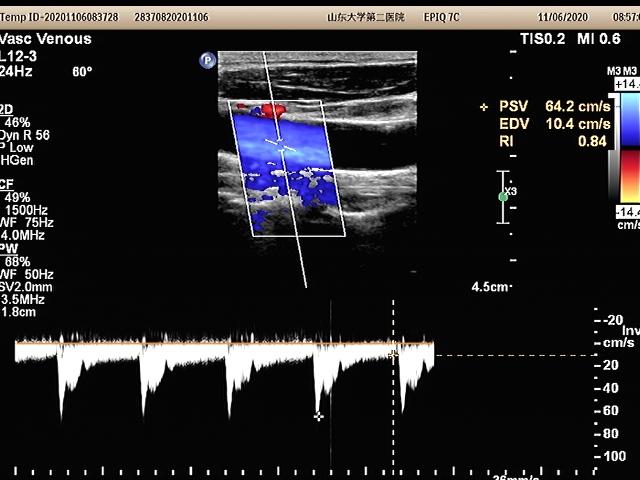

Supplement: Supplementary file 1 [file Data_Sheet_1.ZIP › CONTROLdate2/19.2.JPG]

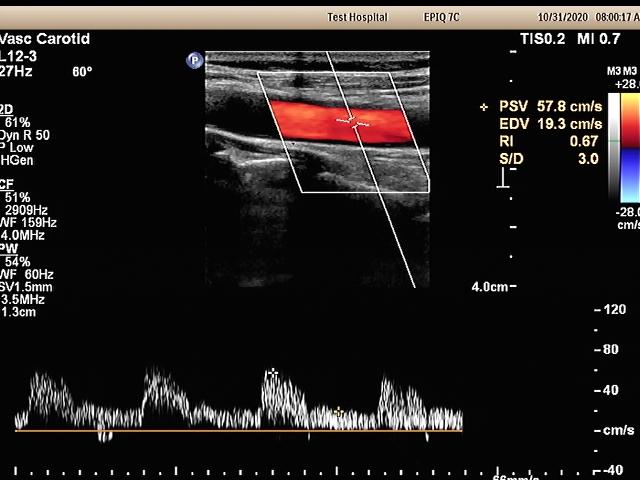

Supplement: Supplementary file 1 [file Data_Sheet_1.ZIP › CONTROLdate2/2.1.JPG]

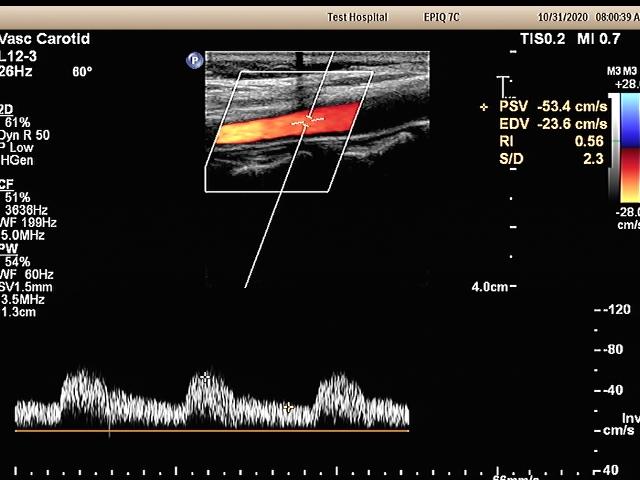

Supplement: Supplementary file 1 [file Data_Sheet_1.ZIP › CONTROLdate2/2.2.JPG]

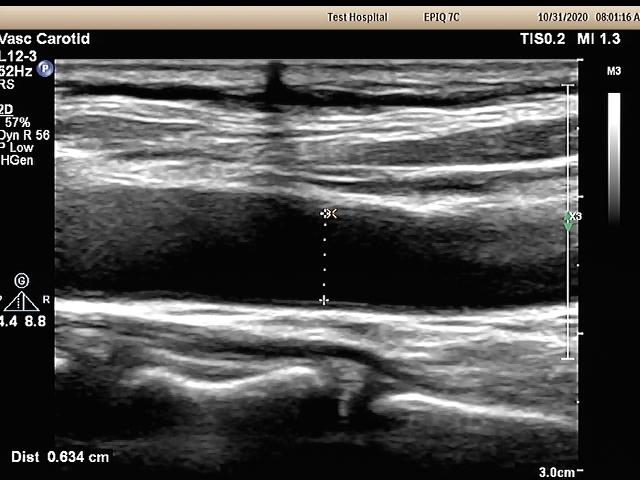

Supplement: Supplementary file 1 [file Data_Sheet_1.ZIP › CONTROLdate2/2.3.JPG]

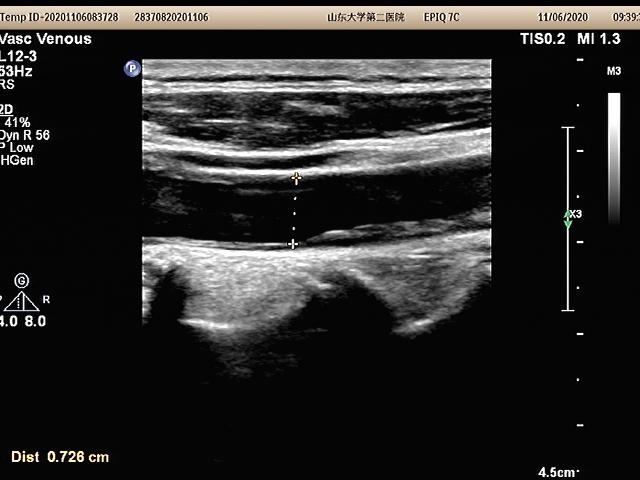

Supplement: Supplementary file 1 [file Data_Sheet_1.ZIP › CONTROLdate2/20.1.JPG]

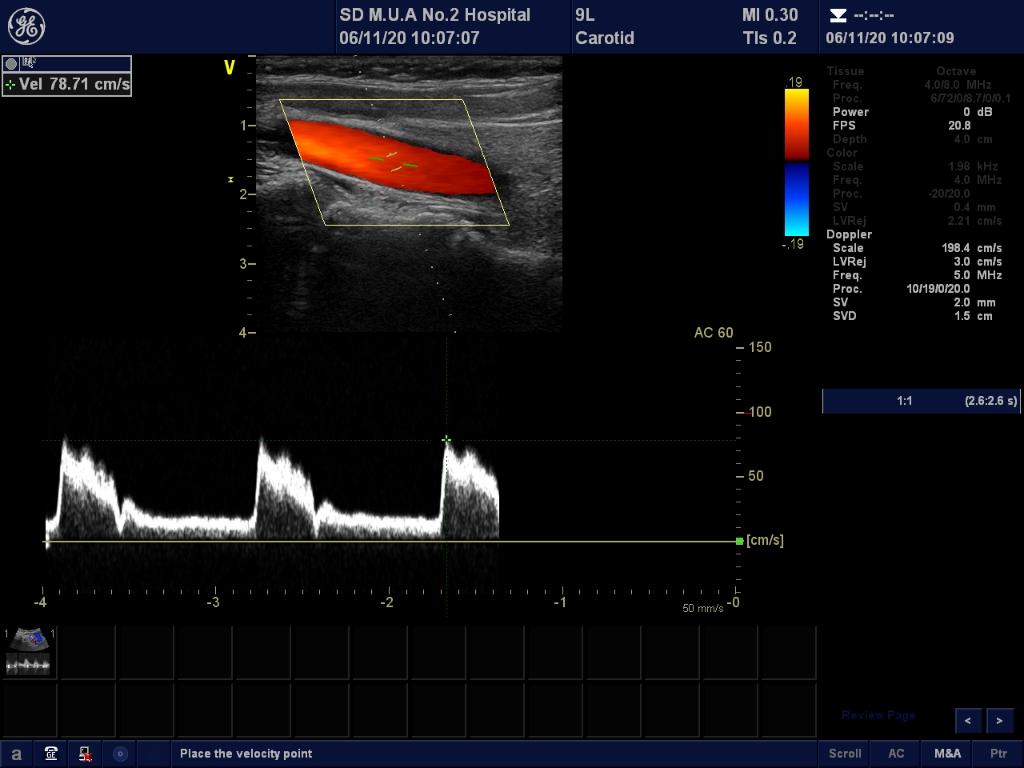

Supplement: Supplementary file 1 [file Data_Sheet_1.ZIP › CONTROLdate2/21.1.JPG]

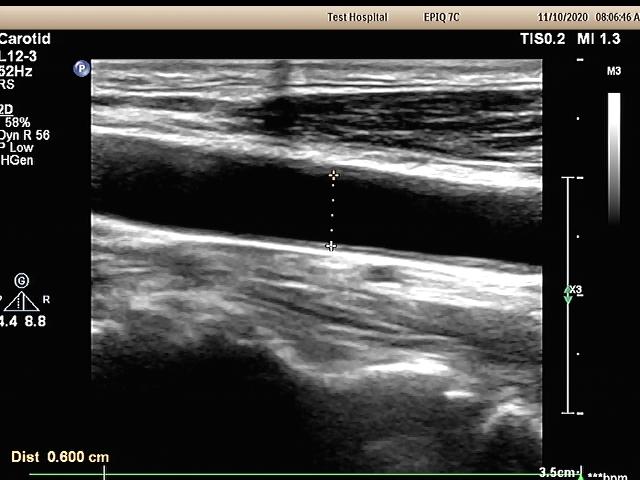

Supplement: Supplementary file 1 [file Data_Sheet_1.ZIP › CONTROLdate2/22.1.JPG]

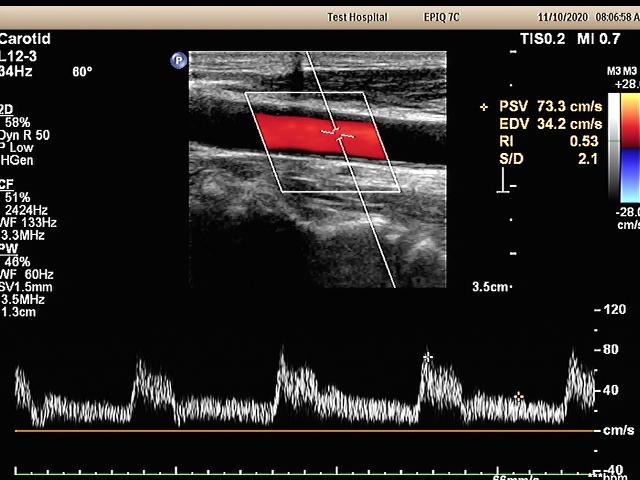

Supplement: Supplementary file 1 [file Data_Sheet_1.ZIP › CONTROLdate2/22.2.JPG]

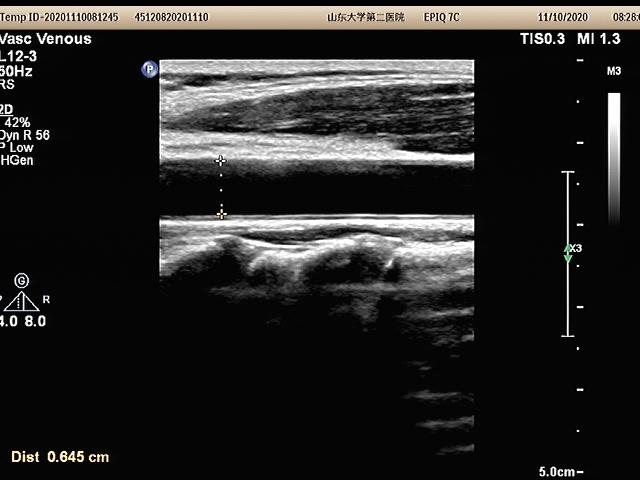

Supplement: Supplementary file 1 [file Data_Sheet_1.ZIP › CONTROLdate2/23.1.JPG]

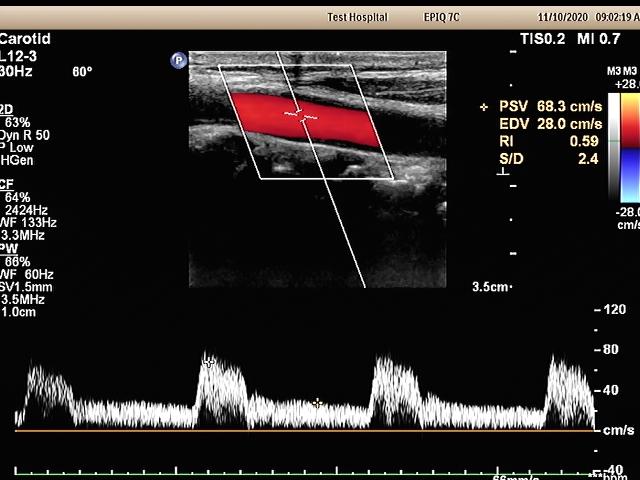

Supplement: Supplementary file 1 [file Data_Sheet_1.ZIP › CONTROLdate2/24.1.JPG]

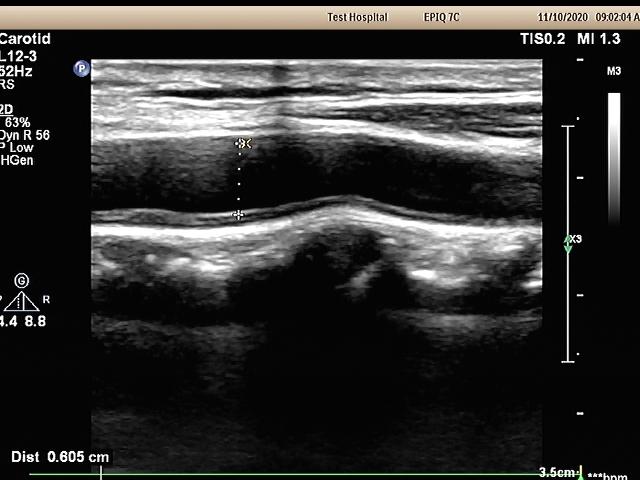

Supplement: Supplementary file 1 [file Data_Sheet_1.ZIP › CONTROLdate2/24.2.JPG]

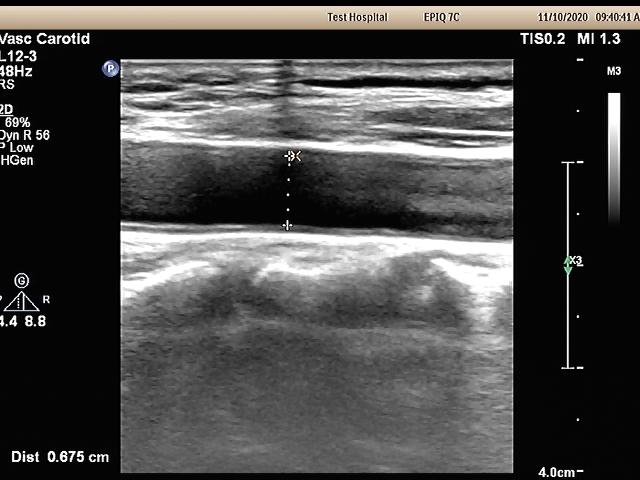

Supplement: Supplementary file 1 [file Data_Sheet_1.ZIP › CONTROLdate2/25.1.JPG]

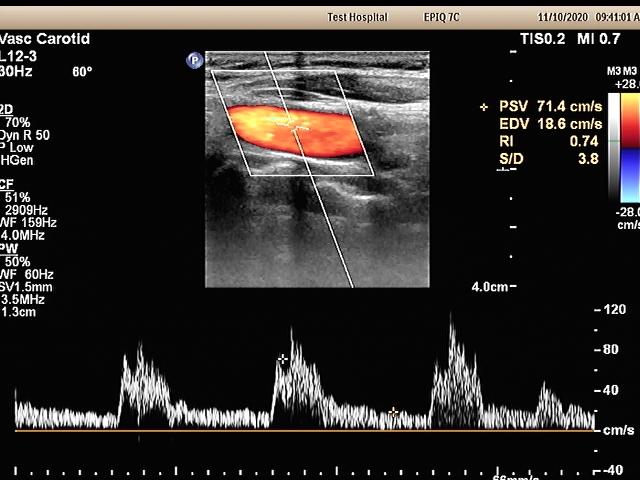

Supplement: Supplementary file 1 [file Data_Sheet_1.ZIP › CONTROLdate2/25.2.JPG]

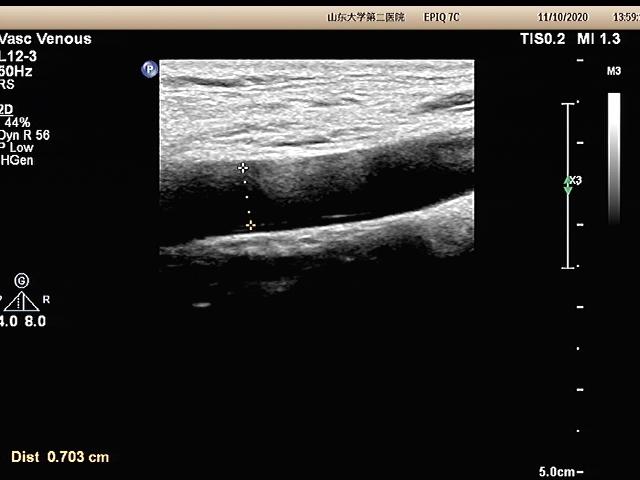

Supplement: Supplementary file 1 [file Data_Sheet_1.ZIP › CONTROLdate2/26.2.JPG]

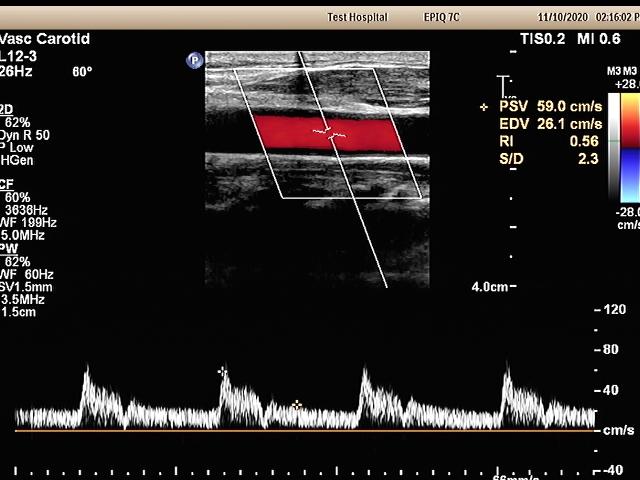

Supplement: Supplementary file 1 [file Data_Sheet_1.ZIP › CONTROLdate2/27.1.JPG]

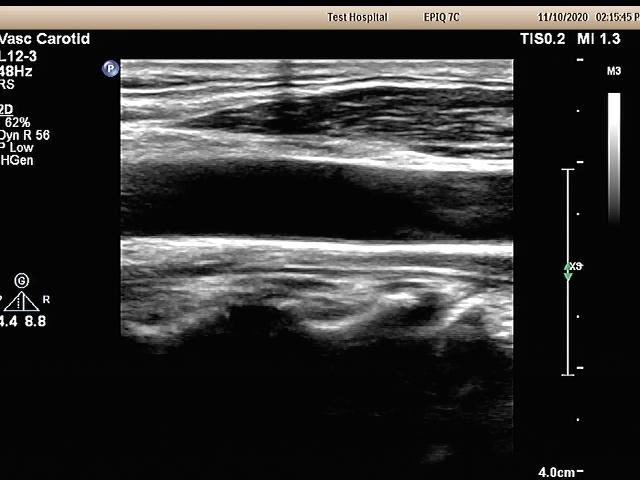

Supplement: Supplementary file 1 [file Data_Sheet_1.ZIP › CONTROLdate2/27.2.JPG]

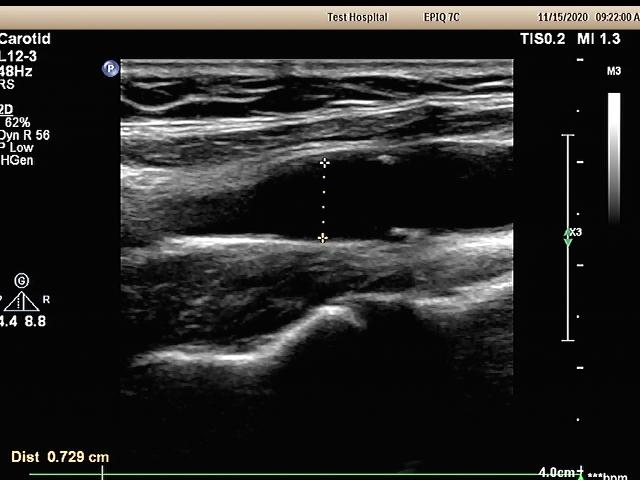

Supplement: Supplementary file 1 [file Data_Sheet_1.ZIP › CONTROLdate2/28.1.JPG]

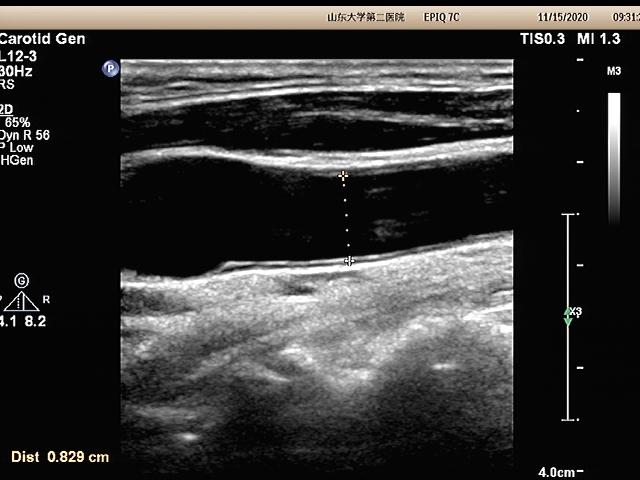

Supplement: Supplementary file 1 [file Data_Sheet_1.ZIP › CONTROLdate2/29.1.JPG]

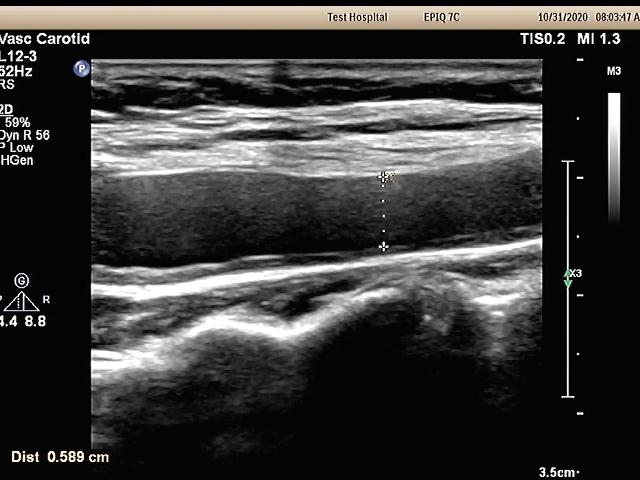

Supplement: Supplementary file 1 [file Data_Sheet_1.ZIP › CONTROLdate2/3.1.JPG]

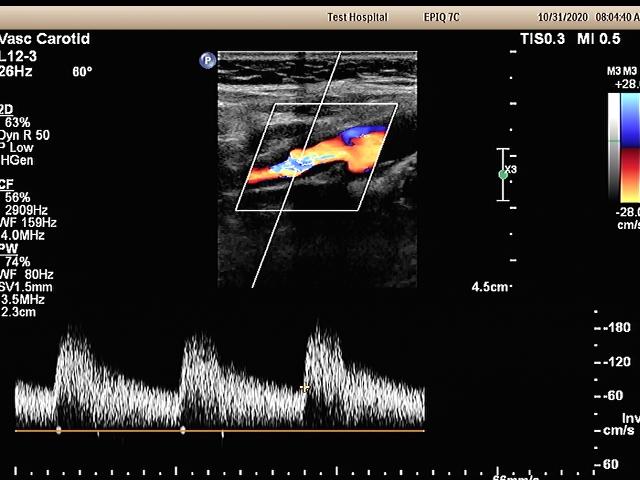

Supplement: Supplementary file 1 [file Data_Sheet_1.ZIP › CONTROLdate2/3.2.JPG]

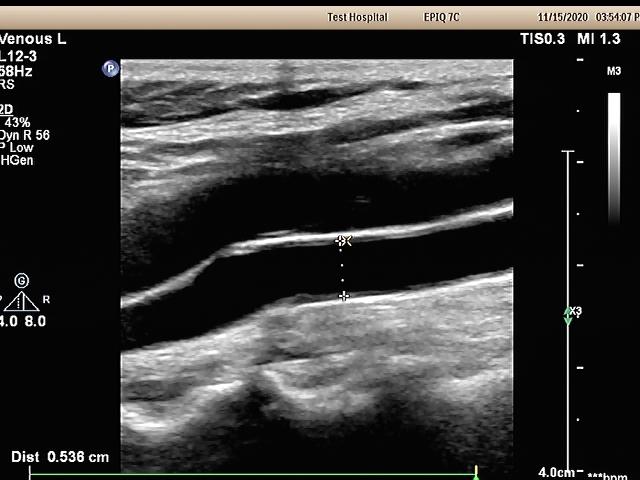

Supplement: Supplementary file 1 [file Data_Sheet_1.ZIP › CONTROLdate2/30.1.JPG]

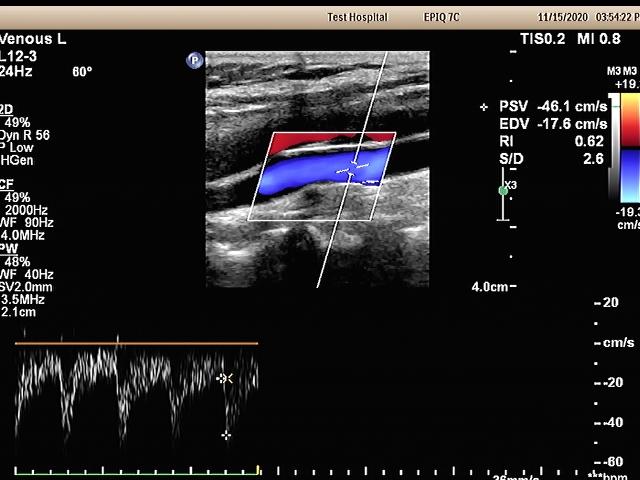

Supplement: Supplementary file 1 [file Data_Sheet_1.ZIP › CONTROLdate2/30.2.JPG]

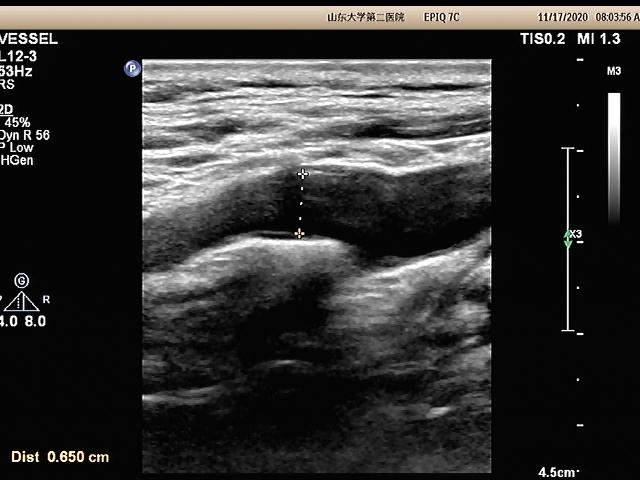

Supplement: Supplementary file 1 [file Data_Sheet_1.ZIP › CONTROLdate2/31.1.JPG]

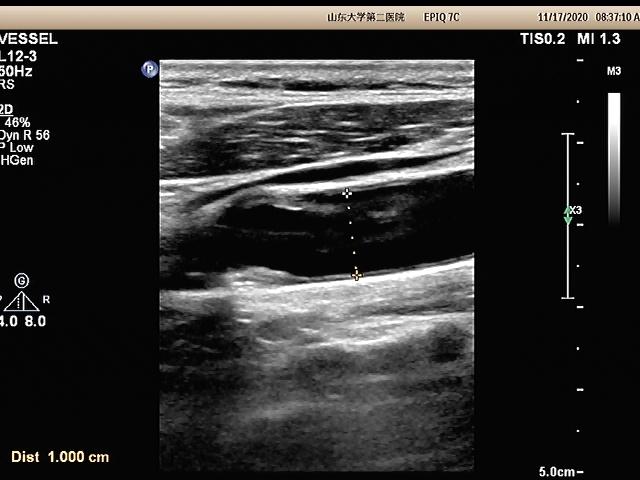

Supplement: Supplementary file 1 [file Data_Sheet_1.ZIP › CONTROLdate2/32.1.JPG]

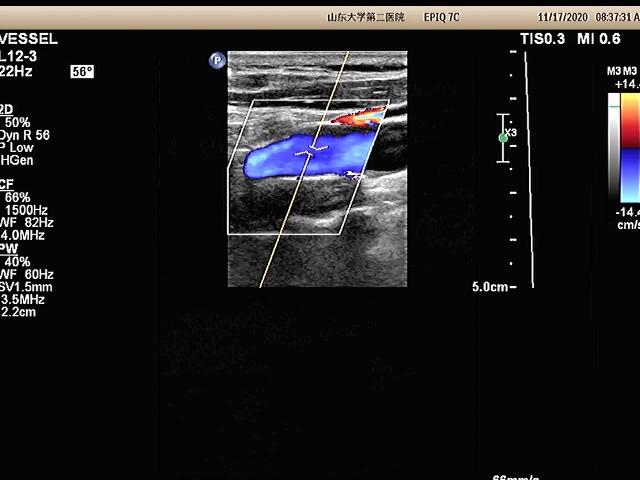

Supplement: Supplementary file 1 [file Data_Sheet_1.ZIP › CONTROLdate2/32.2.JPG]

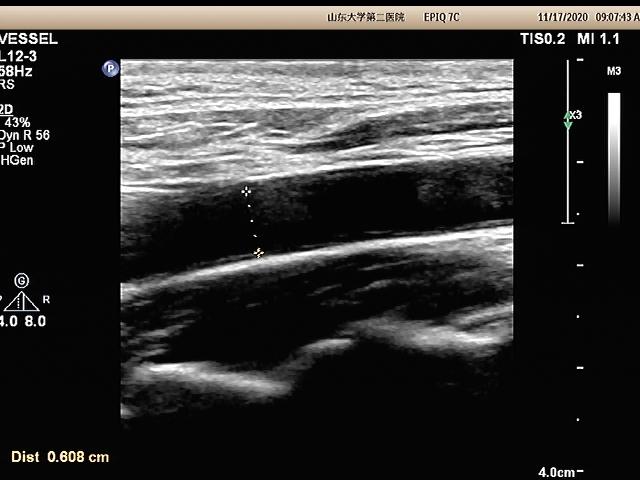

Supplement: Supplementary file 1 [file Data_Sheet_1.ZIP › CONTROLdate2/33.1.JPG]

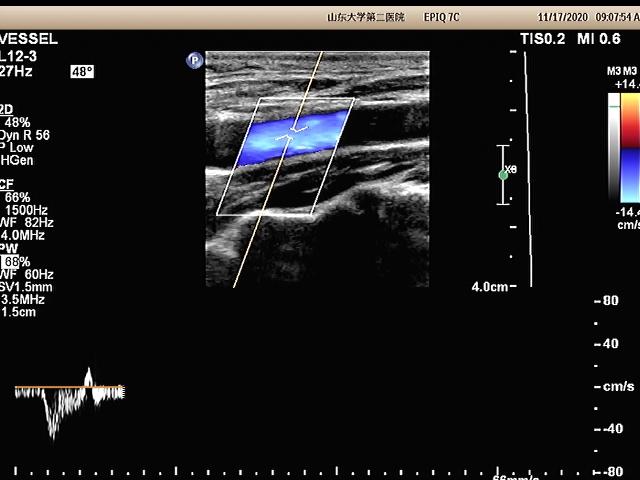

Supplement: Supplementary file 1 [file Data_Sheet_1.ZIP › CONTROLdate2/33.2.JPG]

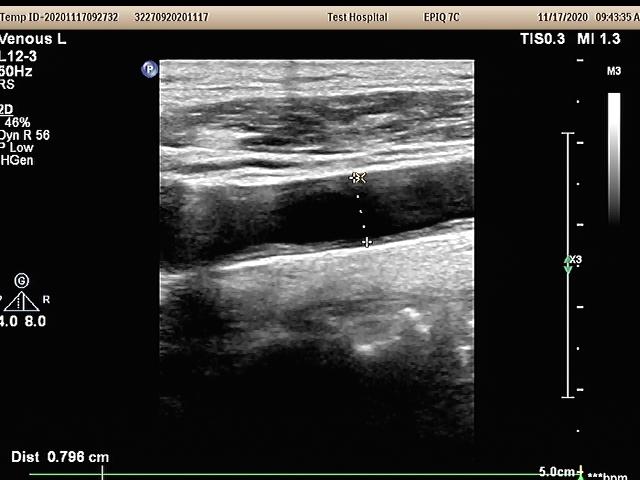

Supplement: Supplementary file 1 [file Data_Sheet_1.ZIP › CONTROLdate2/34.1.JPG]

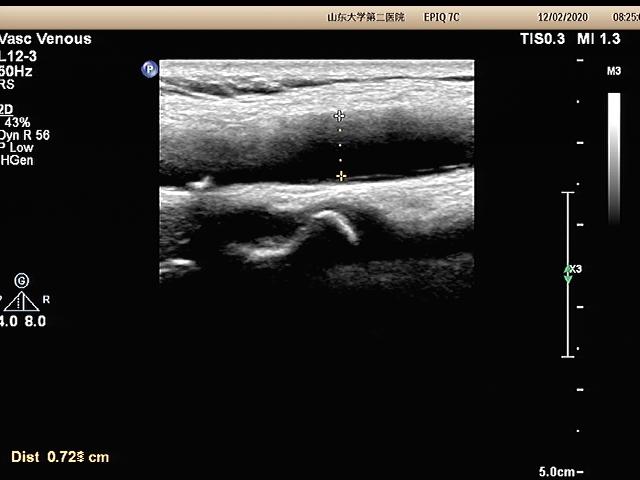

Supplement: Supplementary file 1 [file Data_Sheet_1.ZIP › CONTROLdate2/35.1.JPG]

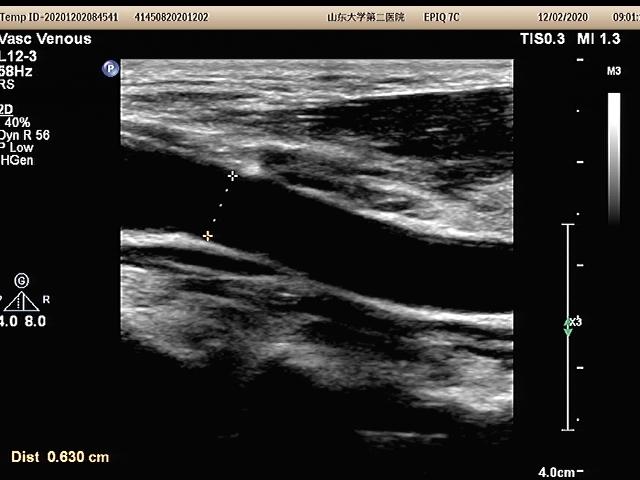

Supplement: Supplementary file 1 [file Data_Sheet_1.ZIP › CONTROLdate2/36.1.JPG]

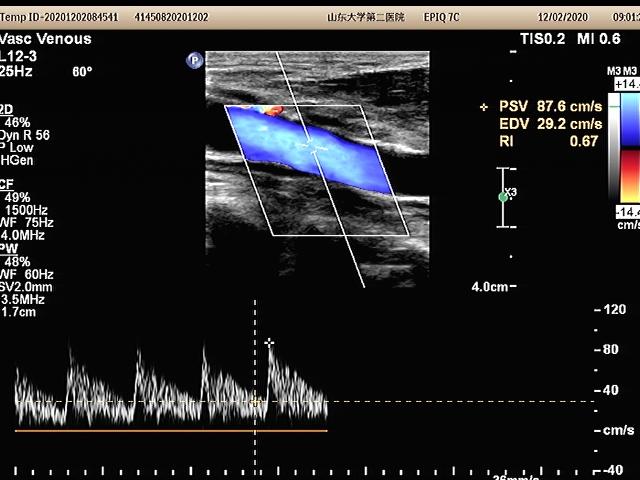

Supplement: Supplementary file 1 [file Data_Sheet_1.ZIP › CONTROLdate2/36.2.JPG]

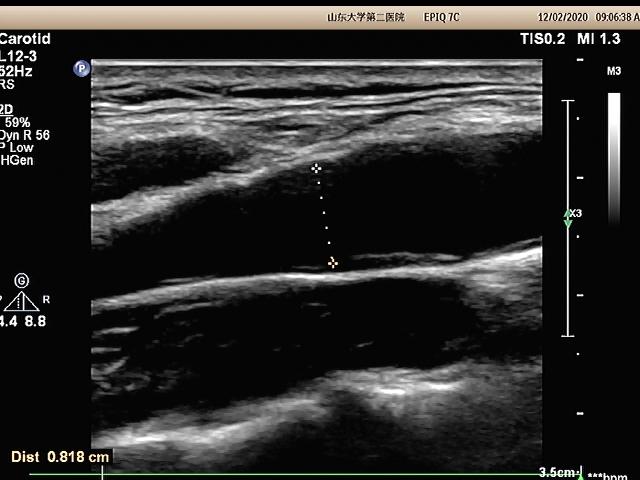

Supplement: Supplementary file 1 [file Data_Sheet_1.ZIP › CONTROLdate2/37.1.JPG]

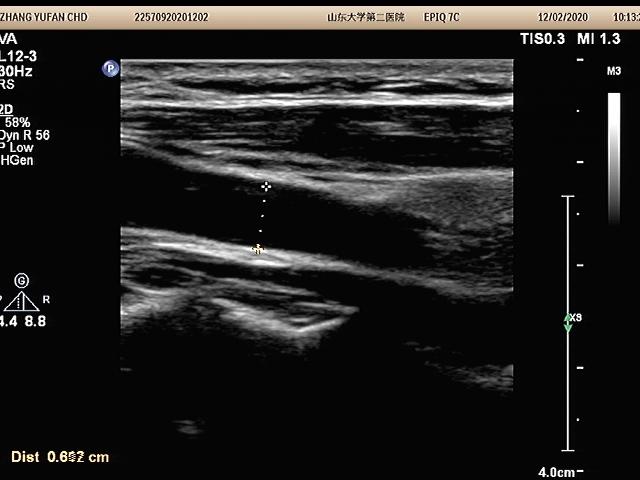

Supplement: Supplementary file 1 [file Data_Sheet_1.ZIP › CONTROLdate2/38.1.JPG]

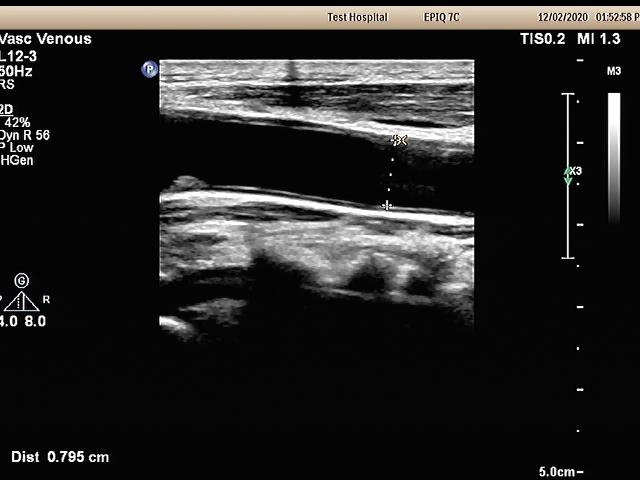

Supplement: Supplementary file 1 [file Data_Sheet_1.ZIP › CONTROLdate2/39.1.JPG]

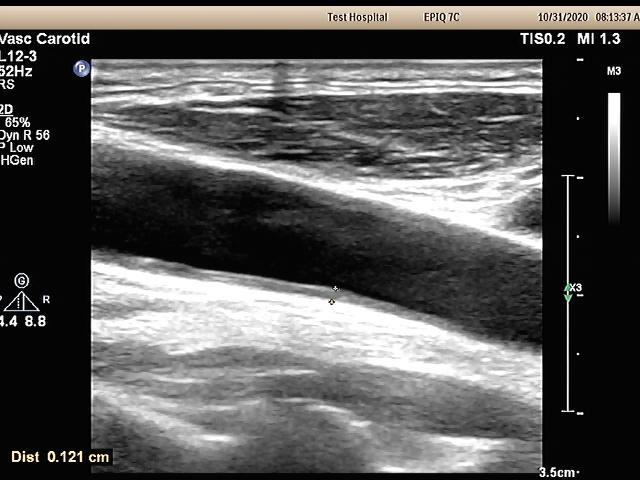

Supplement: Supplementary file 1 [file Data_Sheet_1.ZIP › CONTROLdate2/4.1.JPG]

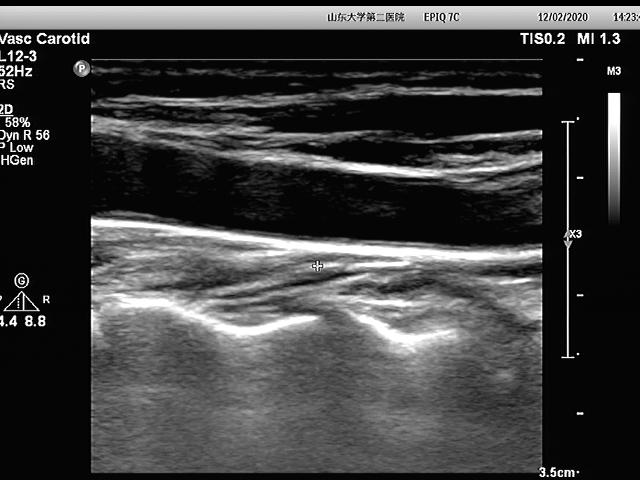

Supplement: Supplementary file 1 [file Data_Sheet_1.ZIP › CONTROLdate2/40.1.JPG]

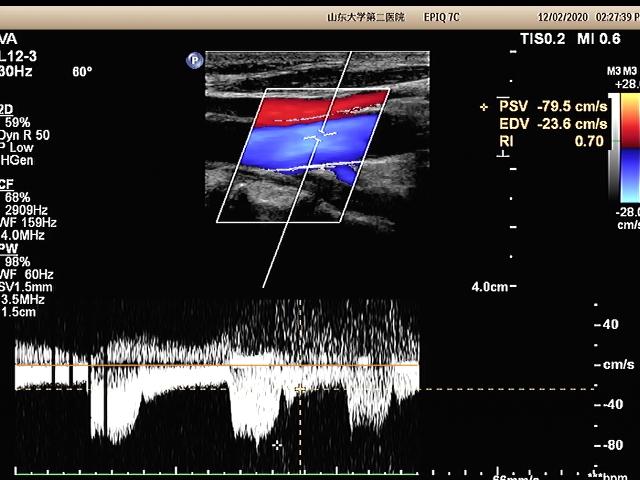

Supplement: Supplementary file 1 [file Data_Sheet_1.ZIP › CONTROLdate2/41.1.JPG]
