# Supplementary figures and images for: Enhanced external counterpulsation ameliorates endothelial dysfunction and elevates exercise tolerance in patients with coronary artery disease (part 1 of 2)
Source: Front Cardiovasc Med. 2022 Nov 29;9:997109. doi: 10.3389/fcvm.2022.997109 (PMC9744945; doi:10.3389/fcvm.2022.997109)

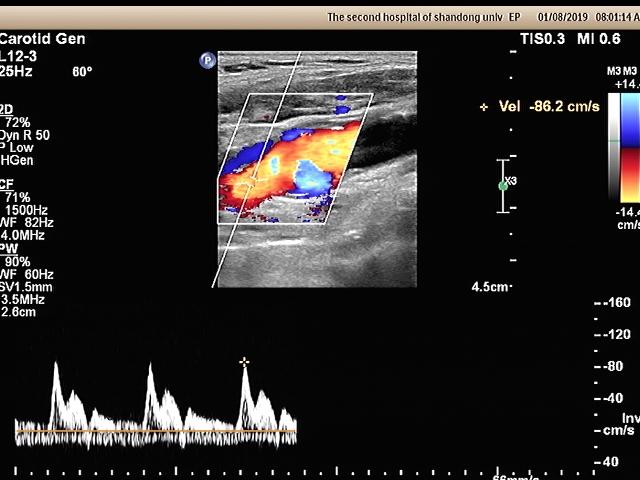

Supplement: Supplementary file 1 [file Data_Sheet_1.ZIP › CONTROLdate1/1.1.JPG]

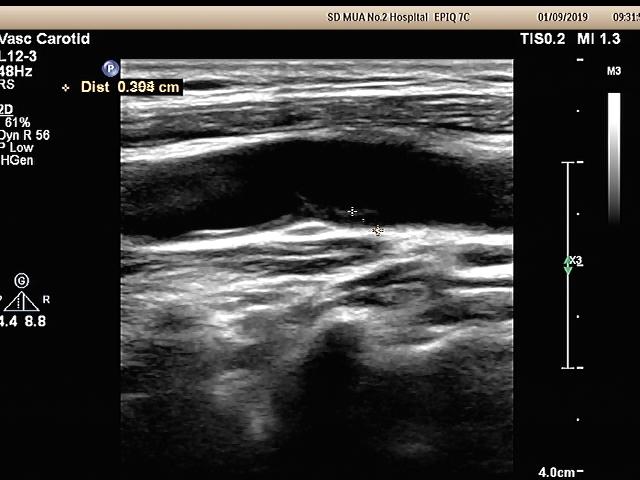

Supplement: Supplementary file 1 [file Data_Sheet_1.ZIP › CONTROLdate1/10.1.JPG]

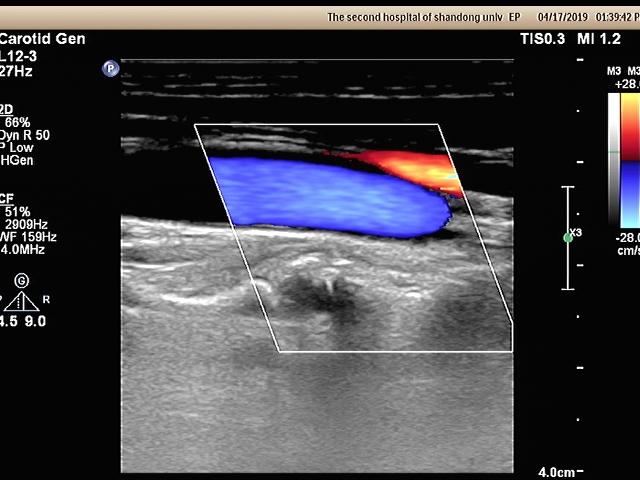

Supplement: Supplementary file 1 [file Data_Sheet_1.ZIP › CONTROLdate1/100.1.JPG]

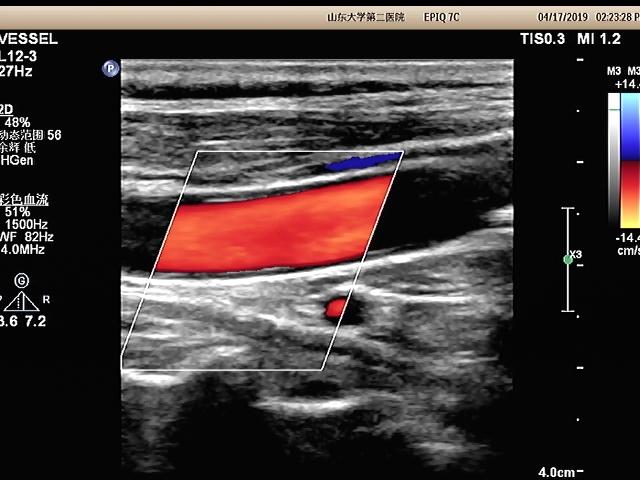

Supplement: Supplementary file 1 [file Data_Sheet_1.ZIP › CONTROLdate1/101.1.JPG]

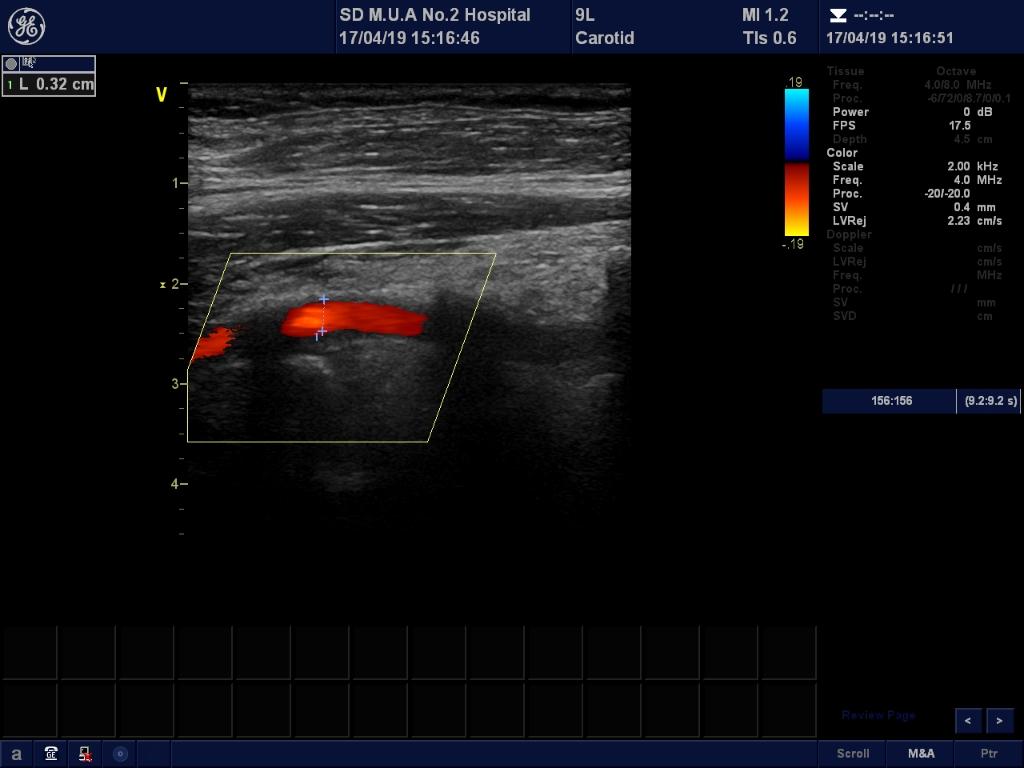

Supplement: Supplementary file 1 [file Data_Sheet_1.ZIP › CONTROLdate1/102.1.JPG]

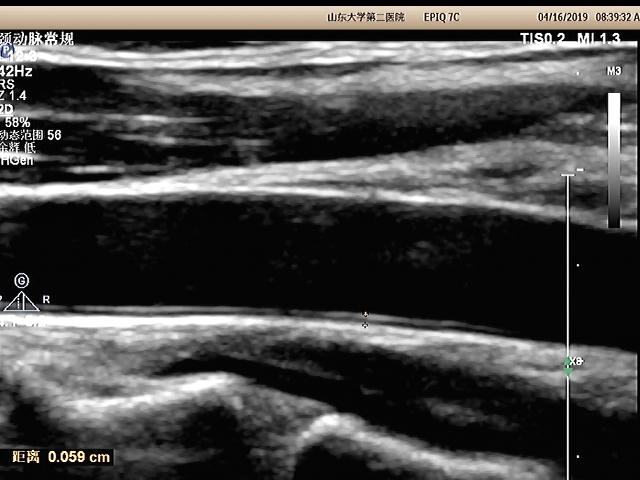

Supplement: Supplementary file 1 [file Data_Sheet_1.ZIP › CONTROLdate1/103.1.JPG]

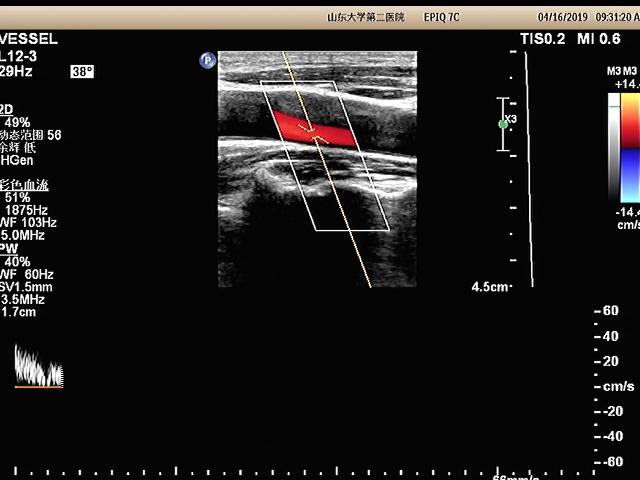

Supplement: Supplementary file 1 [file Data_Sheet_1.ZIP › CONTROLdate1/104.1.JPG]

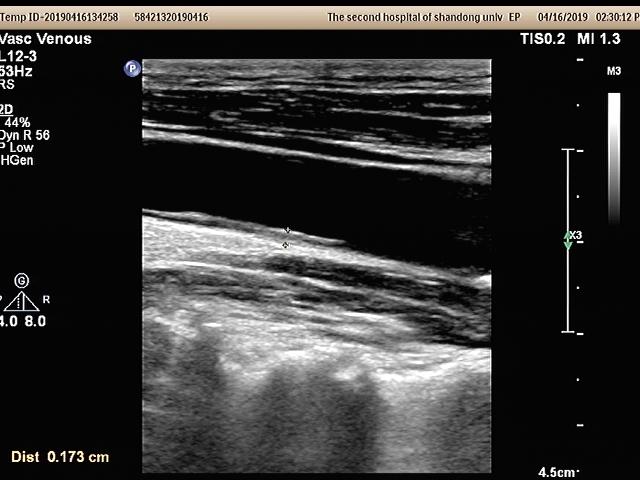

Supplement: Supplementary file 1 [file Data_Sheet_1.ZIP › CONTROLdate1/105.1.JPG]

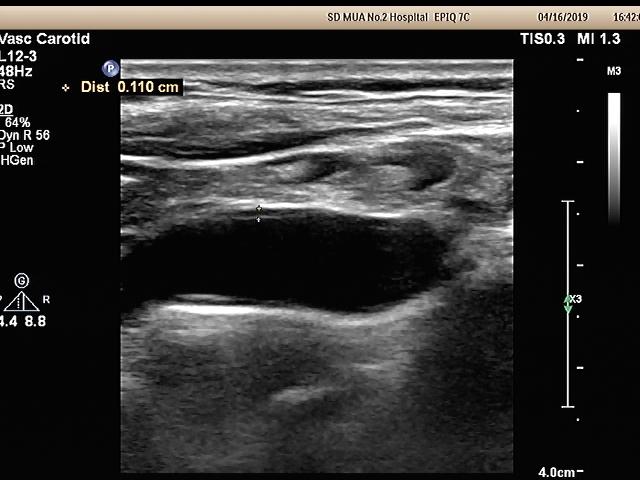

Supplement: Supplementary file 1 [file Data_Sheet_1.ZIP › CONTROLdate1/106.1.JPG]

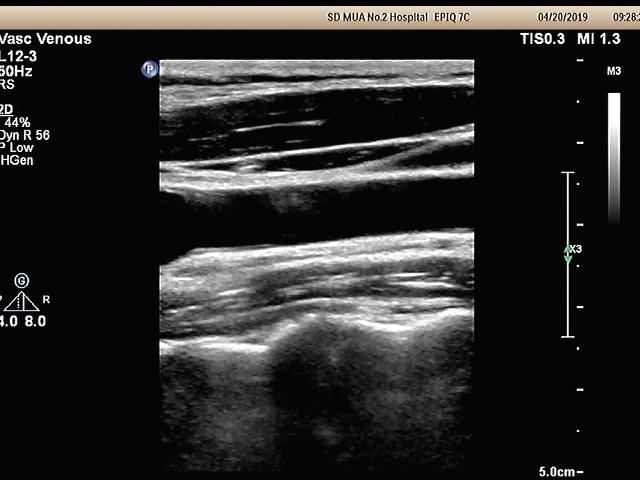

Supplement: Supplementary file 1 [file Data_Sheet_1.ZIP › CONTROLdate1/107.1.JPG]

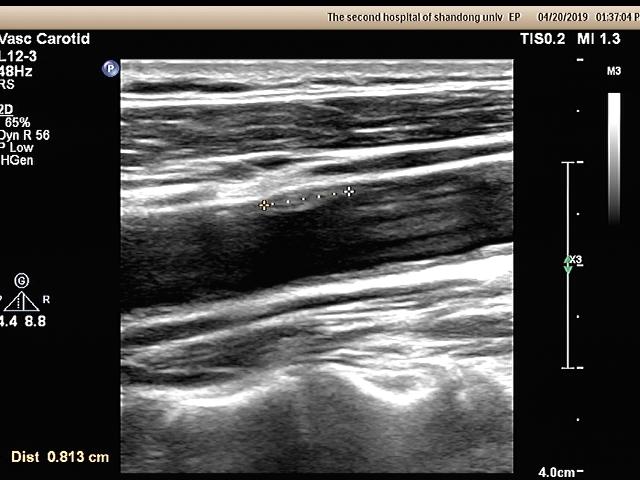

Supplement: Supplementary file 1 [file Data_Sheet_1.ZIP › CONTROLdate1/108.1.JPG]

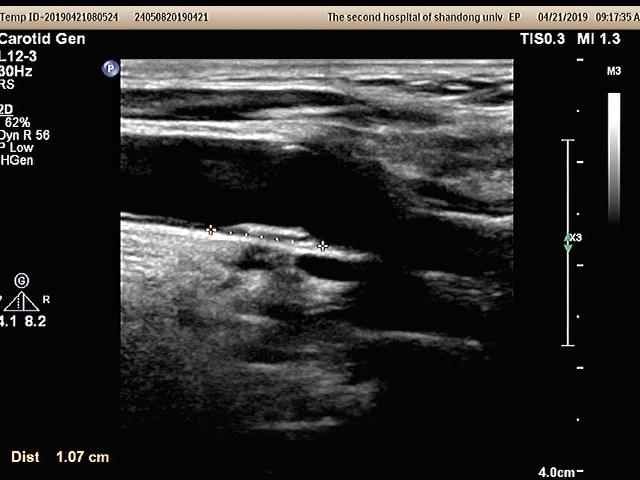

Supplement: Supplementary file 1 [file Data_Sheet_1.ZIP › CONTROLdate1/109.1.JPG]

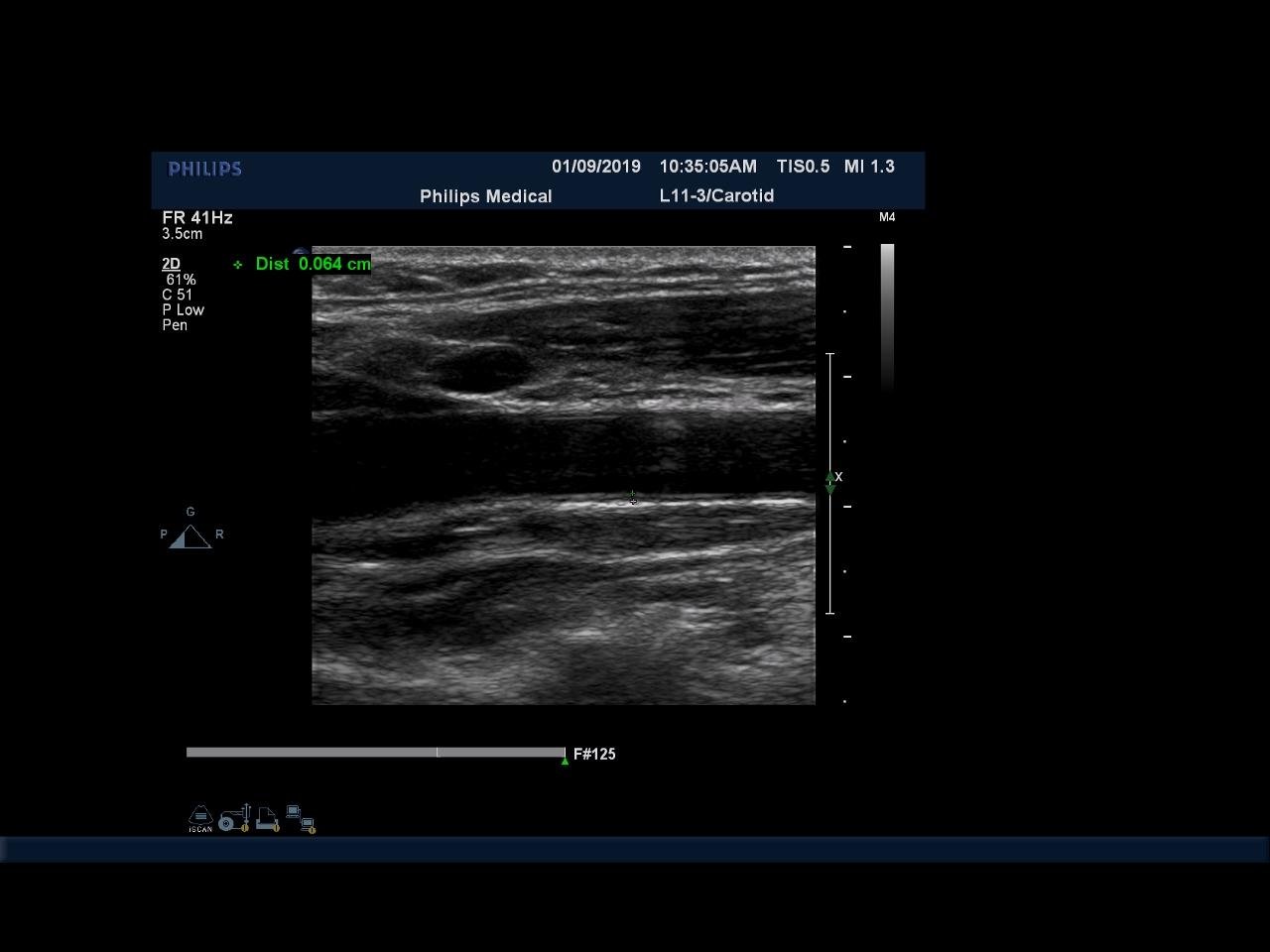

Supplement: Supplementary file 1 [file Data_Sheet_1.ZIP › CONTROLdate1/11.1.JPG]

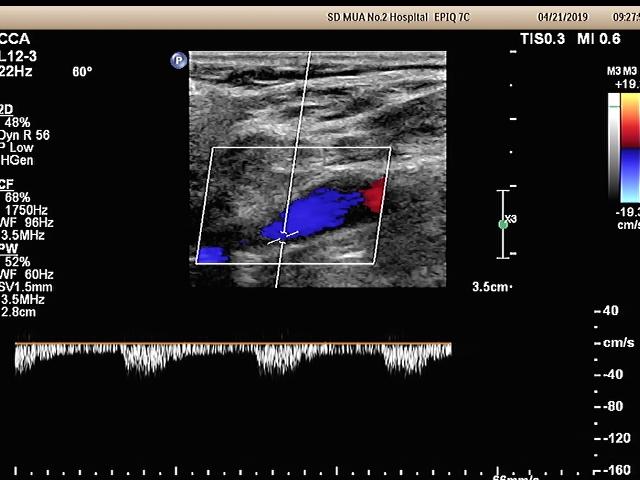

Supplement: Supplementary file 1 [file Data_Sheet_1.ZIP › CONTROLdate1/110.1.JPG]

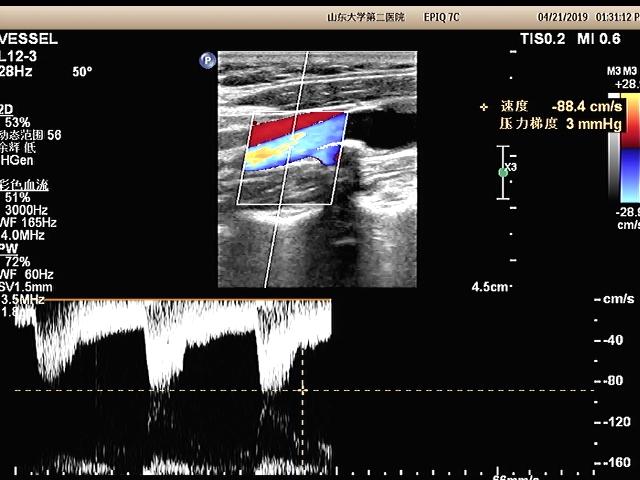

Supplement: Supplementary file 1 [file Data_Sheet_1.ZIP › CONTROLdate1/111.1.JPG]

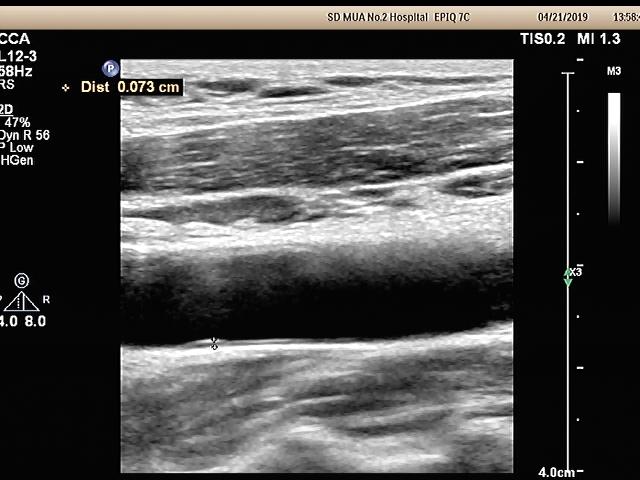

Supplement: Supplementary file 1 [file Data_Sheet_1.ZIP › CONTROLdate1/112.1.JPG]

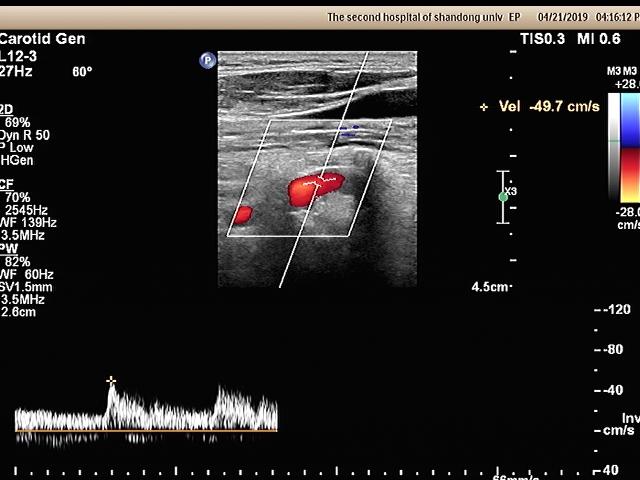

Supplement: Supplementary file 1 [file Data_Sheet_1.ZIP › CONTROLdate1/113.1.JPG]

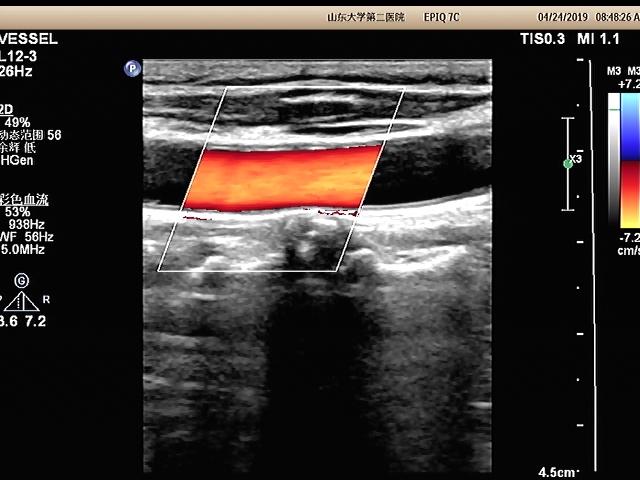

Supplement: Supplementary file 1 [file Data_Sheet_1.ZIP › CONTROLdate1/114.1.JPG]

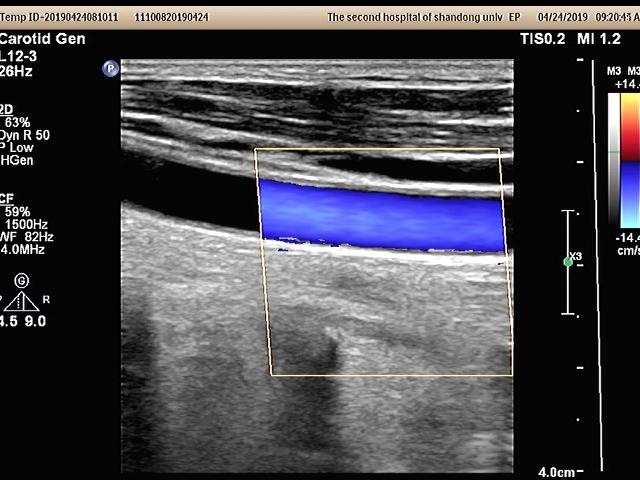

Supplement: Supplementary file 1 [file Data_Sheet_1.ZIP › CONTROLdate1/115.1.JPG]

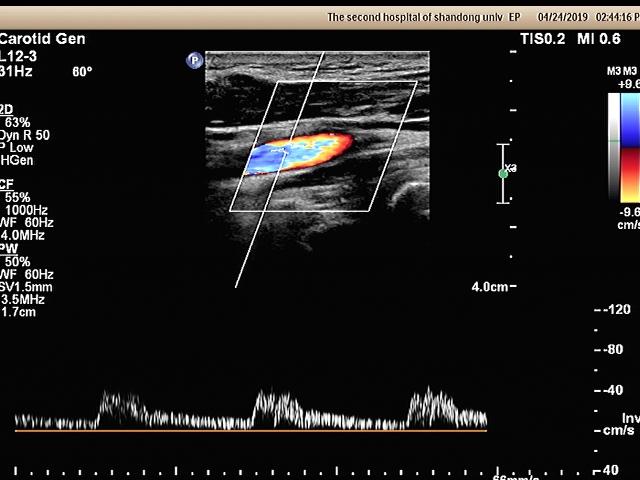

Supplement: Supplementary file 1 [file Data_Sheet_1.ZIP › CONTROLdate1/116.1.JPG]

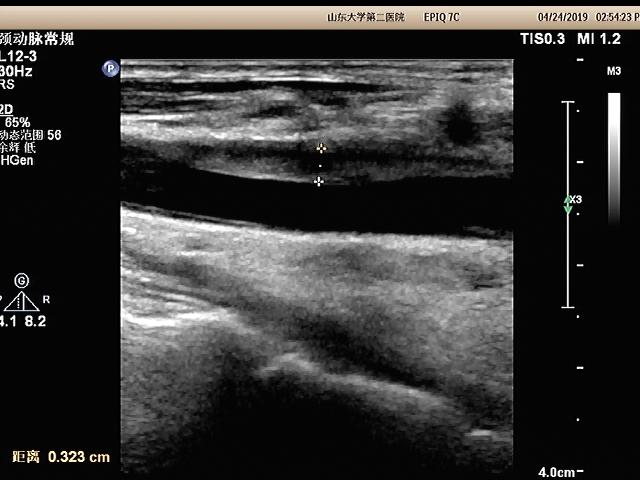

Supplement: Supplementary file 1 [file Data_Sheet_1.ZIP › CONTROLdate1/117.1.JPG]

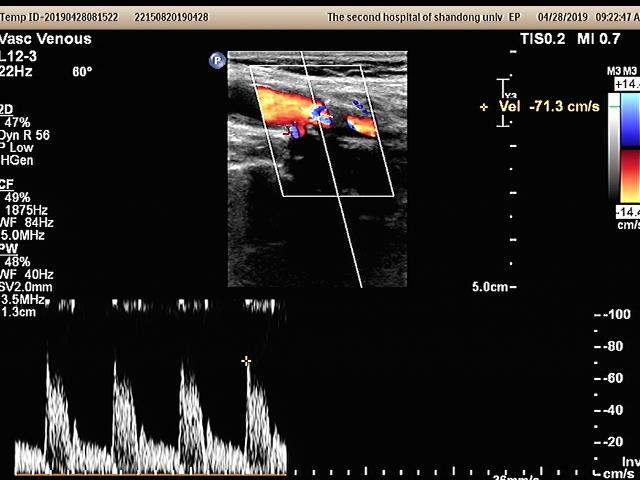

Supplement: Supplementary file 1 [file Data_Sheet_1.ZIP › CONTROLdate1/118.1.JPG]

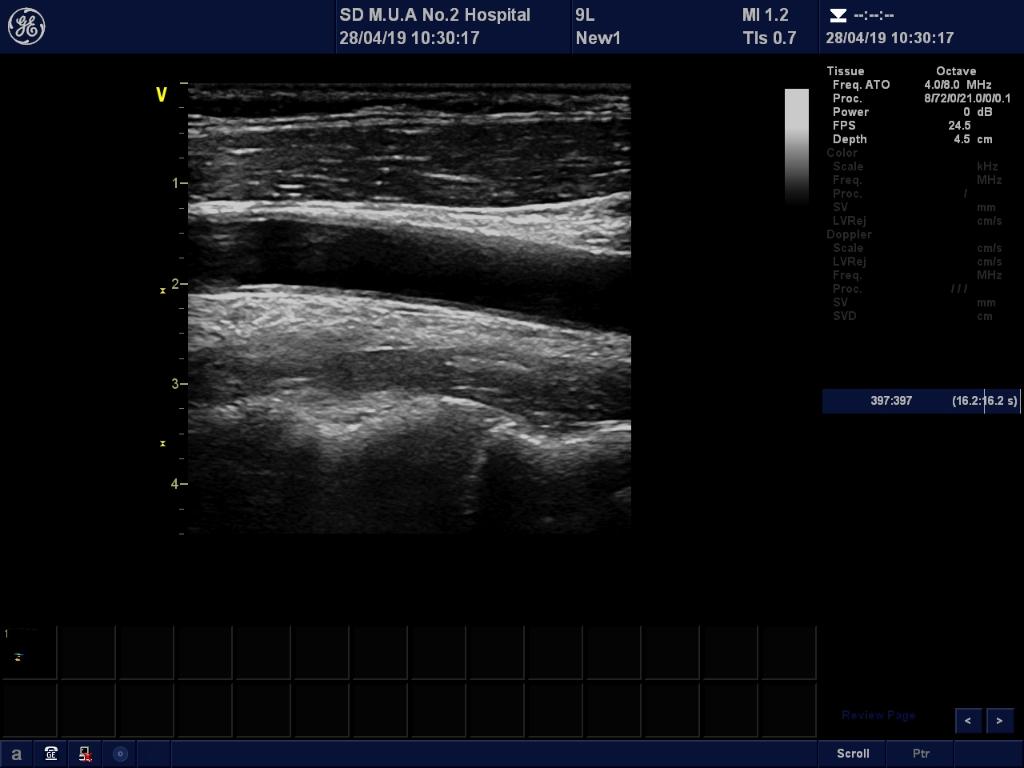

Supplement: Supplementary file 1 [file Data_Sheet_1.ZIP › CONTROLdate1/119.1.JPG]

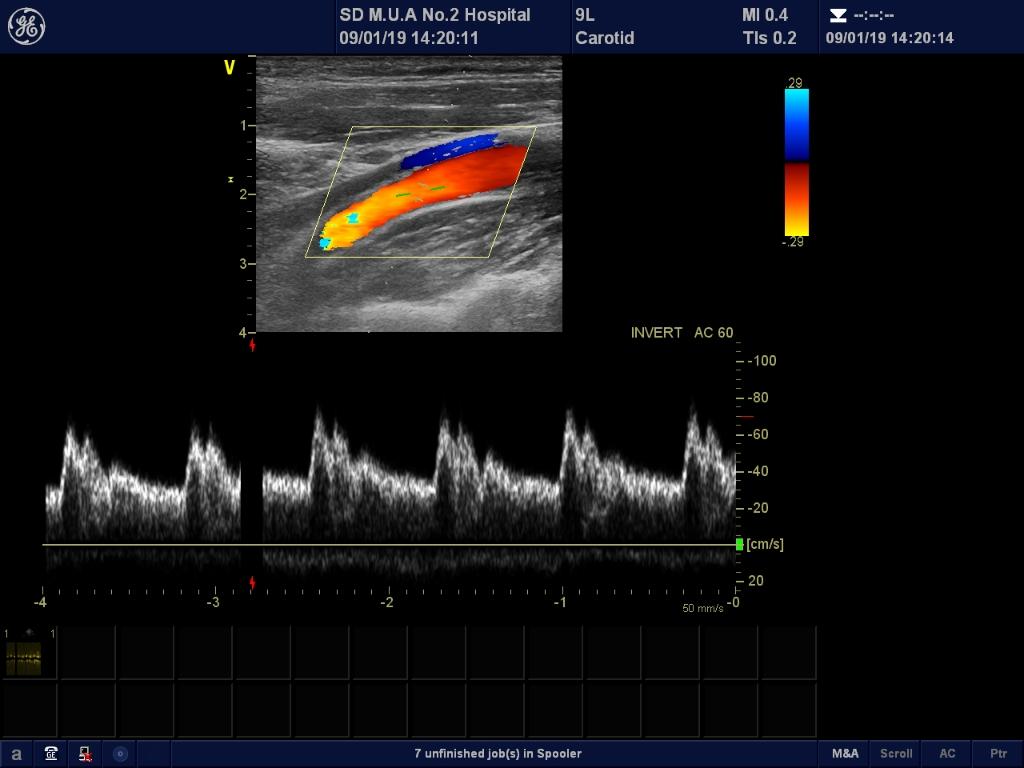

Supplement: Supplementary file 1 [file Data_Sheet_1.ZIP › CONTROLdate1/12.1.JPG]

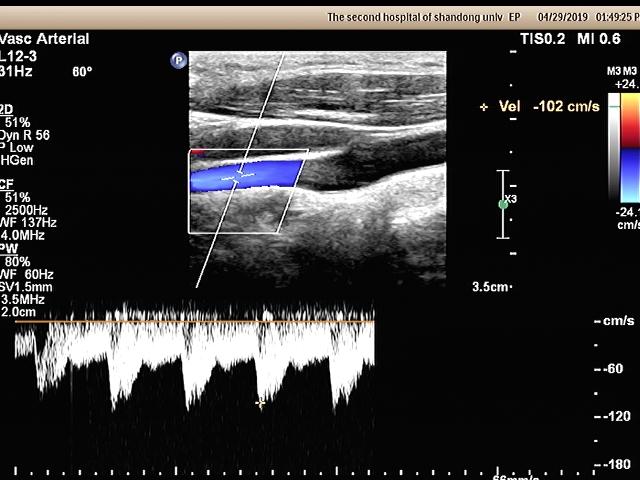

Supplement: Supplementary file 1 [file Data_Sheet_1.ZIP › CONTROLdate1/120.1.JPG]

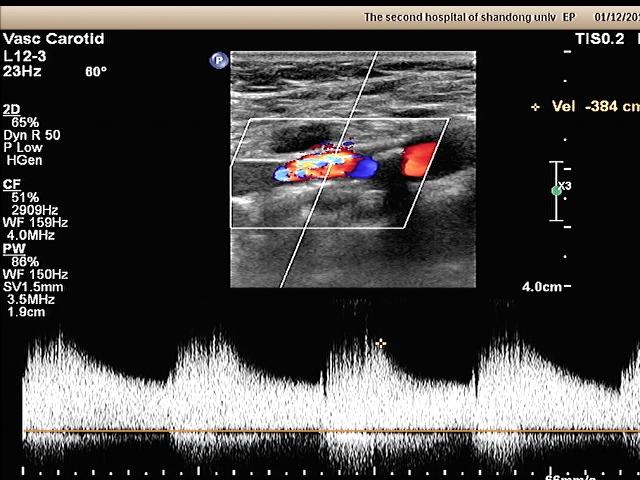

Supplement: Supplementary file 1 [file Data_Sheet_1.ZIP › CONTROLdate1/13.1.JPG]

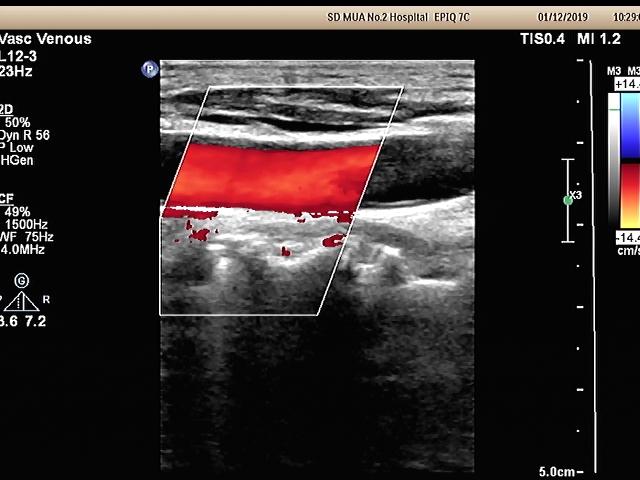

Supplement: Supplementary file 1 [file Data_Sheet_1.ZIP › CONTROLdate1/14.1.JPG]

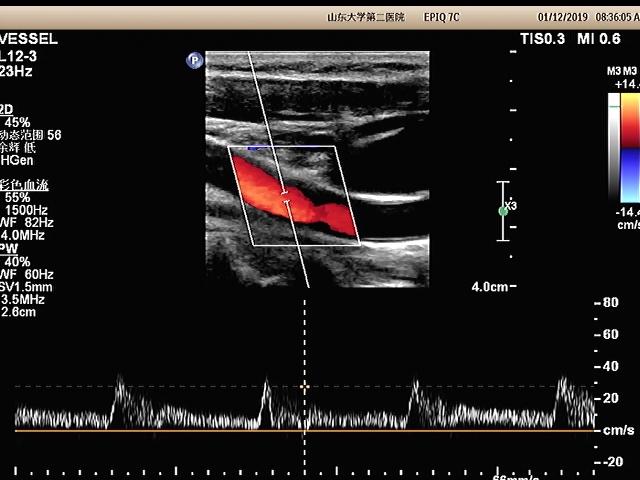

Supplement: Supplementary file 1 [file Data_Sheet_1.ZIP › CONTROLdate1/15.1.JPG]

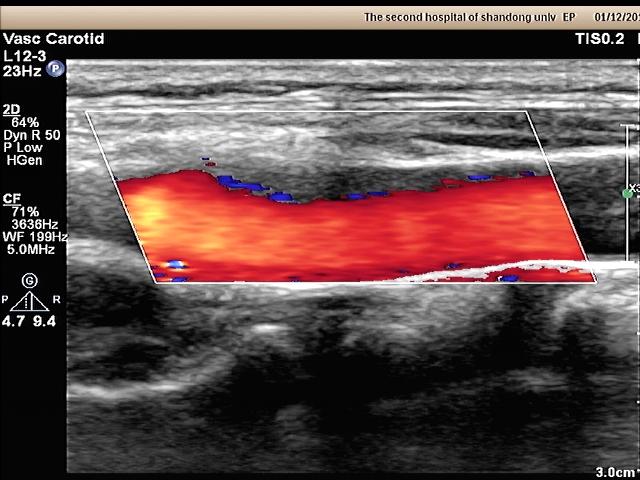

Supplement: Supplementary file 1 [file Data_Sheet_1.ZIP › CONTROLdate1/16.1.JPG]

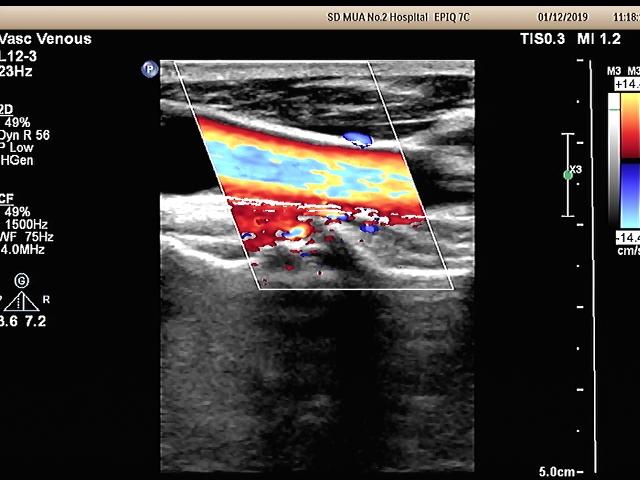

Supplement: Supplementary file 1 [file Data_Sheet_1.ZIP › CONTROLdate1/17.1.JPG]

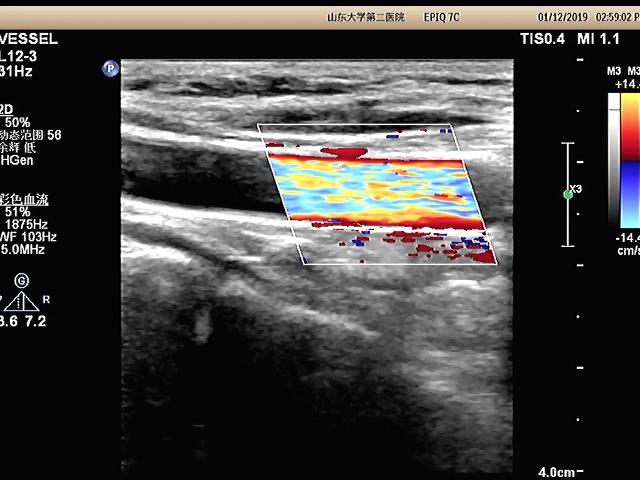

Supplement: Supplementary file 1 [file Data_Sheet_1.ZIP › CONTROLdate1/18.1.JPG]

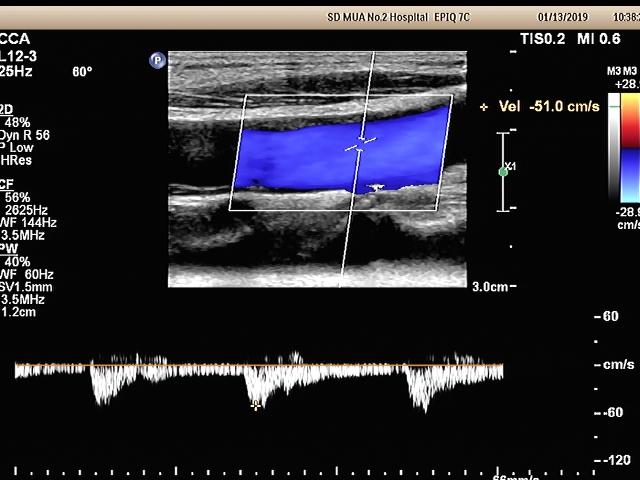

Supplement: Supplementary file 1 [file Data_Sheet_1.ZIP › CONTROLdate1/19.1.JPG]

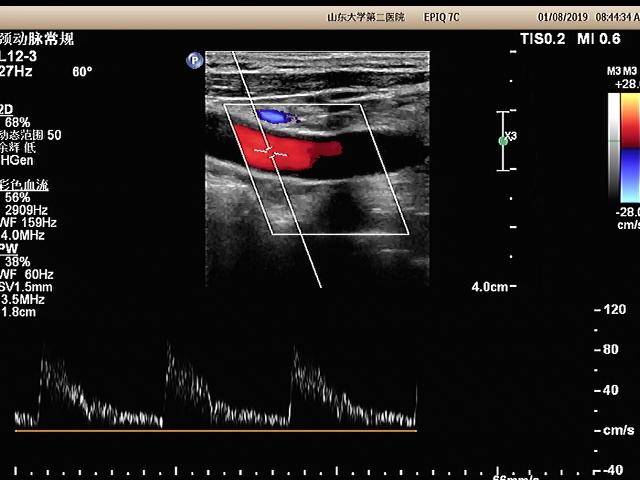

Supplement: Supplementary file 1 [file Data_Sheet_1.ZIP › CONTROLdate1/2.1.JPG]

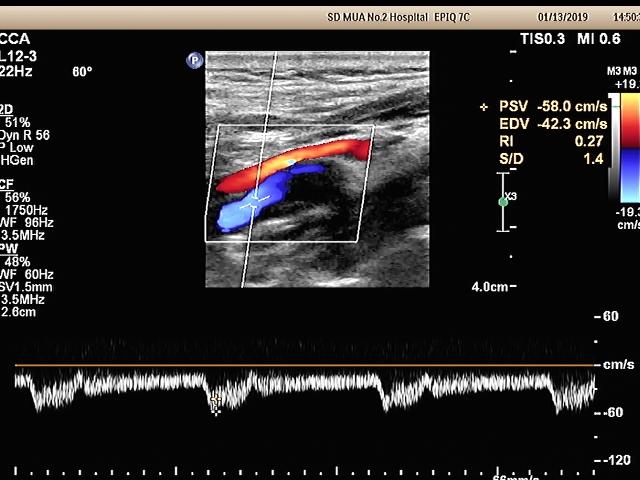

Supplement: Supplementary file 1 [file Data_Sheet_1.ZIP › CONTROLdate1/20.1.JPG]

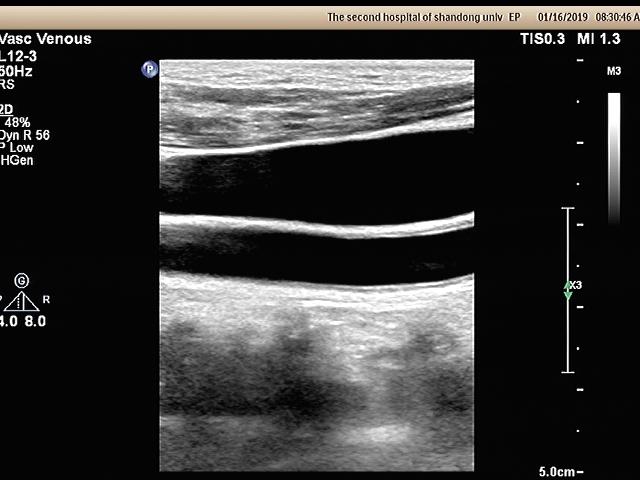

Supplement: Supplementary file 1 [file Data_Sheet_1.ZIP › CONTROLdate1/21.1.JPG]

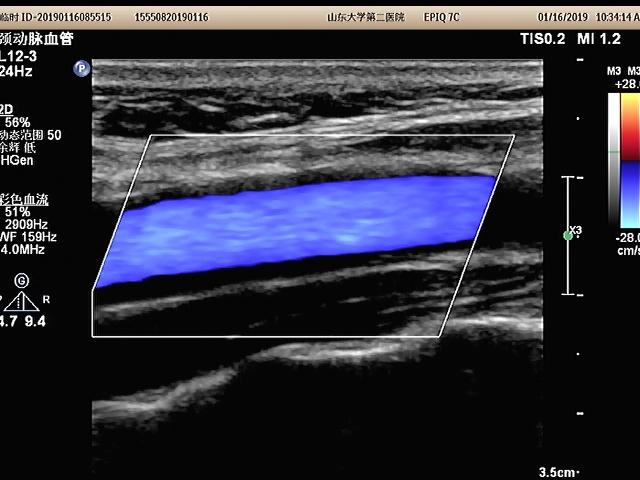

Supplement: Supplementary file 1 [file Data_Sheet_1.ZIP › CONTROLdate1/22.1.JPG]

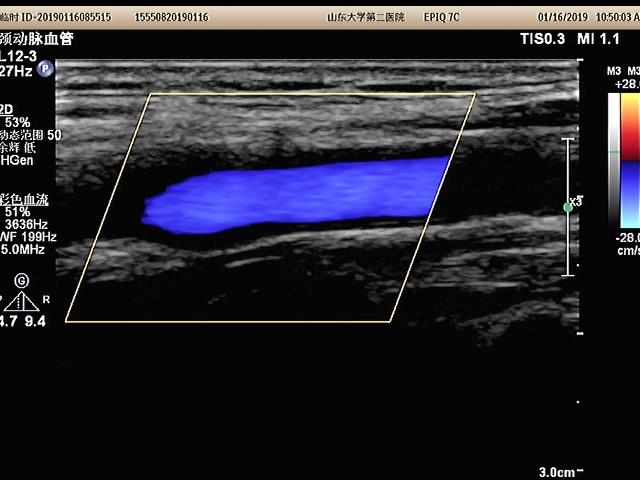

Supplement: Supplementary file 1 [file Data_Sheet_1.ZIP › CONTROLdate1/23.1.JPG]

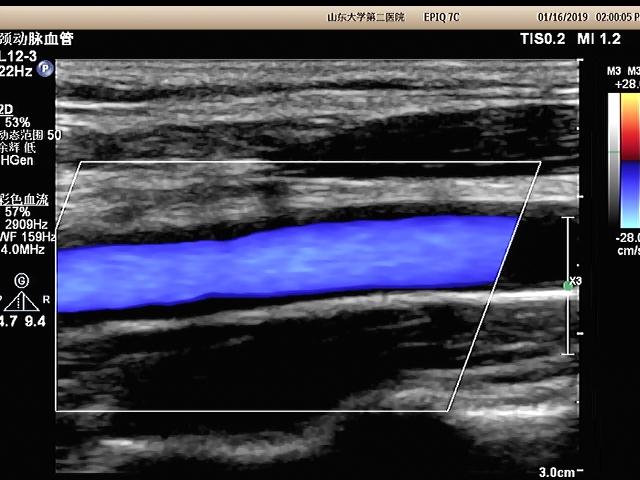

Supplement: Supplementary file 1 [file Data_Sheet_1.ZIP › CONTROLdate1/24.1.JPG]

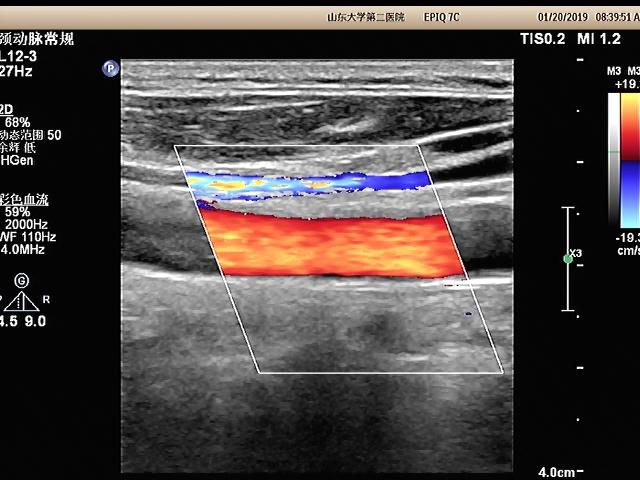

Supplement: Supplementary file 1 [file Data_Sheet_1.ZIP › CONTROLdate1/25.1.JPG]

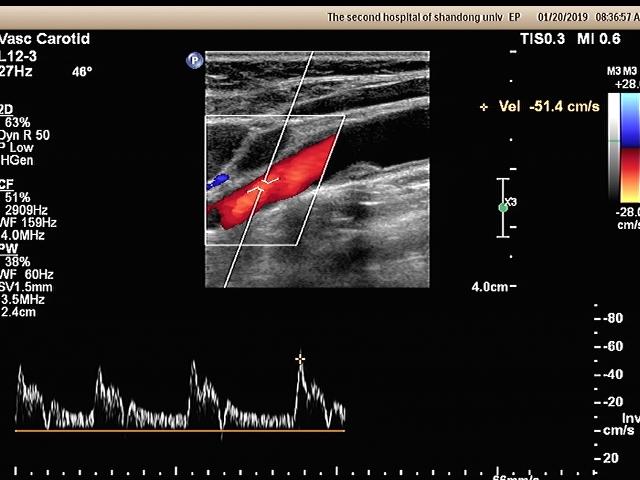

Supplement: Supplementary file 1 [file Data_Sheet_1.ZIP › CONTROLdate1/26.1.JPG]

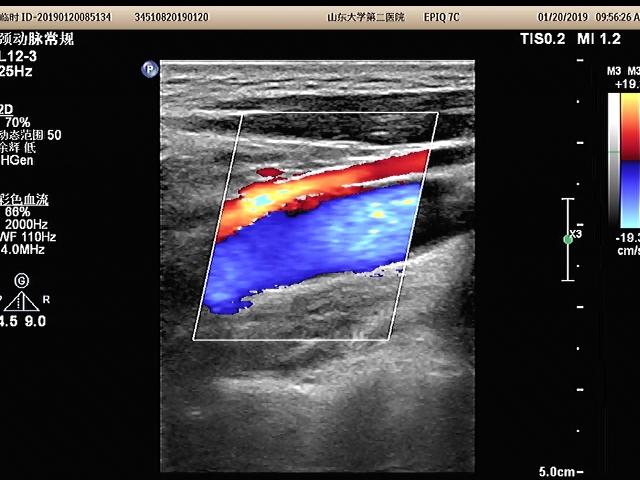

Supplement: Supplementary file 1 [file Data_Sheet_1.ZIP › CONTROLdate1/27.1.JPG]

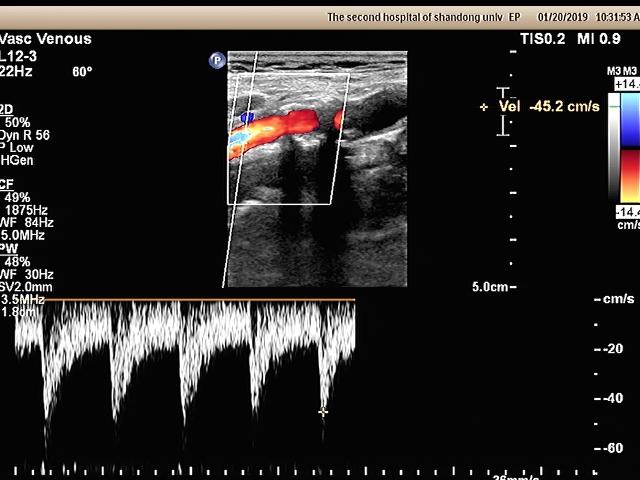

Supplement: Supplementary file 1 [file Data_Sheet_1.ZIP › CONTROLdate1/28.1.JPG]

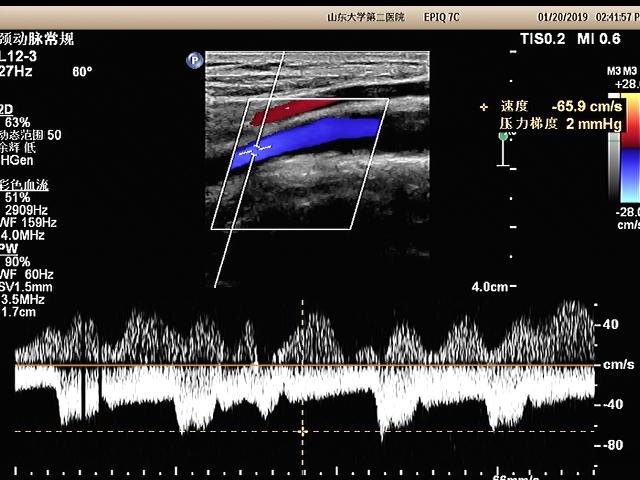

Supplement: Supplementary file 1 [file Data_Sheet_1.ZIP › CONTROLdate1/29.1.JPG]

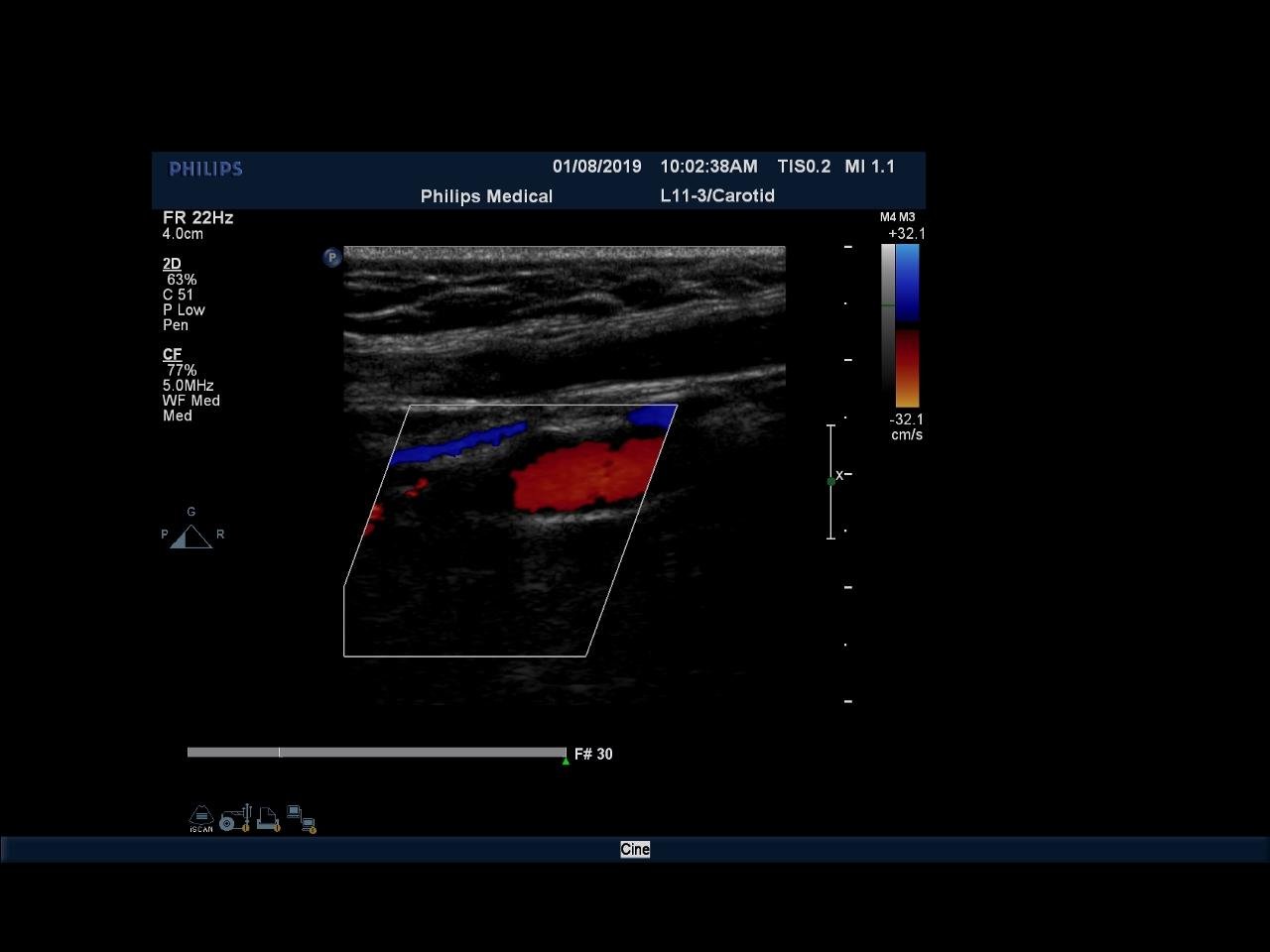

Supplement: Supplementary file 1 [file Data_Sheet_1.ZIP › CONTROLdate1/3.1.JPG]

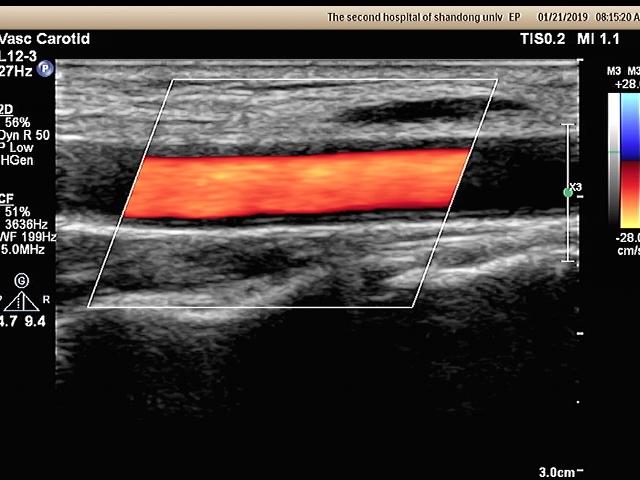

Supplement: Supplementary file 1 [file Data_Sheet_1.ZIP › CONTROLdate1/30.1.JPG]

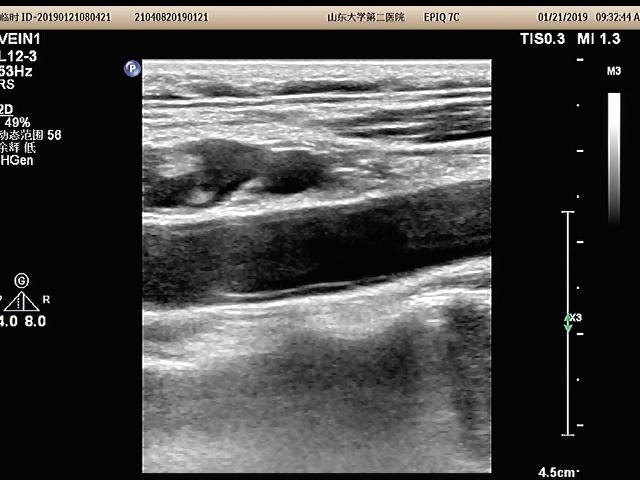

Supplement: Supplementary file 1 [file Data_Sheet_1.ZIP › CONTROLdate1/31.1.JPG]

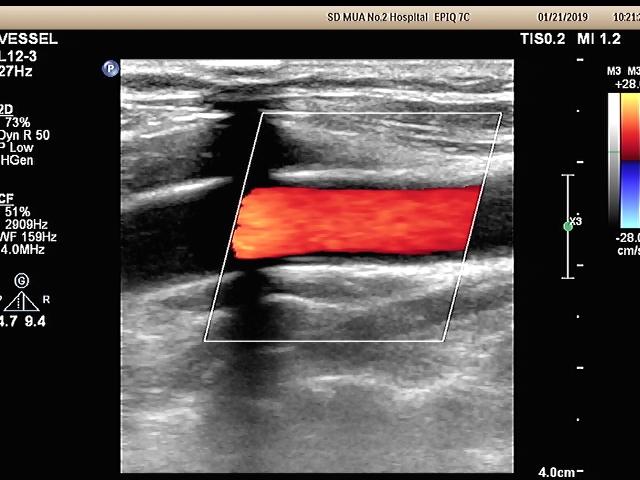

Supplement: Supplementary file 1 [file Data_Sheet_1.ZIP › CONTROLdate1/32.1.JPG]

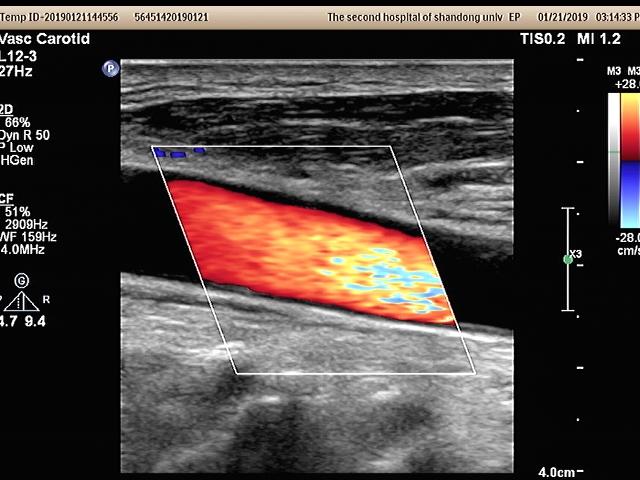

Supplement: Supplementary file 1 [file Data_Sheet_1.ZIP › CONTROLdate1/33.1.JPG]

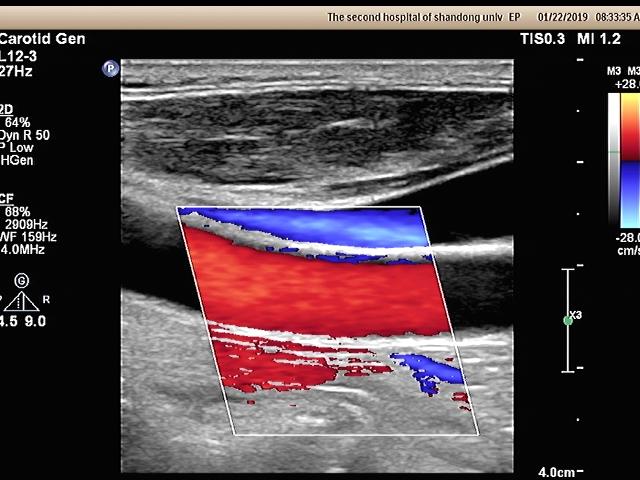

Supplement: Supplementary file 1 [file Data_Sheet_1.ZIP › CONTROLdate1/34.1.JPG]

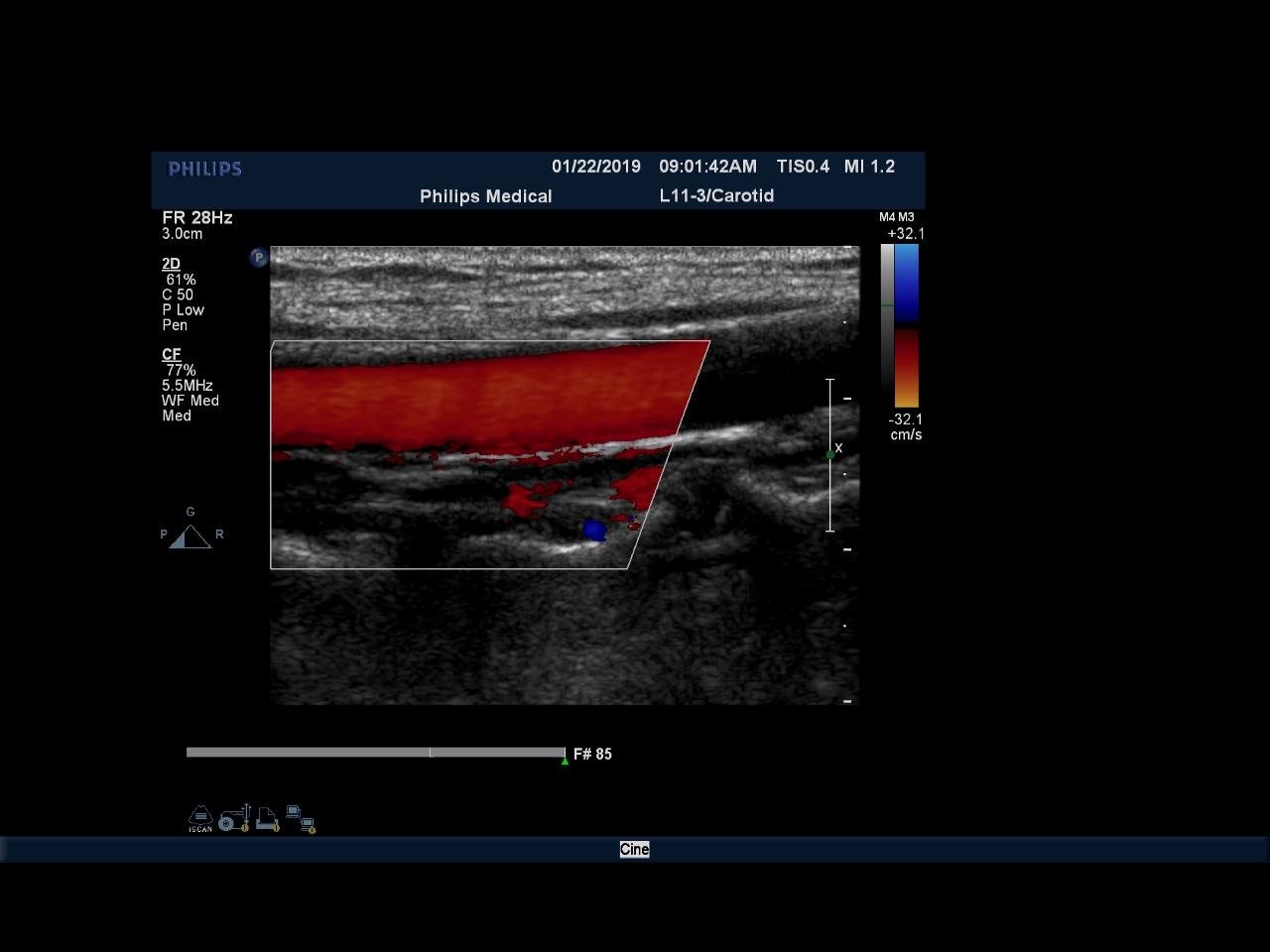

Supplement: Supplementary file 1 [file Data_Sheet_1.ZIP › CONTROLdate1/35.1.JPG]

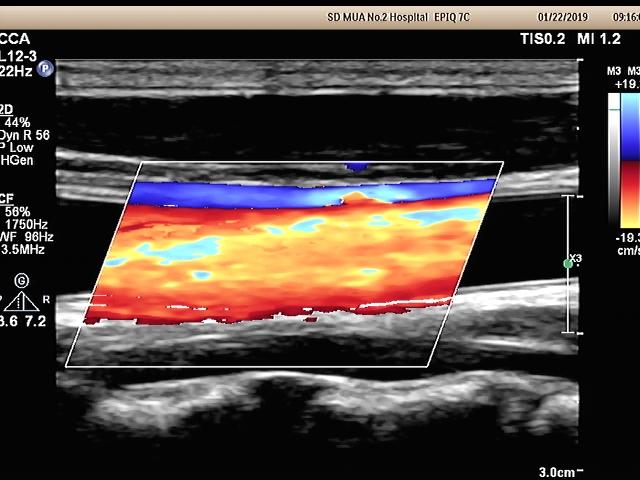

Supplement: Supplementary file 1 [file Data_Sheet_1.ZIP › CONTROLdate1/36.1.JPG]

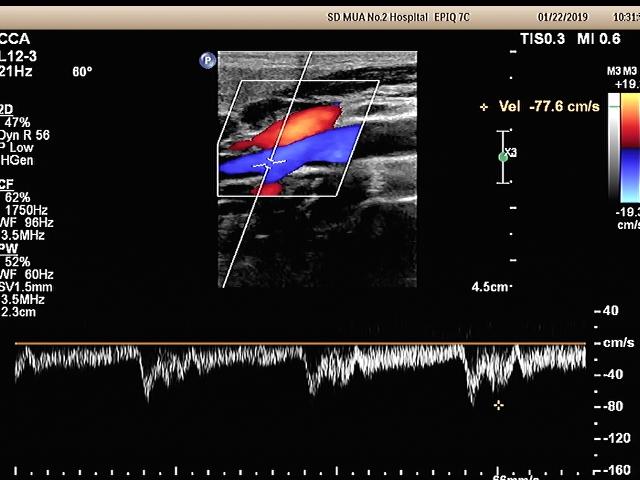

Supplement: Supplementary file 1 [file Data_Sheet_1.ZIP › CONTROLdate1/37.1.JPG]

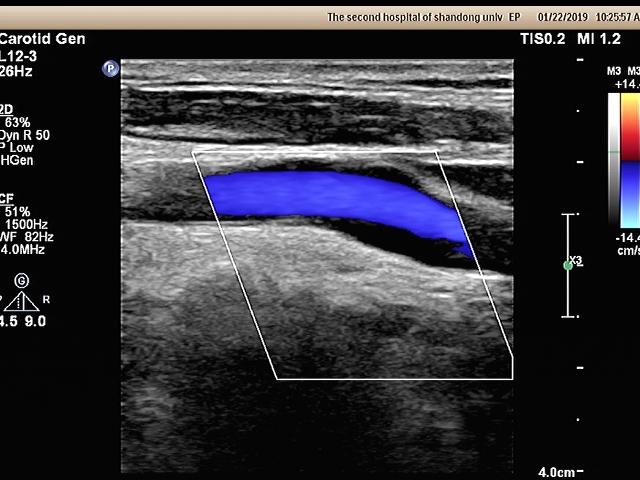

Supplement: Supplementary file 1 [file Data_Sheet_1.ZIP › CONTROLdate1/38.1.JPG]

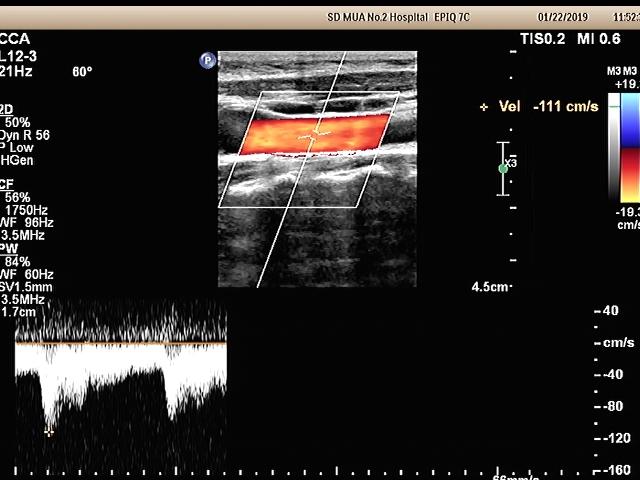

Supplement: Supplementary file 1 [file Data_Sheet_1.ZIP › CONTROLdate1/39.1.JPG]

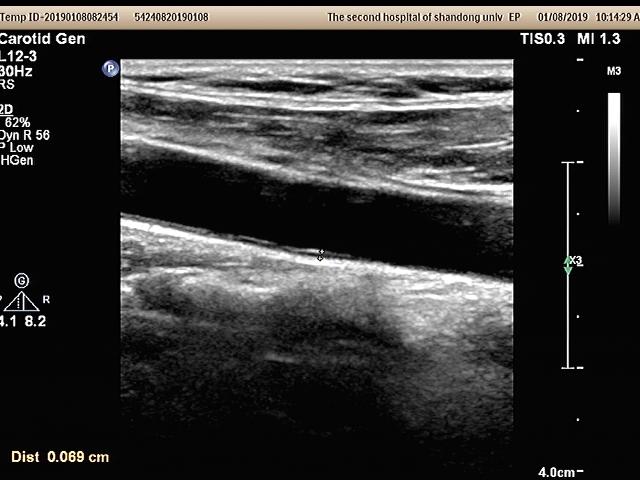

Supplement: Supplementary file 1 [file Data_Sheet_1.ZIP › CONTROLdate1/4.1.JPG]

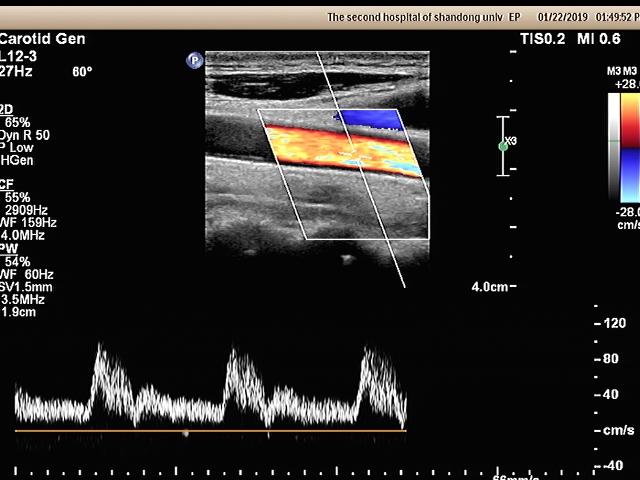

Supplement: Supplementary file 1 [file Data_Sheet_1.ZIP › CONTROLdate1/40.1.JPG]

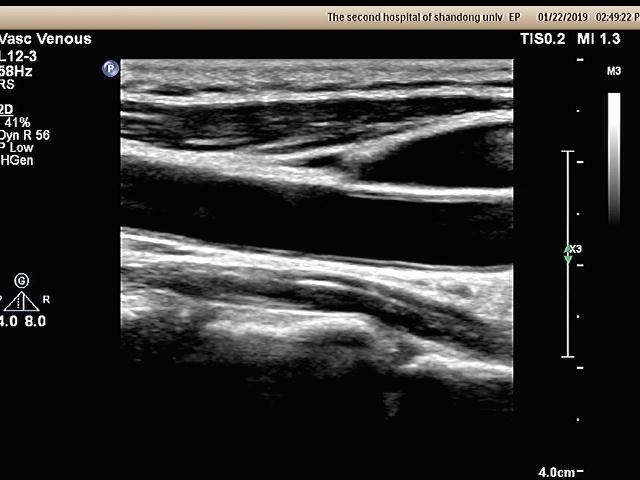

Supplement: Supplementary file 1 [file Data_Sheet_1.ZIP › CONTROLdate1/41.1.JPG]

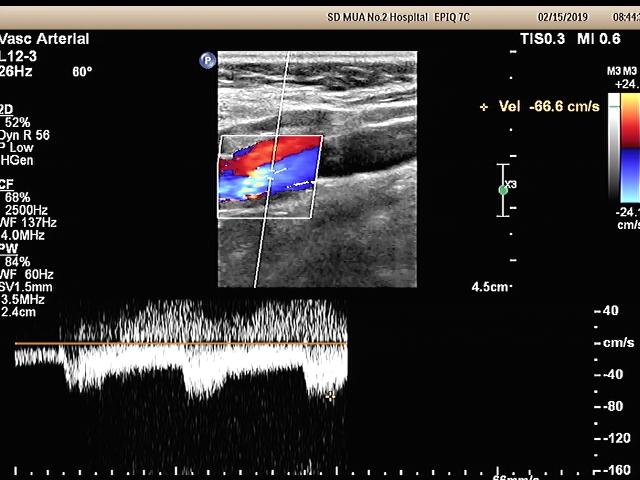

Supplement: Supplementary file 1 [file Data_Sheet_1.ZIP › CONTROLdate1/42.1.JPG]

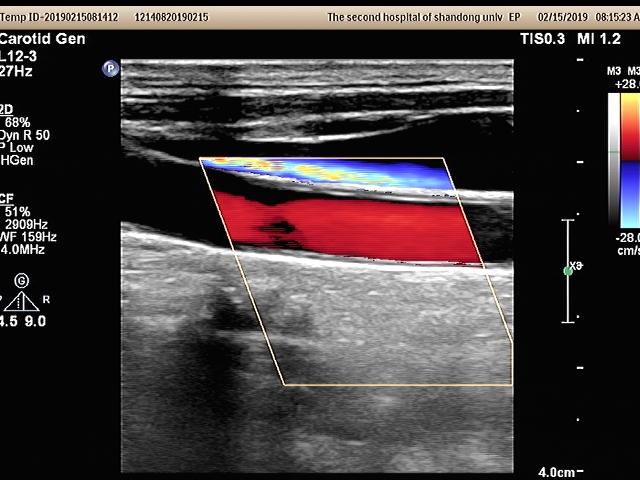

Supplement: Supplementary file 1 [file Data_Sheet_1.ZIP › CONTROLdate1/43.1.JPG]

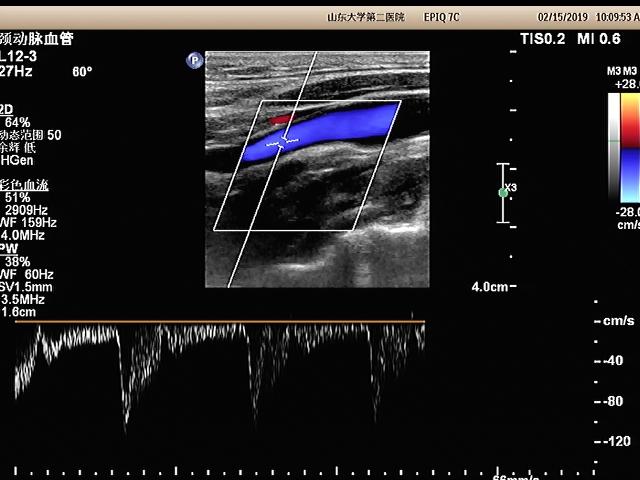

Supplement: Supplementary file 1 [file Data_Sheet_1.ZIP › CONTROLdate1/44.1.JPG]

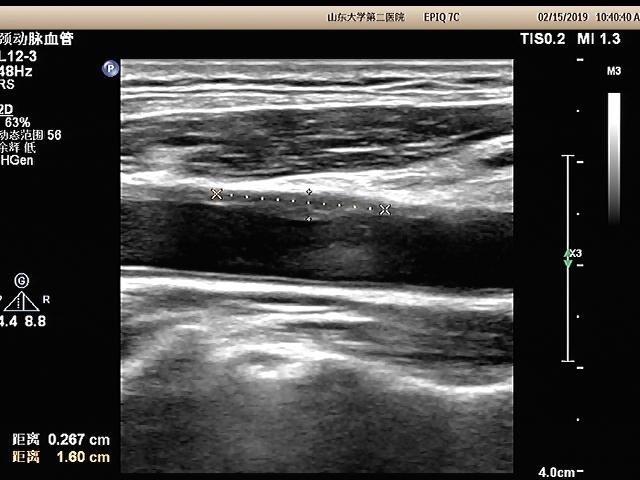

Supplement: Supplementary file 1 [file Data_Sheet_1.ZIP › CONTROLdate1/45.1.JPG]

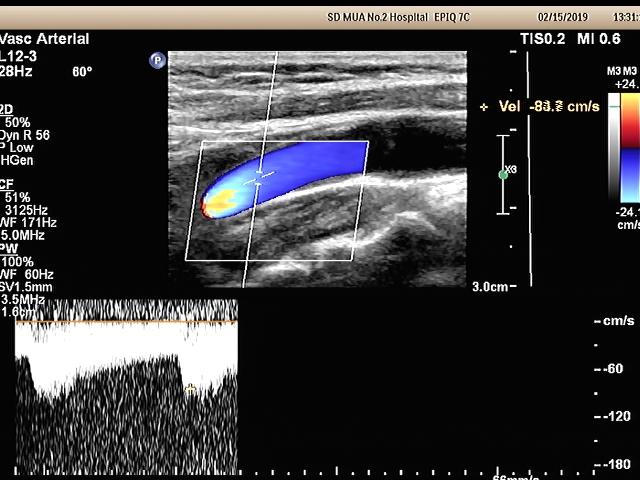

Supplement: Supplementary file 1 [file Data_Sheet_1.ZIP › CONTROLdate1/46.1.JPG]

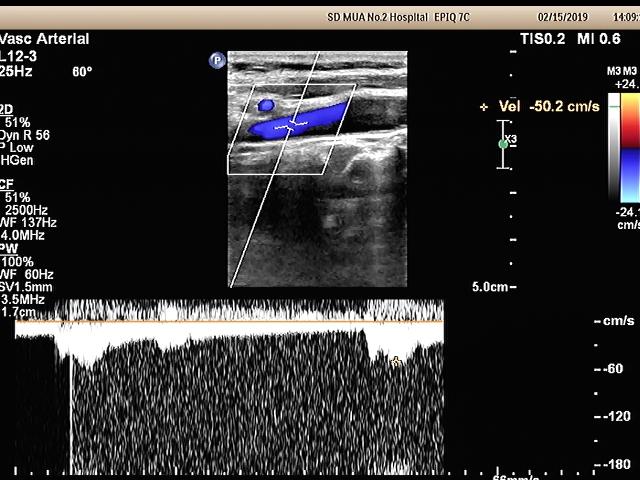

Supplement: Supplementary file 1 [file Data_Sheet_1.ZIP › CONTROLdate1/47.1.JPG]

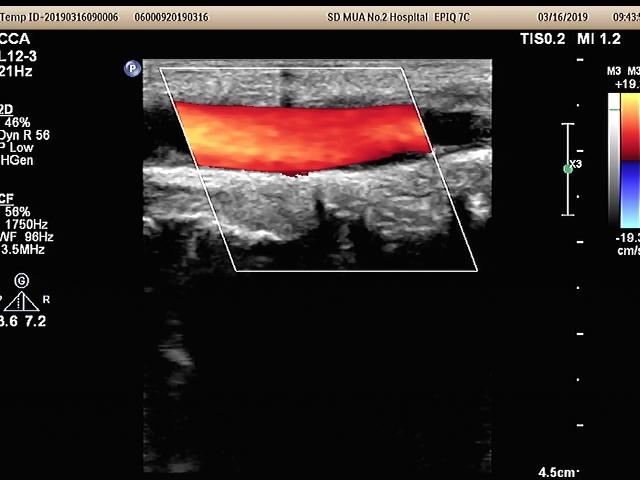

Supplement: Supplementary file 1 [file Data_Sheet_1.ZIP › CONTROLdate1/48.1.JPG]

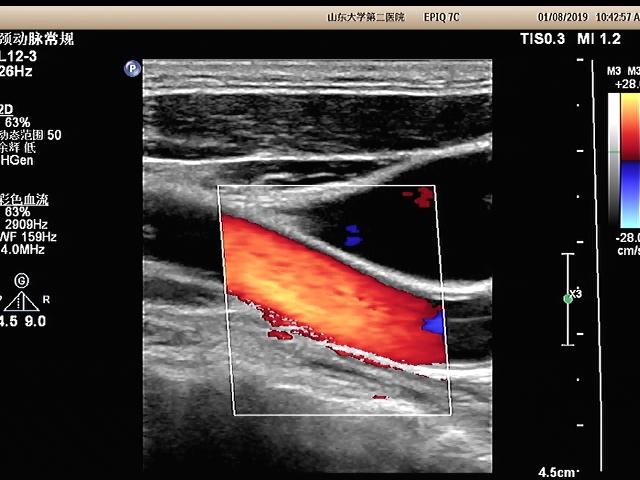

Supplement: Supplementary file 1 [file Data_Sheet_1.ZIP › CONTROLdate1/5.1.JPG]

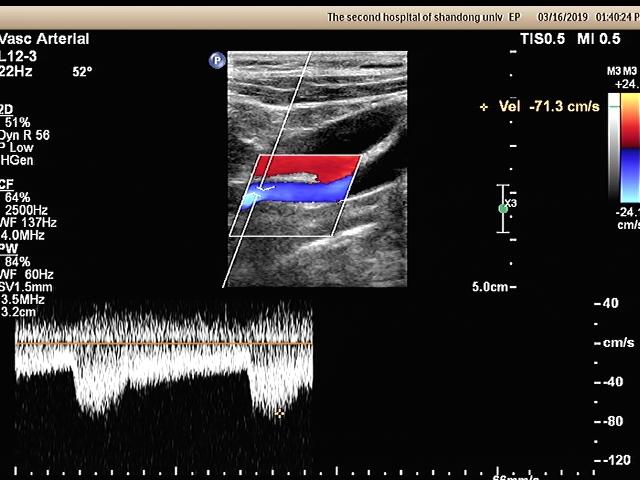

Supplement: Supplementary file 1 [file Data_Sheet_1.ZIP › CONTROLdate1/50.1.JPG]

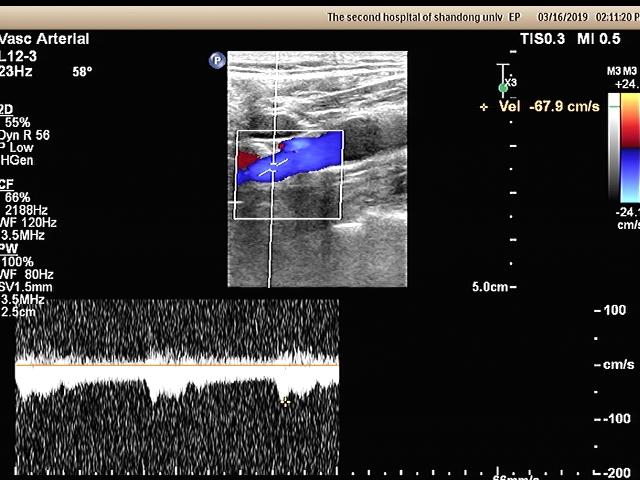

Supplement: Supplementary file 1 [file Data_Sheet_1.ZIP › CONTROLdate1/51.1.JPG]

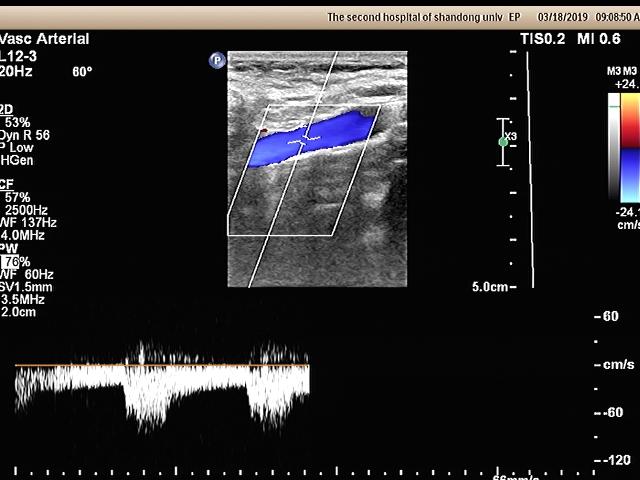

Supplement: Supplementary file 1 [file Data_Sheet_1.ZIP › CONTROLdate1/52.1.JPG]

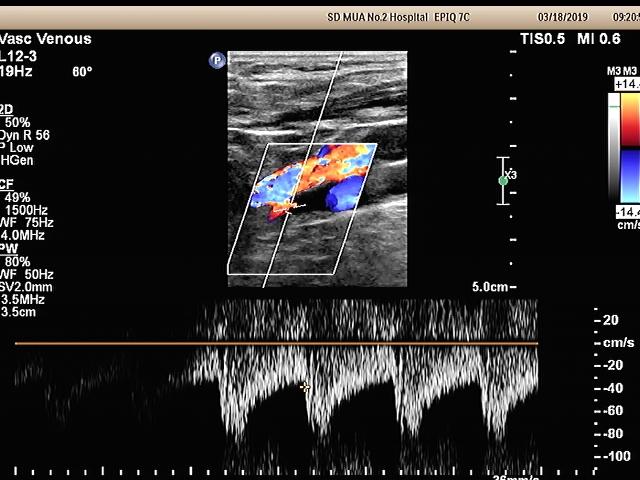

Supplement: Supplementary file 1 [file Data_Sheet_1.ZIP › CONTROLdate1/53.1.JPG]

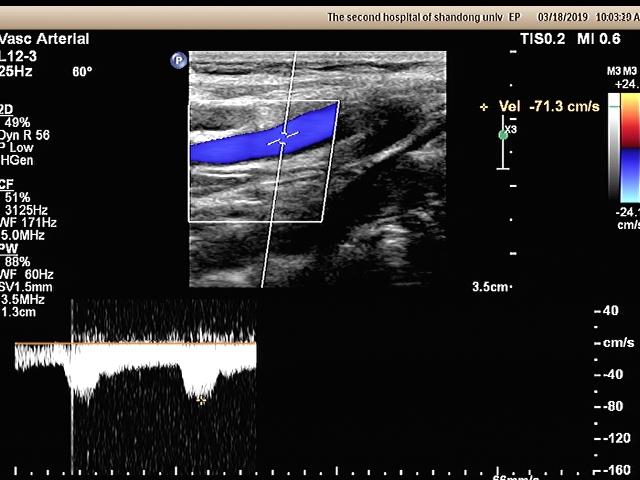

Supplement: Supplementary file 1 [file Data_Sheet_1.ZIP › CONTROLdate1/54.1.JPG]

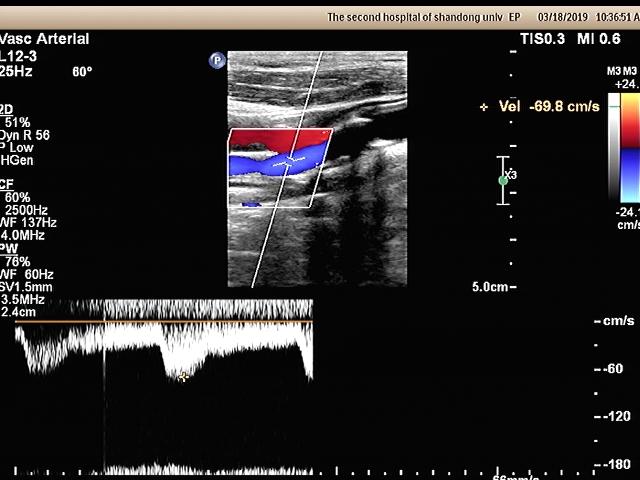

Supplement: Supplementary file 1 [file Data_Sheet_1.ZIP › CONTROLdate1/55.1.JPG]

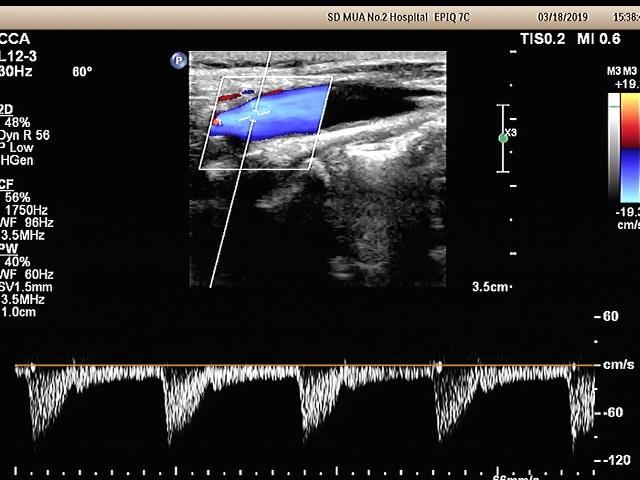

Supplement: Supplementary file 1 [file Data_Sheet_1.ZIP › CONTROLdate1/56.1.JPG]

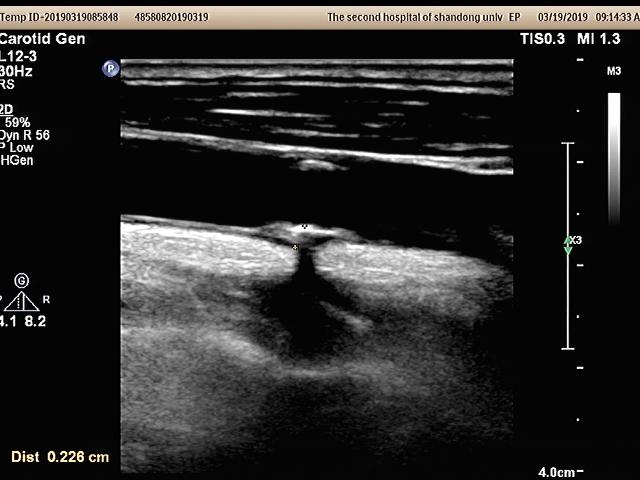

Supplement: Supplementary file 1 [file Data_Sheet_1.ZIP › CONTROLdate1/57.1.JPG]

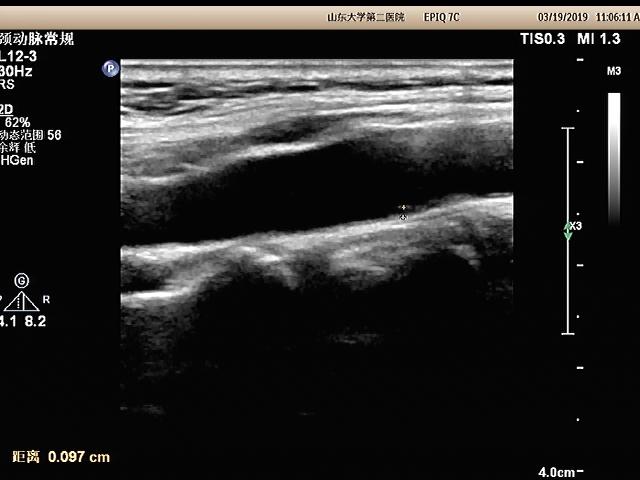

Supplement: Supplementary file 1 [file Data_Sheet_1.ZIP › CONTROLdate1/58.1.JPG]

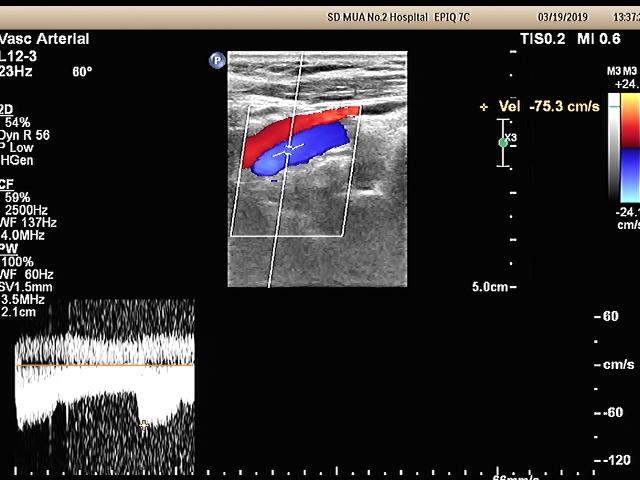

Supplement: Supplementary file 1 [file Data_Sheet_1.ZIP › CONTROLdate1/59.1.JPG]

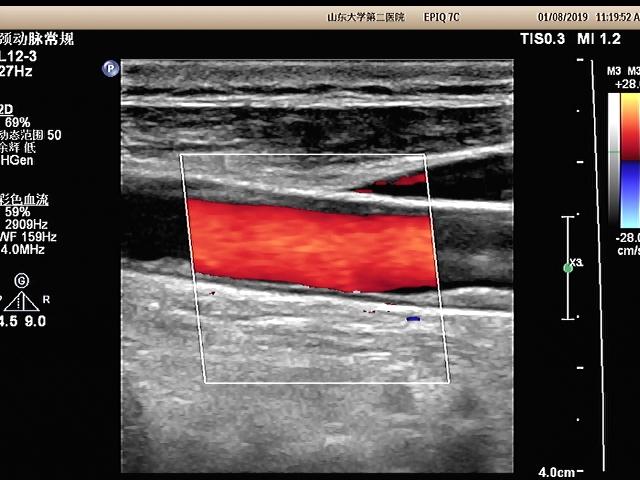

Supplement: Supplementary file 1 [file Data_Sheet_1.ZIP › CONTROLdate1/6.1.JPG]

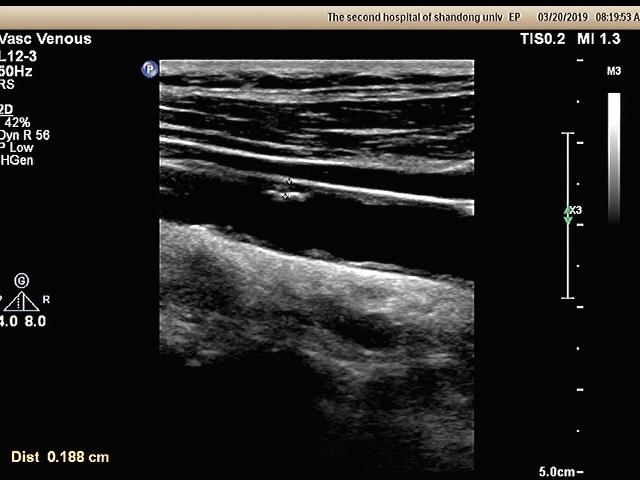

Supplement: Supplementary file 1 [file Data_Sheet_1.ZIP › CONTROLdate1/60.1.JPG]

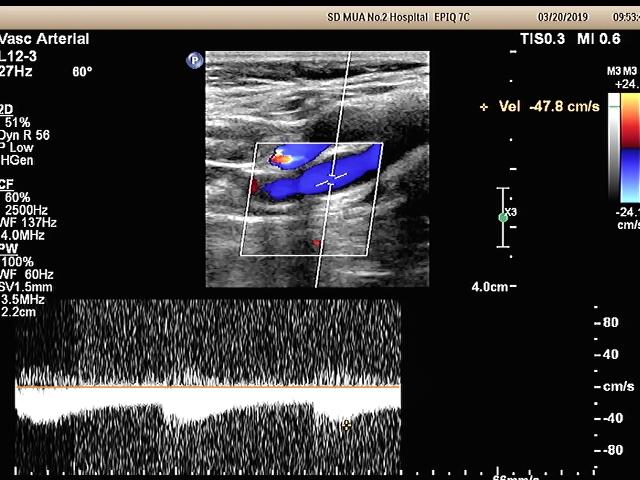

Supplement: Supplementary file 1 [file Data_Sheet_1.ZIP › CONTROLdate1/61.1.JPG]

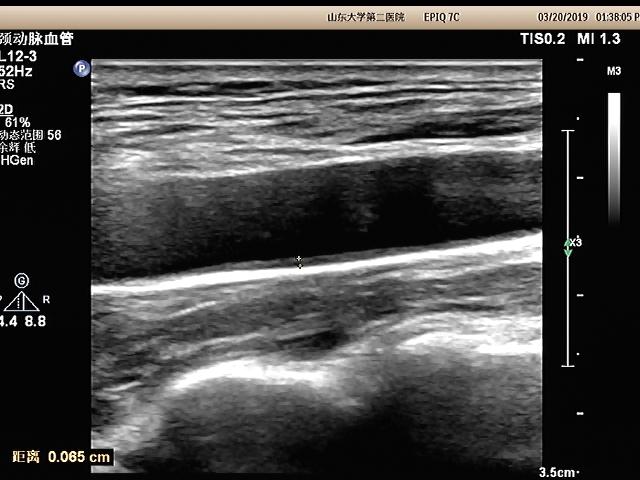

Supplement: Supplementary file 1 [file Data_Sheet_1.ZIP › CONTROLdate1/62.1.JPG]

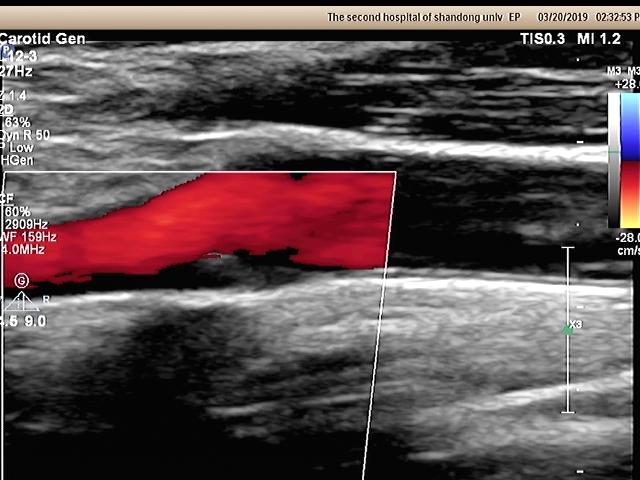

Supplement: Supplementary file 1 [file Data_Sheet_1.ZIP › CONTROLdate1/63.1.JPG]

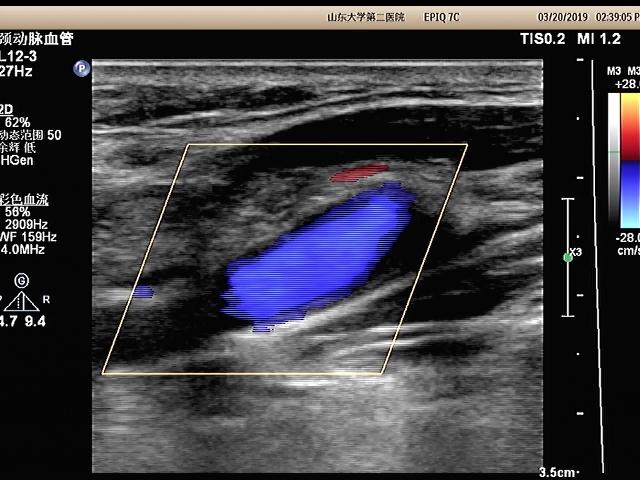

Supplement: Supplementary file 1 [file Data_Sheet_1.ZIP › CONTROLdate1/64.1.JPG]

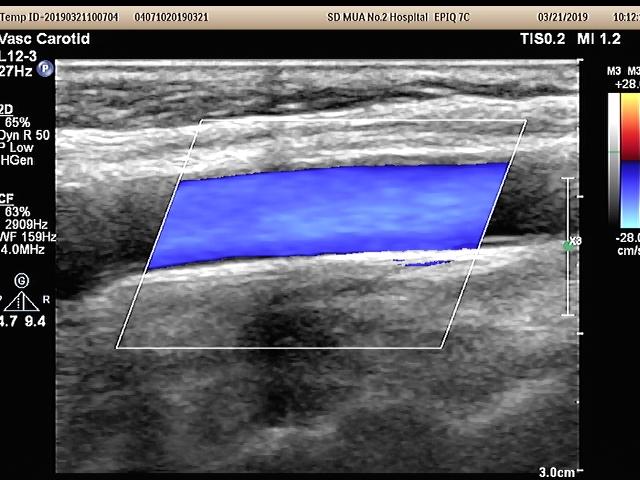

Supplement: Supplementary file 1 [file Data_Sheet_1.ZIP › CONTROLdate1/65.1.JPG]

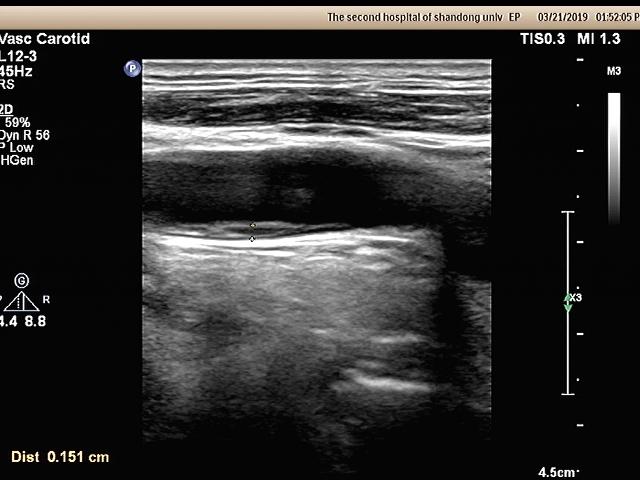

Supplement: Supplementary file 1 [file Data_Sheet_1.ZIP › CONTROLdate1/66.1.JPG]

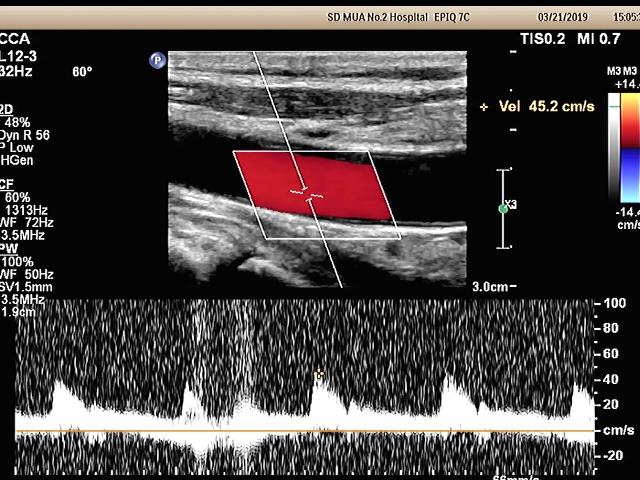

Supplement: Supplementary file 1 [file Data_Sheet_1.ZIP › CONTROLdate1/67.1.JPG]

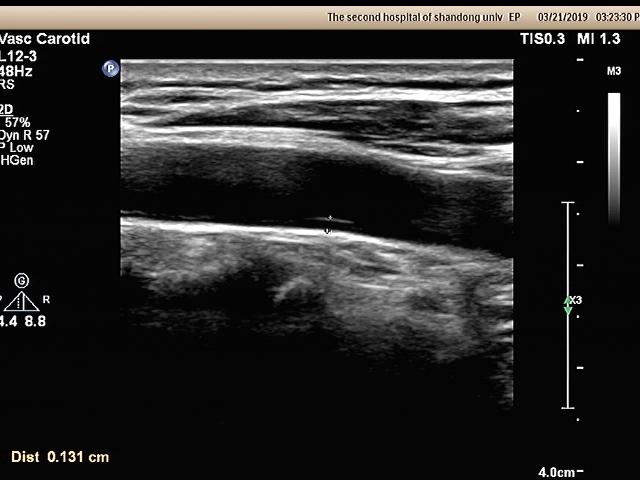

Supplement: Supplementary file 1 [file Data_Sheet_1.ZIP › CONTROLdate1/68.1.JPG]

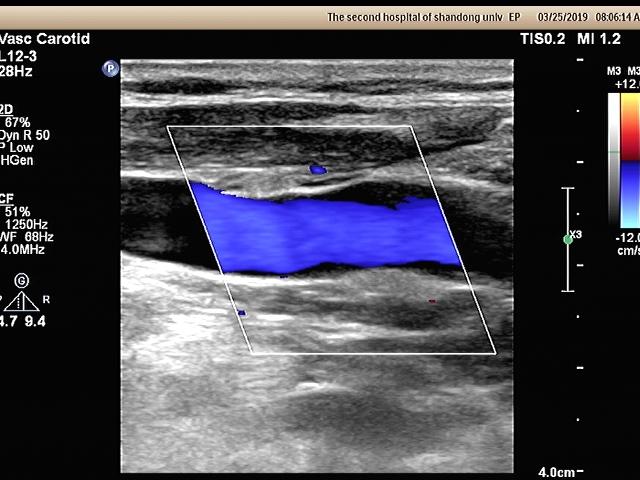

Supplement: Supplementary file 1 [file Data_Sheet_1.ZIP › CONTROLdate1/69.1.JPG]

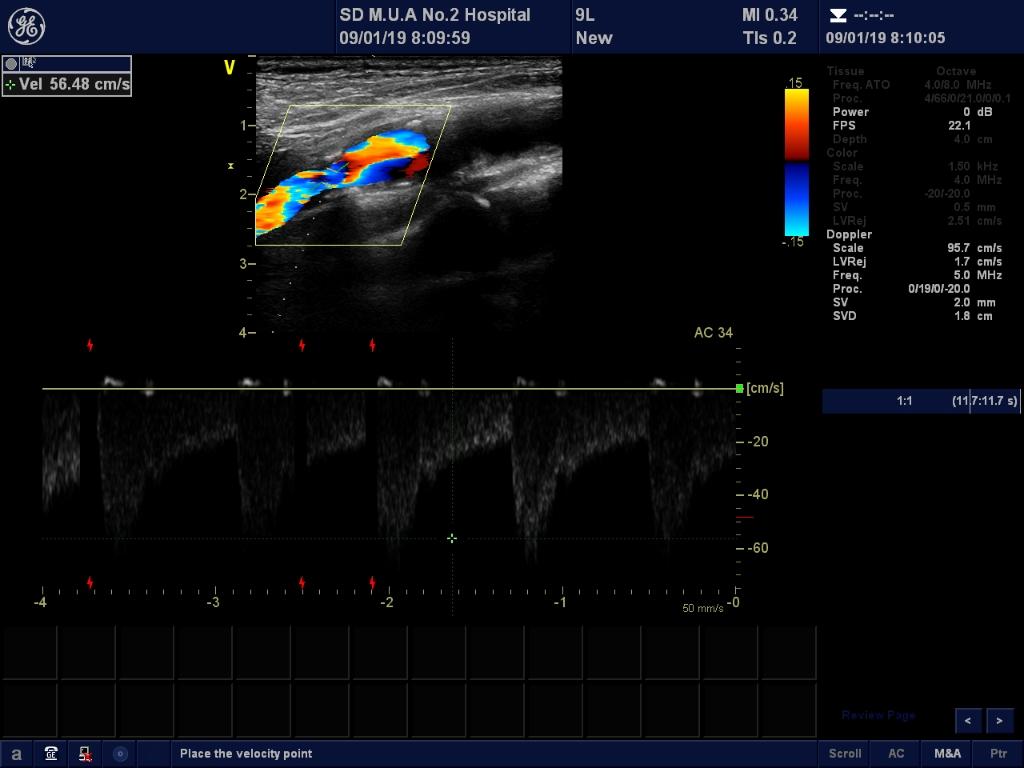

Supplement: Supplementary file 1 [file Data_Sheet_1.ZIP › CONTROLdate1/7.1.JPG]

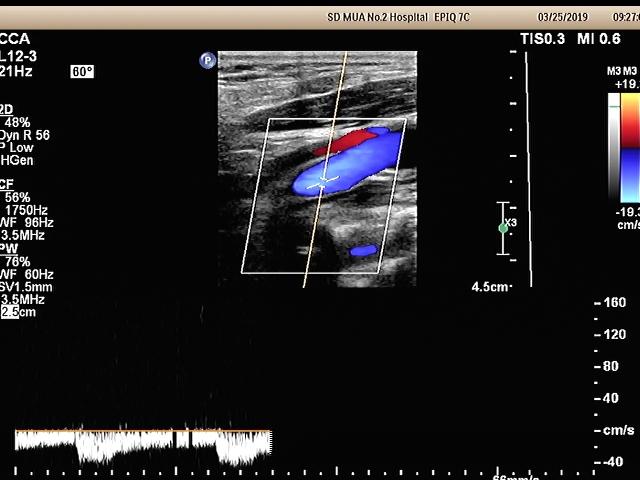

Supplement: Supplementary file 1 [file Data_Sheet_1.ZIP › CONTROLdate1/70.1.JPG]

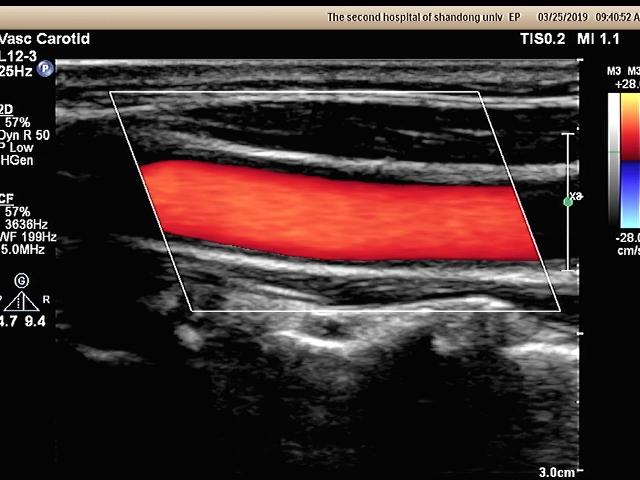

Supplement: Supplementary file 1 [file Data_Sheet_1.ZIP › CONTROLdate1/71.1.JPG]

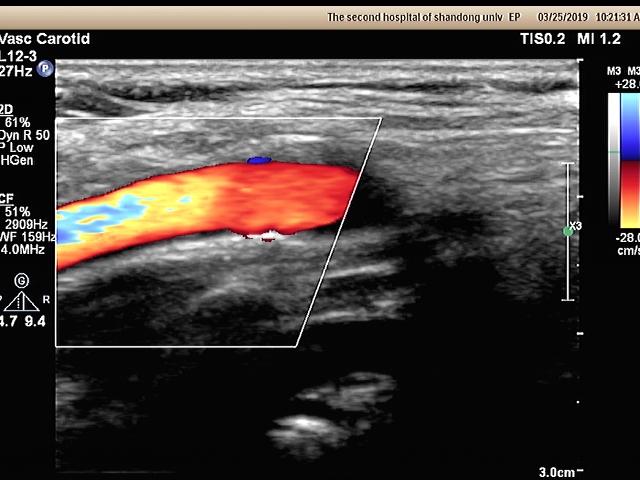

Supplement: Supplementary file 1 [file Data_Sheet_1.ZIP › CONTROLdate1/72.1.JPG]

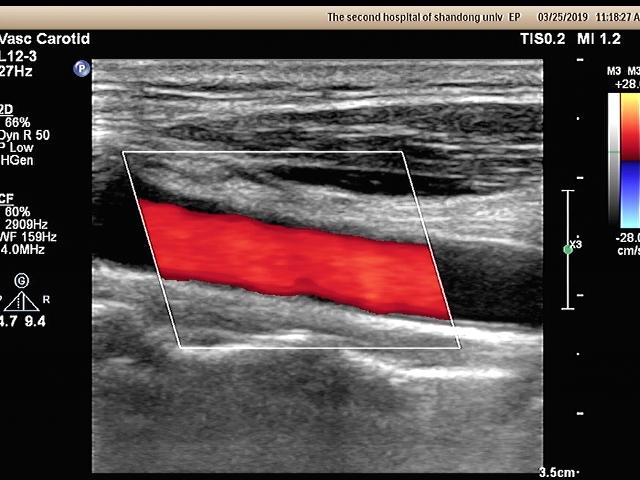

Supplement: Supplementary file 1 [file Data_Sheet_1.ZIP › CONTROLdate1/73.1.JPG]

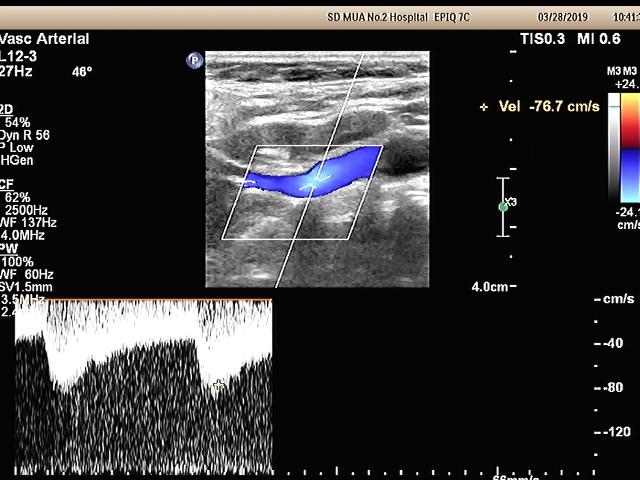

Supplement: Supplementary file 1 [file Data_Sheet_1.ZIP › CONTROLdate1/74.1.JPG]

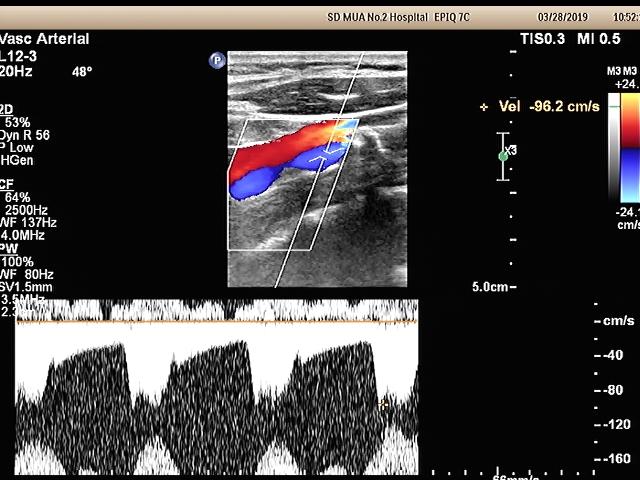

Supplement: Supplementary file 1 [file Data_Sheet_1.ZIP › CONTROLdate1/75.1.JPG]

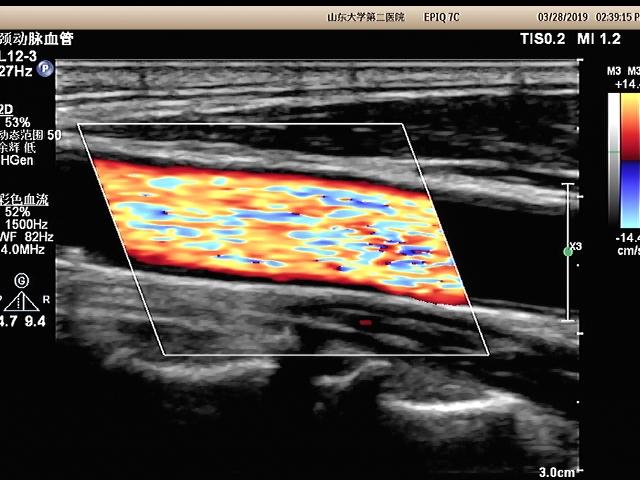

Supplement: Supplementary file 1 [file Data_Sheet_1.ZIP › CONTROLdate1/76.1.JPG]

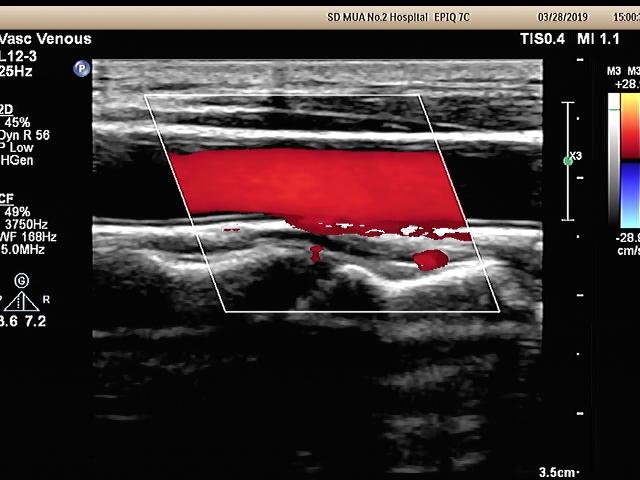

Supplement: Supplementary file 1 [file Data_Sheet_1.ZIP › CONTROLdate1/77.1.JPG]

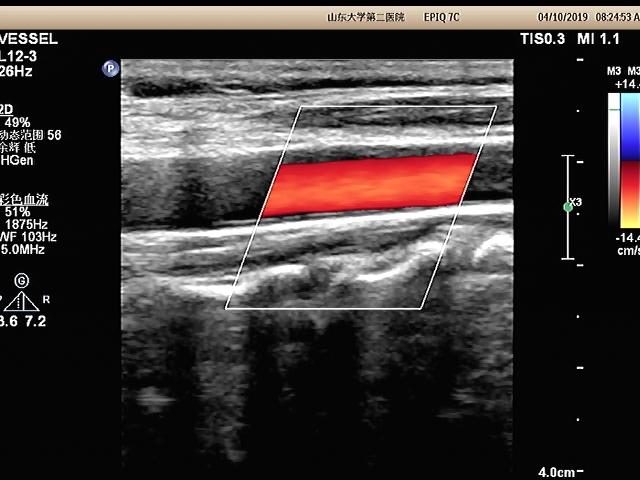

Supplement: Supplementary file 1 [file Data_Sheet_1.ZIP › CONTROLdate1/78.1.JPG]

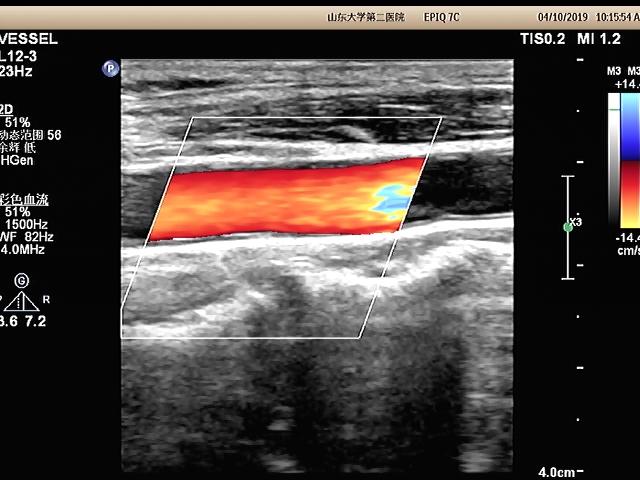

Supplement: Supplementary file 1 [file Data_Sheet_1.ZIP › CONTROLdate1/79.1.JPG]

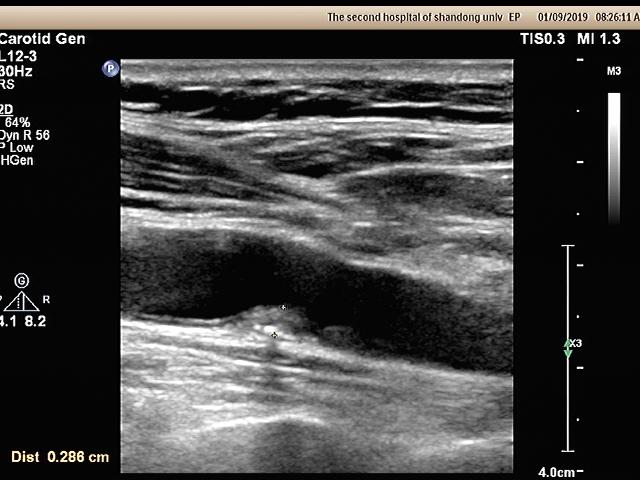

Supplement: Supplementary file 1 [file Data_Sheet_1.ZIP › CONTROLdate1/8.1.JPG]

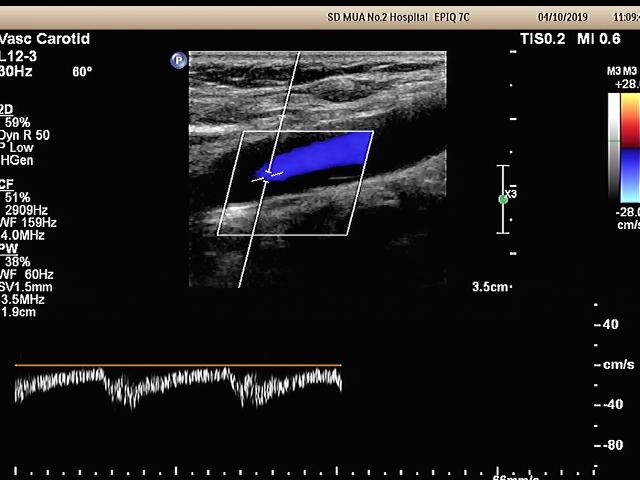

Supplement: Supplementary file 1 [file Data_Sheet_1.ZIP › CONTROLdate1/80.1.JPG]

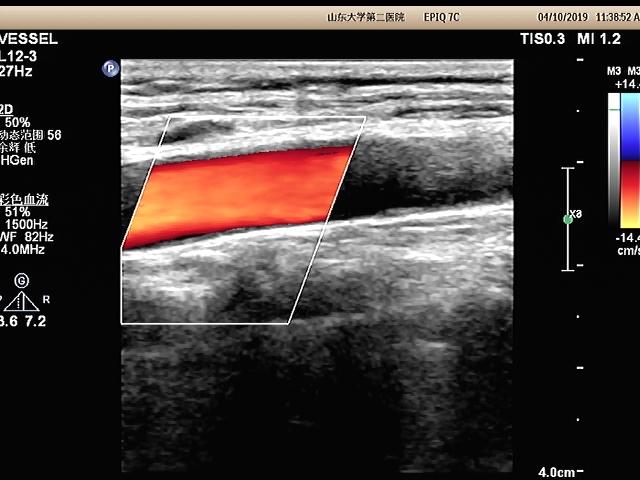

Supplement: Supplementary file 1 [file Data_Sheet_1.ZIP › CONTROLdate1/81.1.JPG]
